# Genomic analysis of NAC transcription factors in banana (*Musa acuminata*) and definition of NAC orthologous groups for monocots and dicots

**DOI:** 10.1007/s11103-013-0169-2

**Published:** 2014-02-26

**Authors:** Albero Cenci, Valentin Guignon, Nicolas Roux, Mathieu Rouard

**Affiliations:** Bioversity International, Commodity Systems and Genetic Resources Programme, Parc Scientifique Agropolis II, 1990 Boulevard de la Lironde, 34397 Montpellier Cedex 5, France

**Keywords:** Comparative genomics, NAC transcription factors, Phylogenetic analysis, Gene family, Expert annotation, Gene duplication

## Abstract

Identifying the molecular mechanisms underlying tolerance to abiotic stresses is important in crop breeding. A comprehensive understanding of the gene families associated with drought tolerance is therefore highly relevant. NAC transcription factors form a large plant-specific gene family involved in the regulation of tissue development and responses to biotic and abiotic stresses. The main goal of this study was to set up a framework of orthologous groups determined by an expert sequence comparison of NAC genes from both monocots and dicots. In order to clarify the orthologous relationships among NAC genes of different species, we performed an in-depth comparative study of four divergent taxa, in dicots and monocots, whose genomes have already been completely sequenced: *Arabidopsis thaliana*, *Vitis vinifera*, *Musa acuminata* and *Oryza sativa*. Due to independent evolution, NAC copy number is highly variable in these plant genomes. Based on an expert NAC sequence comparison, we propose forty orthologous groups of NAC sequences that were probably derived from an ancestor gene present in the most recent common ancestor of dicots and monocots. These orthologous groups provide a curated resource for large-scale protein sequence annotation of NAC transcription factors. The established orthology relationships also provide a useful reference for NAC function studies in newly sequenced genomes such as *M. acuminata* and other plant species.

**Electronic supplementary material:**

The online version of this article (doi:10.1007/s11103-013-0169-2) contains supplementary material, which is available to authorized users.

## Introduction

Identifying the molecular mechanisms underlying tolerance to abiotic stresses is important in crop breeding. A comprehensive understanding of the gene families associated with drought tolerance is therefore highly relevant. The NAC gene family is one of the largest groups of plant transcription factors (TFs), which is known to regulate biotic and abiotic stress-responses such as osmotic stress and various plant developmental processes. NAC proteins are plant-specific TFs, and the NAC family has been recently reviewed by Puranik et al. (2012). NAC genes were originally characterized in a petunia NAM mutant (Souer et al. 1996) and then in *Arabidopsis* CUC (Aida et al. 1997) and ATAF mutants (GenBank accession numbers X74755 and X74756). Two parts can be distinguished in the structure of NAC proteins: the NAC domain (InterPro IPR003441), in the N-terminal region, subdivided in five well-conserved subdomains (A-E); and the transcription regulatory regions (TRRs), in the C-terminal region, which is very variable in sequence and in length. The NAC domain is involved in dimerization and DNA binding, whereas the TRR region plays the role of transcription activator or repressor (Puranik et al. 2012). Evolutionary studies have been done on NAC genes for all major groups of land plants, and it has been shown that some NAC subfamilies were already present in early diverged land plants (Zhu et al. 2012).

Whole genome analyses of the NAC gene family have been performed in several species (*Arabidopsis thaliana*, *Oryza sativa*, *Vitis vinifera*, *Populus trichocarpa, Glycine soja*, *Setaria italica*) (Ooka and Satoh 2003; Fang et al. 2008; Nuruzzaman et al. 2010; Wang et al. 2013; Hu et al. 2010; Le et al. 2011; Puranik et al. 2013), as well as in a subset of viridiplantae species (Zhu et al. 2012). The number of NAC members at the genome level varies with the studied species, from 30, in the early divergent land plants, to more than 160 in *P. trichocarpa.* NAC genes have been classified using phylogenetic analyses in a variable number of groups and subgroups according to the species. The comparison of NAC sequences of *A. thaliana* and *O. sativa* have shown that some NAC members in these two species derived from common ancestors that existed before monocots and dicots diverged (Nuruzzaman et al. 2010).

Whole genome duplications (WGDs) are an important evolutionary feature of plant genomes. Most plant taxa have experienced at least one WGD during their evolution (Van de Peer et al. 2009). A consequence of a WGD is the doubling of all genes. After a WGD event, genomes start to lose (by deletion or pseudogenization) the redundant copies of most of their genes, in a long evolutionary process (fractionation), but some duplicated copies are retained and fixed with modified functional properties. Moreover, differences in gene retention according with their function have been reported (Blanc and Wolfe 2004; Maere 2005). Consequently, in a multigenic family, the number of members in species that experienced independent WGD events can be highly variable. This variability is correlated with the number of WGDs that their genomes experienced in their evolution, the time elapsed from these events, the evolution rate and other evolutionary factors specific to each taxon.

The main goal of this study was to set up a framework of Orthologous Groups (OGs) determined by an expert sequence comparison of NAC genes from both monocots (*O. sativa* and *Musa acuminata*) and dicots (*V. vinifera* and *A. thaliana*). *A. thaliana* and *V. vinifera* were chosen as dicot representatives because the former is a model plant species, while the genome of the latter contains a low number of NAC genes and appears to evolve more slowly than genomes of other dicot taxa (Cenci et al. 2010; 2013; Yue et al. 2010). *O. sativa,* belonging to the Poales clade, was selected as a monocot model species. Instead of another gramineae, we chose *M. acuminata,* a member of the Zingiberales clade of monocots which diverged early from the Poales clade. Moreover, the sequence of the *M. acuminata* DH Pahang genome was recently published and its analyses uncovered one ancient and two more recent WGDs (D’Hont et al. 2012). In banana, six members of the NAC family involved in fruit ripening have been comprehensively characterized (Shan et al. 2012). Among the species considered in the present study, *O. sativa* and *M. acuminata* experienced three independent WGDs in the evolution of their genome (D’Hont et al. 2012). *V. vinifera* and *A. thaliana* underwent a genome triplication (ɣ WGD, common to all the core eudicots) (Jaillon et al. 2007) and the *A. thaliana* genome experienced two additional and more recent WGDs (Van de Peer et al. 2009). WGDs and gene retention lead to multigenic gene families in which OG detection using automatic approaches is challenging. The members belonging to each OG that was defined in this process are supposed to derive from the same ancestor gene existing before the divergence of monocots and dicots, and possibly to share the same function. Our classification intends to be a reference for all monocot and dicot species, to facilitate the identification of the most interesting NAC genes in species for which functional information is lacking.

## Methods

### Sequence retrieval

The sequences of NAC protein-coding genes were extracted from gene family database GreenPhylDB (Rouard et al. 2011) based on the presence of the NAM (No apical meristem) InterPro domain (IPR003441). Excluding splice forms, 82, 111, 146 and 172 sequences were selected for *V. vinifera, A. thaliana*, *O. sativa*, and *M. acuminata*, respectively.

Datasets from *V. vinifera, A. thaliana*, and *O. sativa* were compared with sequences analysed in previous studies. No additional NAC members were found for *V. vinifera* [74 members (Wang et al. 2013)], although only 69 sequences were considered in this study. Five sequences were excluded from the analyses: VvNAC35 and VvNAC38 because they are not NAC but VOZ transcription factors; VvNAC72 because it was identified as a cellulose synthase-like protein G3; and VvNAC50 and VvNAC66 because they are likely pseudogenes.

Among the 105 *A. thaliana* NAC members analysed by Ooka and Satoh (2003), splice forms of five genes were eliminated (ANAC021/022, 034/035, 038/039, 050/051, and 079/080). Among the GreenPhylDB-extracted sequences, after the eliminating the NAC pseudogenes, three sequences (At3g12910.1, At3g12977.1, At4g35580.2) which were not included in the study of Ooka and Satoh (2003) were added to the *A. thaliana* NAC members in this study. Comparisons between *O. sativa* NAC sequences extracted from GreenPhylDB with the 138 and the 151 members found by Fang et al. (2008) and Nuruzzaman et al. (2010), respectively, provided 165 independent sequences for our study. The previously reported *M. acuminata* NAC sequences had all been scanned for the presence of the conserved NAC domain using interproscan searches (http://www.ebi.ac.uk/Tools/pfa/iprscan/). The sequences used in the present study are provided in Online Resources 1.

### Expert annotation of NAC sequences

Since the *M. acuminata* NAC sequences were the result of an automatic annotation, an expert revision of the gene structure of this family was conducted. In order to detect additional InterPro protein domain IPR003441 not included in the annotated genes, a tBlastn search was performed on the *M. acuminata* pseudo-chromosome sequences with a sample of NAC genes. Banana sequences were curated using the Banana Genome Hub (Droc et al. 2013) and more specifically with the Artemis software (Carver et al. 2008) connected to the Community Annotation System (Guignon et al. 2012). Modifications of the gene structures were based on transcripts, when available, and protein similarity with published NAC genes of other plant species. The revised annotations are publically available via the Banana Genome Hub. Some modifications of published NAC annotations for *A. thaliana*, *V. vinifera* and *O. sativa* were also performed. The modified sequences are reported in Online Resources 1 and a concise description of modifications is provided in Online Resources 2.

### Analysis of NAC duplication in *Musa* genome

NAC genes separated by not more than 10 other genes were considered as tandem duplications. NACs generated by segmental duplication of the *Musa* genome were determined by the analysis of the output file (Online Resources 3) of the CoGe SynMap program (http://genomevolution.org/CoGe/SynMap.pl) obtained using a default parameter (Lyons et al. 2008).

### Expert inference of the OGs

**Fig. 1:**
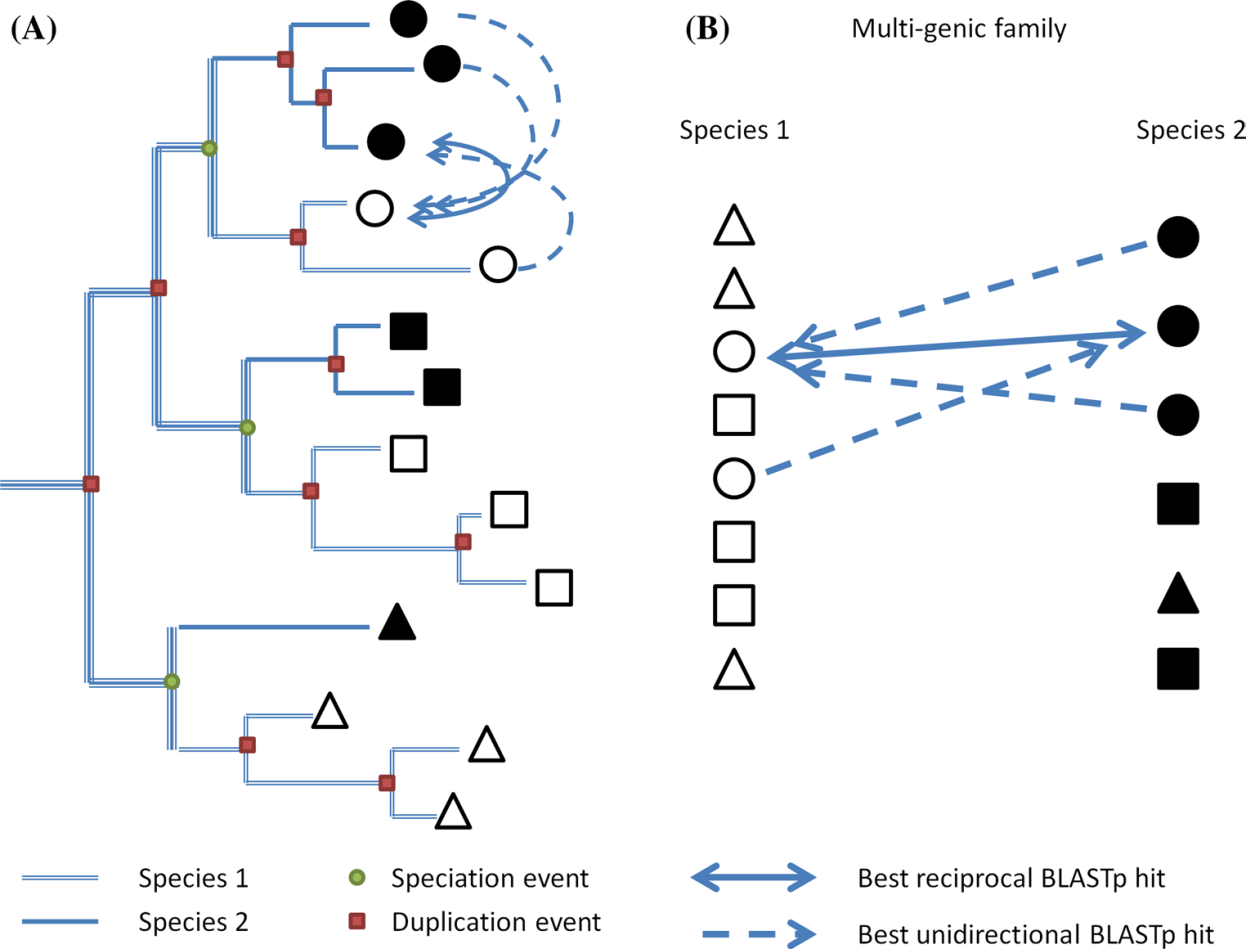
Inference of the orthologous groups with similarity-based approach. *Empty* and *filled shapes* indicate orthologous copies in two different species. **a** Schematic representation of the amplification of a multi-copy family in two species. *Arrows* connect copies of an OG in a species with the less divergent copy of the other species. **b** Expected Blastp results for an OG. *Arrows* connect copies of a species with the best Blastp hit in the other species

Orthologous group (OG) reconstruction was based on sequence similarity inferred from protein–protein BLAST analyses (analyses performed with default Blastp parameters) that were all carefully examined by human expertise (Fig. 1). The method used is based on two assumptions. Firstly, the best Blastp hit indicates the lowest protein distance from the query sequence. Secondly, for each member of the query species, the smallest protein distance will be found with an orthologous gene of the subject species, although, this second assumption cannot be true in all circumstances, particularly for rapid sequence evolution of the orthologous gene(s). However, since all the NAC genes have a similar function (regulation of gene transcription), dramatic differences in their evolution rates are expected to be infrequent.

The orthologous inference was performed in two steps. First, all the NAC amino acid sequences of *A. thaliana*, *M. acuminata* and *O. sativa* were grouped with the *V. vinifera* NAC member having the higher Blastp score. Then, Blastp analyses were performed for all the other “species by species” combinations to verify the highest sequence-similarity among the sequences of each group. When inconsistencies were found (i.e. the best NAC protein found for the three species belonged to two different groups) the majority rule was applied and the query sequence included inside the group containing the two best hits. The Blastp analyses of *V. vinifera* NAC sequences also allowed grouping those genes never found having best Blastp hits with the other species. Sequences having low Blastp scores with other species (see results) were considered species-specific and not considered in subsequent comparative analyses.

### OrthoMCL clustering

The whole set of NAC sequences included in the OGs were clustered using OrthoMCL (Li et al. 2003) after Blastp all vs all (e-value 1e−10). The inflation parameter was gradually increased from 1.5 (default) to 14, to vary levels of granularity and until we reached the number of OGs identified by the expert annotation. Then, the resulting groups were compared to check if the manually obtained results were reproducible by a common strategy for orthology group detection (i.e. OrthoMCL).

### Phylogenetic analyses

Phylogenetic analyses were performed using an in-house phylogenetic workflow powered by Galaxy (available at http://gohelle.cirad.fr/galaxy/u/reviewer/w/greenphyl-phylogenomic-analysis-workflow) that reproduces the main steps of the GreenPhyl pipeline. These steps included: multiple alignment (MAFFT v.6 (Katoh and Toh 2008)); masking (GBLOCKS (Talavera and Castresana 2007)). Gene tree reconstruction was performed using PhyML (Guindon and Gascuel 2003) with tree improvement with best of NNI and SPR methods with aLRT support (Anisimova and Gascuel 2006) steps. In order to optimize the resolution of the global phylogeny, we used ProtTest v3 (Darriba et al. 2011) to define the best substitution model. Since the JTT model resulted slightly better than LG, it was chosen for the phylogeny analysis of the whole set of NAC sequences. The gene trees were reconciled using RAPGreen (Dufayard et al. 2005) using a species tree that is compliant with NCBI taxonomy (Online resources 4). All the multiple alignments and gene trees were deposited in Treebase (http://purl.org/phylo/treebase/phylows/study/TB2:S14688)

## Results

### Number of NAC genes in *M. acuminata* genome

Among the 36,542 automatically predicted genes in the *M. acuminata* genome, 172 contained the InterPro protein domain IPR003441. To improve their structural annotation, all these sequences were manually curated, as necessary. Additional genomic regions containing the domain IPR003441 were also detected and analyzed. Globally, our analysis resulted in 167 potentially functional NAC genes (Online Resources 1) that we retained for this study. Five pseudogenes and a number of remnants were also found in the *M. acuminata* genome. We then refined the selection of NAC sequences for other species, taking into account previous publications dedicated to NAC transcription factors (see “Methods”).

### NAC member duplication in *M. acuminata*

The banana genome contains the largest number of NAC genes among the already sequenced genomes of angiosperms. Despite the large number of NAC genes observed, only twelve tandem duplication regions (involving 27 genes) were detected in *M. acuminata*. By contrast, at least 18 segmental duplications (involving 43 NAC genes) were detected by CoGe SynMap (Online Resources 3), that originated from one of the last two WGDs that occurred for the *Musa* genome (D’Hont et al. 2012).

### Expert orthologous grouping of NAC sequences

The *V. vinifera* NAC sequences were chosen as a reference for two main reasons: (a) *V. vinifera* proteins appear to have a slower evolution rate than other dicots (Cenci et al. 2010; 2013; Yue et al. 2010), and (b) its genome experienced the lowest number of WGDs after the monocot/dicot lineage divergence (Bowers et al. 2003; Jaillon et al. 2007; Tang et al. 2010; D’Hont et al. 2012; Paterson et al. 2009) which is the probable main reason for its low number of NAC members.

Among the 103 *A. thaliana* NAC members, 19 displayed very low Blastp scores (lower than 170) with the best hit of the *V. vinifera* protein database and with the other two analysed species. Consequently, only the 84 NAC members (showing Blastp score higher than 250) were considered for the grouping. Similarly, 73 *Oryza sativa* NAC sequences showed very low similarity with NACs of other analysed species and were not considered in the orthologous grouping.

Finally, among the 167 *M. acuminata* NAC sequences, those five having a very low score with the best *V. vinifera* hit (less than 130) were discarded, and the remaining 162 with BLAST scores higher than 200 were retained for the orthologous grouping.

Thus, 40 OGs containing NAC members were obtained (Table 1). The nomenclature we used for OGs is based on the eight *V. vinifera* clusters (Wang et al. 2013) with a letter to distinguish different groups inside the clusters (Table 1). For example, among the nine *V. vinifera* NAC sequences included in cluster 1, eight OGs were named from 1a to 1h (being two sequences, VvNAC05 and VvNAC11, included in the OG 1 h). Similarly, cluster 5 (containing eleven *V. vinifera* NAC sequences) was subdivided into three OGs: 5a (containing VvNAC01, VvNAC07 and VvNAC73), 5b (VvNAC25, VvNAC51-VvNAC55 and VvNAC71) and 5c (VvNAC67). Two of these groups did not contain any *A. thaliana* sequence (3b, 7f), whereas three other groups did not contain any monocot sequences (3e, 4f, and 5c). An additional OG was obtained with one *O. sativa* and two *M. acuminata* very closely related sequences; this monocot-specific group was named MS (Table 1). Four sequences (Os08g33670.1 and Os11g03310.1 close to the sequences of OGs 1; VvNAC42 and VvNAC45 close to OGs 3c or 3d) could not be assigned due to very similar Blastp scores with sequences of different OGs.

**Table 1:** List of the 40 OGs of the NAC gene family based on sequence analysis in four angiosperm species

Orthologous group	*V. vinifera*	*A. thaliana*	*O. sativa*	*M. acuminata*	Function
1a	**VvNAC56**	**ANAC074**	Os02g41450.1 Os02g56600.1 Os03g01870.1 **Os04g43560.1** Os10g33760.1	Achr1T10860 Achr1T20530 Achr5T18670 Achr6T36550 Achr8T18980 Achr11T00880 Achr11T22760 AchrUn_randomT17260 AchrUn_randomT02760	MaNAC6 (Achr11T00880), banana fruit ripening (Shan et al. 2012)
1b	**VvNAC33**	**ANAC021/022** ANAC_ At3g12977.1	Os02g06950.1 Os04g52810.1 **Os06g46270.1** Os08g10080.1 Os12g41680.1	Achr3T23360 Achr5T00500 Achr5T26640 Achr6T30050 Achr6T31350 Achr8T07120 Achr8T33310 Achr9T10210 Achr9T26140 Achr11T25720	NAC1 (ANAC21/022) Root development (Guo et al. 2004); Os12g41680, abiotic stresses (Nuruzzaman et al. 2012); MaNAC5 (Achr9T26140), banana fruit ripening (Shan et al. 2012)
1c	**VvNAC65**	**ANAC038/039**	**Os09g32260.1**	Achr6T01770 **Achr7T19680** Achr8T24680	
1d	**VvNAC16**	ANAC054 **ANAC098**	**Os06g23650.1**	**Achr10T22350** Achr10T26180	CUC1 and CUC2 (ANAC054 and 098), shoot apical meristem development (Takada et al. 2001)
1e	**VvNAC14**	**ANAC031**	**Os08g40030.1**	**Achr9T20090**	CUC3 (ANAC031), shoot apical meristem development (Hibara et al. 2006)
1f	**VvNAC06**	**ANAC058**	**Os03g42630.1**	Achr8T18420 Achr9T00570^a^ **Achr10T19900**	
1 g	**VvNAC61**	ANAC046 **ANAC087**	Os01g01470.1 Os01g29840.1 Os03g21030.1 Os07g48550.1 Os11g03310.1 Os11g03370.1^b^ Os12g03050.1	Achr3T09520 Achr3T18020 Achr5T07600 Achr6T08600 Achr6T32290 Achr7T18330 Achr8T21470 Achr9T16920 Achr10T05070	OsNAC45 (Os11g03370), drought and salt tolerance (Zheng et al. 2009); Os11g03370, Os12g03050, virus infection (Nuruzzaman et al. 2010)
1 h	VvNAC05 **VvNAC11**	ANAC059 ANAC079/080 ANAC092 ANAC100	Os02g36880.1 **Os04g38720.1**	Achr6T30570 Achr7T11500 Achr9T27530	AtNAC2 (ANAC059), salt stress response and lateral root development (He et al. 2005); ANAC092, salt stress (Balazadeh et al. 2010); OsNAC2 (Os04g38720) Shoot branching (Mao et al. 2007); MaNAC3 (Achr9T27530), banana fruit ripening (Shan et al. 2012)
2a	**VvNAC02** VvNAC22	**ANAC007** ANAC026 ANAC101	**Os02g42970.1** Os04g45340.1 Os06g01480.1	Achr6T36840 Achr7T06640 Achr8T11590 Achr11T03780 Achr11T17510	VND4-6 (ANAC007, 026, 101), vascular development (Kubo 2005)
2b	**VvNAC23**	**ANAC037** ANAC076 ANAC105	Os03g03540.1 Os10g38834.1	Achr8T12100 Achr11T03040	VND1-3 (ANAC037, 076,105), vascular development (Kubo 2005); Os10g38834, drought stress (Nuruzzaman et al. 2012)
2c	**VvNAC63**	**ANAC030**	Os04g59470.1 Os08g01330.1	Achr3T22360 Achr7T23170	VND7 (ANAC030), vascular development (Kubo 2005)
2d	**VvNAC70**	ANAC015 **ANAC070**	**Os04** **g (unannotated, OsI_35493)**	**Achr2T05640** Achr6T28890	Bearskin1-2 (ANAC015, 070), Root cap maturation (Bennett et al. 2010)
2e	**VvNAC68**	**ANAC033**	Os02g15340.1 **Os06g33940.1**	**Achr2T20020** Achr10T14400 Achr10T21750	Sombrero (ANAC033), Root cap maturation (Willemsen et al. 2008)
2f	**VvNAC24** VvNAC49	**ANAC043** ANAC066 ANAC012	Os06g04090.1 **Os08g02300.1**	**Achr3T12230** Achr7T05980 Achr9T24450 AchrUn_randomT21980	NST1-2 (ANAC043-066) and SND1 (ANAC012), Secondary wall thickening (Mitsuda et al. 2005; 2007)
3a	**VvNAC08** VvNAC39	**ANAC002** ANAC032 ANAC081 ANAC102	**Os01g66120.1** Os05g34830.1 Os11g08210.1	Achr3T18990 Achr6T17720 Achr6T18720 Achr6T25380 Achr7T23250 Achr10T04570	ATAF1 (ANAC002) Drought stress responses (Hu et al. 2006); ATAF1-2 (ANAC002-081), repressor of pathogenesis-related proteins (Delessert et al. 2005; Wang et al. 2009); OsNAC6 (SNAC2), 5, 52 (Os01g66120, Os11g08210, Os5g34830) Abiotic stress (Ohnishi et al. 2005; Nakashima et al. 2007; Hu et al. 2008: Takasaki et al. 2010; Gao et al. 2009)
3b	VvNAC44		Os01g60020.1 Os03g60080.1 Os07g12340.1	Achr4T02390 Achr4T10310 Achr5T07590 Achr6T32330 Achr7T21780 Achr10T29200	OsNAC19 (0s3g60080), response to infection by *M. grisea* (Lin et al. 2007); SNAC1 (Os3g60080) drought stress (Hu et al. 2006
3c	**VvNAC60** VvNAC26	ANAC047 ANAC029	Os01g01430.1 **Os03g21060.1** Os05g34310.1 Os07g48450.1 Os11g03300.1 Os12g03040.1	Achr3T18010 Achr4T02380 Achr6T32320 Achr7T21770 Achr9T04960 Achr10T12860 AchrUn_randomT17360	AtNAP (ANAC029), leaf senescence (Guo and Gan 2006); OsNAC10 (Os11g03300) Drought tolerance (Jeong et al. 2010); Os11g03300, Os12g03040, reponse to *Magnaporte grisea* infection, (Sun et al. 2013)
3d	VvNAC03 VvNAC43^a^ VvNAC18	ANAC018 ANAC025 ANAC056	Os07g37920.1	Achr1T08860 Achr9T19520	Os07g37920, senescence (Distelfeld et al. 2012)
3e	VvNAC17	ANAC072 ANAC019 ANAC055			ANAC072 (RD26), 019 and 055, Drought tolerance (Tran et al. 2004); ANAC019, ANAC055, Defense disease, Jasmonate pathway (Bu et al. 2008)
4a	**VvNAC64**	**ANAC028** ANAC045 ANAC086	**Os03g02800.1**	Achr1T02820 **Achr8T13430** Achr11T01400 Achr11T16860 AchrUn_randomT08190	RIM1 (Os3g02800) virus resistance; Jasmonate pathway signalling (Yoshii et al. 2010)
4b	**VvNAC57**	**ANAC057**	**Os09g38000.1** Os09g38010.1	**Achr3T13880** Achr9T23940	
4c	**VvNAC13**	**ANAC071** ANAC011 ANAC096	**Os10g42130.1**	Achr3T18070 Achr5T03360 **Achr5T18140** Achr11T17780	
4d	**VvNAC20** VvNAC21	ANAC050 ANAC051/052 ANAC053 ANAC077 **ANAC078**	**Os02g57650.1** Os08g44820.1	Achr2T04010 Achr9T20400 Achr9T23580 AchrUn_randomT07620	
4e	VvNAC48	ANAC082 ANAC103	Os05g35170.1 Os_AK068153	Achr7T23330 Achr10T04530 Achr11T09000	VNI1 (ANAC082), vascular development (Yamaguchi et al. 2010)
4f	VvNAC69	ANAC020			
4 g	**VvNAC15**	ANAC013 ANAC016 **ANAC017**	**Os09g32040.1**	**Achr8T24280** AchrUn_randomT11980	
5a	**VvNAC01** VvNAC07 VvNAC73	ANAC041 ANAC084 ANAC097 **ANAC083**	Os01g70110.1 Os08g42400.1 Os09g33490.1 Os11g31330.1 **Os12g29330.1**	Achr2T21110 Achr3T18680 Achr6T16560 Achr6T20870 Achr6T23840 Achr7T22480 Achr10T16940 Achr10T11910 AchrUn_randomT24680	VNI2 (ANAC083), vascuolar development, salt tolerance, leaf senescence (Yamaguchi et al. 2010; Yang et al. 2011; Seo and Park 2011)
5b	**VvNAC25** VvNAC51 VvNAC52 VvNAC53 VvNAC54 VvNAC55 VvNAC71	**ANAC014** ANAC062 ANAC091 ANAC_ At4g35580	Os06g01230.1 **Os08g06140.1**	Achr2T11810 Achr3T00330 Achr4T29170 Achr11T07800 AchrUn_randomT04060	TIP (ANAC091), virus interacting (Ren et al. 2000); NTL6 (ANAC062) drought stress (Kim et al. 2012); ntm2 (ANAC069) salt stress (Park et al. 2011); NTL9 (ANAC_At4g35580) salt stress (Yoon et al. 2008)
5c + MS	VvNAC67	ANAC040 ANAC060 ANAC089	Os01g15640.1	Achr5T23620 Achr6T03200	NTL8 (ANAC040), regulation of salt-responsive flowering (Kim et al. 2007)
6a	**VvNAC40**	**ANAC034**	**Os01g66490.1** Os05g34600.1 Os08g02160.1	Achr7T23650 Achr3T21690 Achr5T17060 Achr10T04320 Achr10T10690 Achr11T08970 Achr11T22590 Achr11T26450	LOV1 (ANAC034) Cold response, photoperiod pathway (Yoo et al. 2007)
6b	VvNAC10 **VvNAC27**	**ANAC009** ANAC094	**Os08g33910.1** Os02g51120.1	**Achr6T02680** Achr10T08120	FEZ (ANAC009), Root cap maturation (Willemsen et al. 2008); ONAC063 (Os08g33910) salt stress (Yokotani et al. 2009)
6c	VvNAC28 VvNAC29 VvNAC30 VvNAC31 VvNAC32 **VvNAC36**	ANAC_ At3g12910.1 **ANAC042**	**Os03g56580.1** Os07g04560.1 Os12g43530.1	Achr4T23030 Achr4T32010 Achr5T02170 Achr6T31585 Achr7T00860 **Achr9T10040** Achr10T08420	ANAC042, heat stress (Shahnejat-Bushehri et al. 2012) and pathogen infection (Saga et al. 2012); MaNAC2 (Achr6T31585), MaNAC4 (Achr7T00860), banana fruit ripening (Shan et al. 2012); Os10g38834, drought stress (Nuruzzaman et al. 2012)
7a	**VvNAC34** VvNAC37	ANAC010 **ANAC073**	**Os01g48130.1** Os05g48850.1	Achr2T09080 Achr4T02730 **Achr6T27000**	MaNAC1 (Achr6T27000), banana fruit ripening (Shan et al. 2012)
7b	**VvNAC19**	**ANAC075** ANAC099	**Os01g09550.1** Os05g10620.1 Os06g36480.1	**Achr2T16590** Achr4T30940 **Achr6T05480** Achr7T09510 Achr10T27600	
7c	**VvNAC12**	**ANAC008**	**Os06g15690.1**	**Achr3T07330** Achr6T11230^a^	SOG1 (ANAC008), response to gamma radiation (Yoshiyama et al. 2009); Os06g15690 drought stress (Nuruzzaman et al. 2012)
7d	**VvNAC59**	**ANAC044** ANAC085	Os04g40140.1 **Os02g38130.1**	Achr9T01880 **AchrUn_randomT17050**	Os04g477300 (Os04g40140) boron-toxicity tolerance (Ochiai et al. 2011); Os0238130, viral infection (Nuruzzaman et al. 2010)
7e	VvNAC47 **VvNAC58**	**ANAC104**	**Os02g34970.1** Os04g35660.1	Achr6T17670 Achr6T18640 Achr6T25790 **Achr10T10790**	XND1 (ANAC104), lignocellulose synthesis (Zhao et al. 2007); Os02g34970) drought stress; viral infection (Nuruzzaman et al. 2010; 2012)
7f	**VvNAC09**		**Os10g21560.1**	Achr2T06610 Achr4T07148 **Achr7T26050**	
8a	VvNAC04 VvNAC41	ANAC036	Os03g04070.1 Os06g51070.1	Achr1T02710 Achr3T00560 Achr3T14720 Achr5T19060 Achr7T04030 Achr11T01320 AchrUn_randomT08220	
8b	VvNAC46 VvNAC62 **VvNAC74**	ANAC061 **ANAC090**	Os01g64310.1 **Os05g37080.1** Os11g05614.1 Os11g45950.1^a^ Os12g05990.1	Achr6T19400 Achr8T01410 Achr9T29750 Achr10T04720	Os11g05614, virus infection (Nuruzzaman et al. 2010)

The last column contains information on known functions for genes of the relative groups. Sequences showing best reciprocal Blastp hits are indicated in bold. Os08g33670.1 and Os11g03310.1 were close to the sequences of cluster 1, but they could not be assigned to a specific group. VvNAC42 and VvNAC45 could not be resolved between groups 3c or 3d

^a^Putative pseudogenes

^b^ Os11g03310.1 was assigned to the group 1g based on the phylogenetic analysis results

### OrthoMCL analysis

**Fig. 2:**
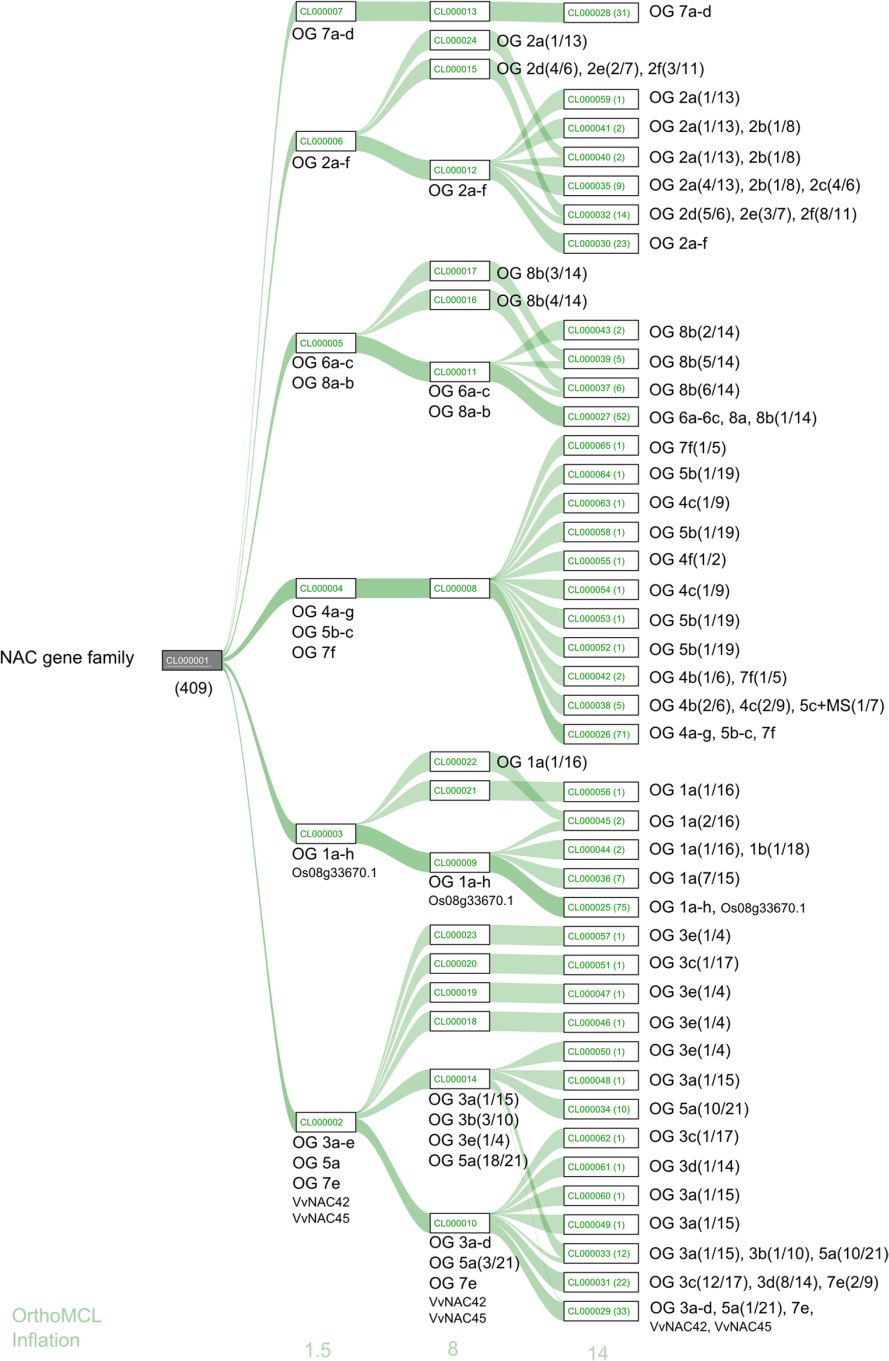
Comparison between expert versus OrthoMCL automatic analysis for OG detection. Clusters obtained by OrthoMCL with three increasing inflation parameter and the OG member included in each cluster

To verify whether our orthologous inferences could be reproduced automatically in such a very large gene family as the NAC, OG sequences were submitted to OrthoMCL clustering, using a panel of growing inflation parameters (from 1.5 to 14) that increase the cluster stringency. With the lowest stringency, six clusters were obtained (Fig. 2 and Online Resources 5) containing NAC members of variable numbers (from 4 to 10) of those OGs determined by expert comparison. None of these OGs was split in different OrthoMCL clusters. The increase in stringency did not allow OG differentiation, but with higher inflation parameters, single NAC sequences were isolated or OGs split in different clusters (Fig. 2, Online Resources 6-10). Only the cluster containing all NAC sequences of OGs 7a-d was maintained regardless of stringency level (Fig. 2, Online Resources 5-10).

### Structural analysis of NAC genes

**Fig. 3:**
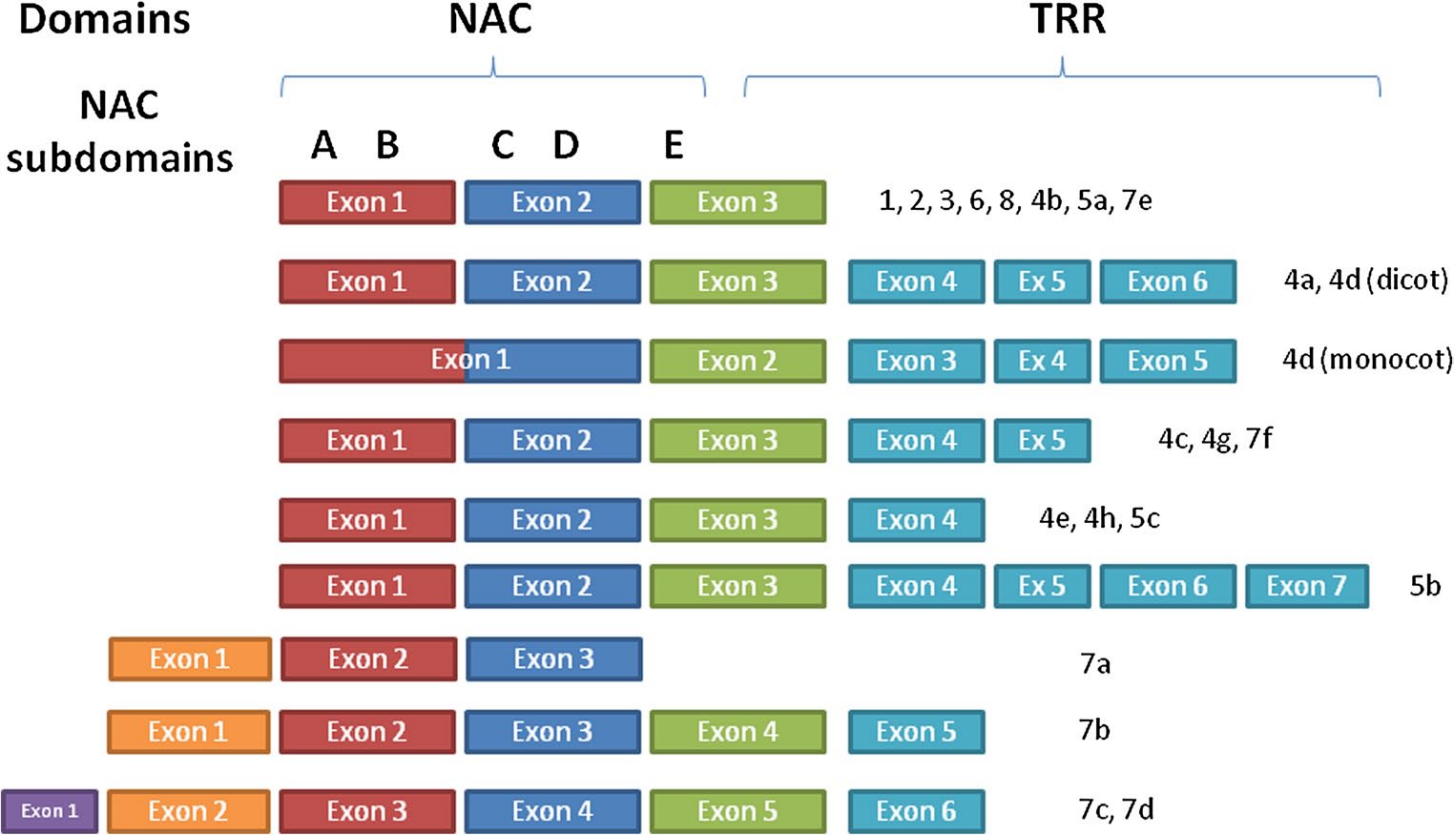
Exon/intron structure of NAC genes according to the orthologous groups

In addition to the sequence similarity, the exon/intron structure of the NAC genes was comprehensively analysed. Almost all NAC genes of OGs 1, 2, 3, 6 and 8 were found to have very similar structures, with three exons that aligned well (Fig. 3). The first exon contains A and B subdomains (Kikuchi et al. 2000) and ends at the first nucleotide of the first codon after the B subdomain; the second exon contains the C and D subdomains and ends at the third nucleotide of a codon; the third exon begins with the E subdomain and contains all the C-terminal region of the gene that includes the TRR. A few exceptions to this typical structure were observed in *A. thaliana* and *O. sativa* due to intron loss or, in other words, exon merging. In *A. thaliana*, only ANAC066 and ANAC101 genes are composed of two exons, because the second intron has been lost. In *O. sativa*, Os06g23650.1, Os08g40030.1 and Os11g05614.1 lost the first intron, Os03g42630.1, Os01g01470.1 and Os01g29840.1 lost the second intron, and Os12g05990.1 and Os06g51070.1 lost both introns. Moreover, all the three NAC members of the 3b group lost the first intron, whereas only the Os07g12340.1 gene lost also the second intron resulting in a mono-exonic structure.

Genes included in OGs 4, 5 and 7 have more variable numbers of exons. OGs 4a-h and 5a-c have the typical position for first two introns, but additional introns could be present in the TRR, varying from none (OGs 4b and 5a) to four (OG 5b). In monocot sequences of OG 4d, the first intron was lost and, consequently, the first exon contains the first four subdomains (Fig. 3).

OGs 7a-d have particular features. E subdomains are not recognizable and the beginning of the gene has a different structure. OGs 7a-b genes have an additional exon at the beginning, so A-B subdomains are included in the second exon (that ends at the same position as the first exon of other groups), and third exon contains the C-D subdomains (Fig. 3). These two OGs can be differentiated according to their structure. Genes of OG 7a have only three exons; in OG 7b, genes are longer and have three additional exons in the TRR. Genes in OGs 7c and 7d have two additional exons at the beginning, the first with a very small coding region (3–4 amino acids). Consequently the A-B subdomains are in the third exon, whereas the C-D subdomains are in the fourth one. The fifth and sixth exons contain the TRR (only Os04g40140.1 has 7 exons due to an additional intron in the fourth exon). Finally, genes of OG 7e have the typical structure with three exons and genes of group 7f have two additional exons located in the TRR (with the exception of GSMUA_Achr2T06610.1 that merged the last two exons).

### Phylogenetic analyses

**Fig. 4:**
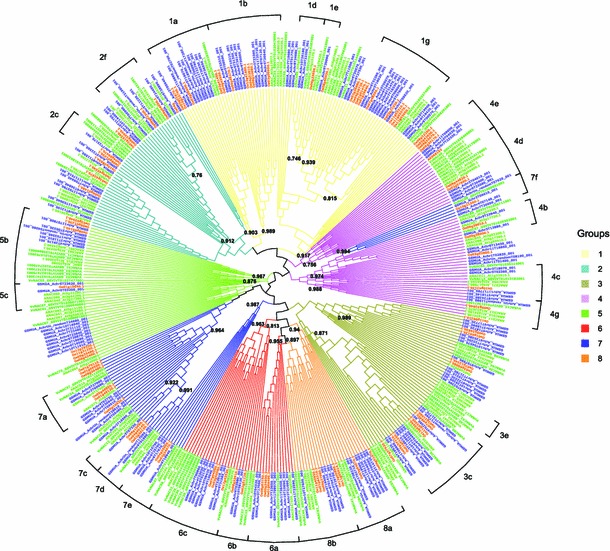
Maximum Likelihood phylogenetic tree of NAC proteins. Phylogenetic analysis was carried with protein sequences from *A. thaliana* (ARATH), *V. vinifera* (VITVI), *O. sativa* (ORYSA) and *M. acuminata* (MUSAC) as described in the “Methods”. Branch support values correspond to approximate likelihood ratio test (a-LRT) results. The 26 clusters supporting the grouping were indicated on the figure with the numbering proposed in this work. Leaf colors of the gene tree are colored according to their species. PhyloXML format of this gene tree and phylogenetic trees of the groups are provided as Online Resources 4

A phylogenetic analysis was performed with all the sequences included in the OGs (84, 69, 92 and 162 for *A. thaliana*, *V. vinifera*, *O. sativa* and *M. acuminata*, respectively) and a gene tree was obtained (Fig. 4 and Online Resources 11) based on 86 aligned amino acids, all belonging to the NAC domain. Twenty-six clusters were observed that contain all the NAC members of the OGs determined by expert comparison. On the other hand, a number of sequences relationships were not well defined. Two OGs, MS and 5c (specific to monocots and dicots, respectively), were consistent with the phylogenetic results, but these clusters were closely related and formed a unique cluster (aLRT = 0.876). Consequently these two OGs were merged into the 5c OG for subsequent analysis.

In order to verify whether the phylogenetic resolution could be improved using a set of more closely related sequences, we performed additional phylogenetic analyses with the sequences of OGs 2a–f (51 sequences) and 7a–d (33 sequences) which, in the global NAC gene tree, are clustered and sharply isolated from all other NAC but not well resolved in clear sub-clusters (Online Resources 11). The trees were obtained based on 163 and 175 aligned amino acids, respectively, and they contained also positions outside the NAC domain. The phylogenetic trees were perfectly consistent with expertly determined OGs, being all the sequences in OG-specific clusters, with aLRT values spanning between 0.790 and 0.997 (Online Resources 12).

Finally, phylogenetic analyses were performed for each expertly identified OG; since *A. thaliana* or monocots NAC sequences were lacking in four OGs (3b, 3e, 4f and 7f), only thirty-six trees were built (Online Resources 13). Among the 36 phylogenetic trees obtained, 31 showed the expected dicot and monocot clustering. In two additional trees, a sequence of *M. acuminata* (Achr7T17500, OG 1 h) and two sequences of *A. thaliana* (ANAC084 and ANAC097, OG 5a) occupy unexpected positions; all these three sequences were characterized by very long branches (Online Resources 13). In the remaining three trees, one was unresolved (3c) whereas in the other two (3d and 1d) monocot and dicot sequences were not separated into phylum-specific clusters. For a given species, when more than one sequence is included in an OG, species-specific clusters are often observed, which indicates gene amplifications took place after the lineage divergence. On the other hand, in few cases sub-clusters containing dicot and/or monocots (e.g. group 2a, Online Resources 13) could indicate gene duplications that occurred before the *Arabidopsis*-*Vitis* and/or *Musa*-*Oryza* lineage divergence.

## Discussion

The phylogenetic position of *M. acuminata* (order Zingiberales, a sister group to the well-studied Poales order) offers the opportunity for a deeper exploration into monocot evolution and into plant genome evolution in general. The present study analysed the evolution of NAC genes, a high copy TF family in the monocots and dicots, the two major lineages of angiosperms.

### *Musa acuminata* NAC

The availability of the *M. acuminata* genome sequence allows global analysis of large gene families such as NAC transcription factors. Automatic gene annotation provides an initial panorama of abundance and chromosome distribution of the members of this gene family. As automatic gene structure annotation can be imprecise, we performed a revision of the members of this large family. We considered 167 genes to be potentially functional, and eliminated from the analyses five pseudogenes predicted by the presence of mutations modifying the typical gene structure and by the lack of observed transcription. However, due to the limited availability of transcriptome data, the lack of functionality of these genes needs to be confirmed, as well as the effective activities of the potentially functional genes. Compared to other species, the *M. acuminata* genome contains a high number of NAC genes. Twelve ancestral genomic regions pre-dating the last two WGDs could be reconstructed by analysis of gene collinearity among *Musa* chromosomes (D’Hont et al. 2012). When the NAC genes were positioned in these ancestral groups, at least 43 genes could be inferred as derived from duplications during one of the last two *Musa* WGDs.

### Orthologous grouping of NAC genes from different species

The orthologous grouping of NAC sequences belonging to four distinctly divergent species was based on similarities inferred by Blastp analysis. Even if Blastp does not provide a distance measure between two sequences, a better Blastp score was assumed to indicate a greater similarity between compared sequences.

Among all the NAC sequences available, a number of sequences showed a very low Blastp score with any NAC of other species. The Blastp score is influenced by the distance between the protein sequences, which in turn is influenced by the genetic distance between the analysed species. The latter in turn is determined by the phylogenetic position and evolution rate of the analysed species. For example, a lower genetic distance is expected between two monocot species than between a monocot and a dicot. Given this, empirically set thresholds of Blastp scores were used to exclude highly divergent sequences from the comparative analysis. Moreover, these highly divergent sequences showed similar Blastp scores with sequences assigned to several OGs that render these sequences unassignable to any OG. Sequences showing low Blastp scores with other species could be the result of species-specific sequence evolution or degradation due to the relaxation of any purifying selective pressure (pseudogenization).

The existence of a large group of *O. sativa*-specific NACs was highlighted in the study of Nuruzzaman et al. (2010). The large majority of these sequences resulted in cluster II in the phylogenetic analysis of Fang et al. (2008). In the light of their species specificity, these sequences were not considered in our comparative study.

More than 400 sequences were manually compared for their similarity with a species by species Blastp analysis. Forty OGs containing NAC sequences from at least two species were obtained. In addition to their being lower in number, NAC sequences of *V. vinifera* were chosen as the reference for comparison because of the reported lower evolution rate of this species compared to other dicots (Cenci et al. 2010; 2013; Yue et al. 2010). The phylogenetic analyses performed on NAC sequences of 36 OGs were consistent with these reports, i.e. the branch lengths of NAC sequences being generally shorter in *V. vinifera* than in *A. thaliana* (Online Resources 13).

Using the full set of sequences, phylogenetic analyses confirmed most of the orthologous grouping results: 26 of the 40 OGs were found consistent with clustering, i.e. all the NACs of a given OG were found in an OG-specific cluster. The other groups could not be resolved in OG-specific clusters, although no inconsistencies were noted with the proposed grouping (i.e. no well-supported clusters containing sequences from different OGs were observed). OGs 7a-d appeared very divergent from the other NACs, which is consistent with their particular structure (additional exons at the beginning) and sequence (lack of E subdomains). The NAC of the OGs 2a-f and 7a-d resulted in two well-defined clusters, but the distribution of NAC in specific OGs was not completely resolved. By performing phylogenetic analysis restricted to the sequences of these OGs, we obtained perfectly consistent clusters with the grouping made by the Blastp reciprocal analysis. The number of amino acid positions in the filtered multiple alignment was approximately twice the number of the ones obtained with the whole set of NAC sequences (86 amino acids). It is likely that the phylogenetic signal was clearer and enabled a better resolution of these clusters than the global analysis.

The phylogenetic analyses performed on NAC sequences of 36 OGs showed congruence with the known species phylogeny (i.e. the monocot/dicot classification), even if some sequences had unexpected positions. These exceptions could be the results of specific high sequence-divergence (as suggested by their long branches in the tree) maybe due to purifying selection relaxation in the presence of redundant copies of genes.

Automated clustering by OrthoMCL with the lowest stringency level provided six clusters containing several manually obtained OGs (spanning from 4 to 10). The increase in stringency did not provide a better resolution of OGs, but tended to isolate more divergent sequences (Fig. 2). NAC sequences appear to be problematic for automatic clustering, mainly because of the large number of OGs and of repeated gene duplications inside each lineage.

The phylogenetic analysis of *O. sativa* NAC (Fang et al. 2008) was extremely consistent with the OGs’ distribution obtained in this study. When several *O. sativa* NAC sequences are included in a given OG, they also appear clustered in the phylogenetic tree obtained by Fang et al. (2008). For example, groups 1a and 1b coincide with the two main sub-clusters of cluster I-2 (NAC1), and the six *O. sativa* NAC sequences of group 1 g were in a specific cluster as well as both NACs of the 1 h group. Some exceptions were observed involving OGs 3c, 7b and 5a. Even if NAC sequences of OGs 6a-c and 8a-b were resolved in five OG-specific clusters, their clusters were mixed in our phylogenetic analyses (performed with both the global sample or limited to the 6 + 8 groups). This is consistent with the findings of Fang et al. (2008) limited to *O. sativa* sequences. In conclusion, no major inconsistency was observed between the outcomes of phylogeny analyses and expert definition of OGs.

NAC gene structure was found to be largely consistent with OGs based on NAC sequence similarity. A few exceptions to the three-exon structure were observed in groups 1, 2, 3, 6 and 8. The most frequent changes are intron losses (i.e. exon mergings). One and two additional exons were observed at the beginning of the genes of OGs 7a–b and 7c–d, respectively. Genes of groups 4, 5 and 7 underwent exon/intron structure changes in the TRR, which is also very variable in its amino acid sequence. Similarity in gene structure was very common within each OG. However some differences could be observed. These differences could be explained by independent evolution of the genes (gain or loss of introns), but also they could be the results of erroneous annotations. In fact, the annotation of these genes is difficult in the very variable C-terminal region, due to lack of similarity with other model genes. Moreover, the level of expression of these genes is often low or tissue/condition specific (Wang et al. 2013), which reduces the representation of NAC genes in transcriptome databases.

All the sequences in each of the 40 OGs are supposed to derive from an individual ancestral gene that was present in the most recent common ancestor of monocot/dicot species. Most of the group assignations look robust. However, for some more divergent sequences, the assignation remains unreliable and further analysis involving improved annotations or additional species could modify the assignation of these sequences. The observed dicot-specific OGs (3e and 4f) could be a lineage-specific evolution from another group (that could reduce the number of ancestral NAC members) or alternatively, the orthologous genes may have been lost in the monocot lineages. Even if the two dicot-specific OGs do not correspond to an ancestral sequence, at least 38 ancestral NAC sequences pre-dated the divergence of monocot/dicot lineages.

### NAC duplicates

**Fig. 5:**
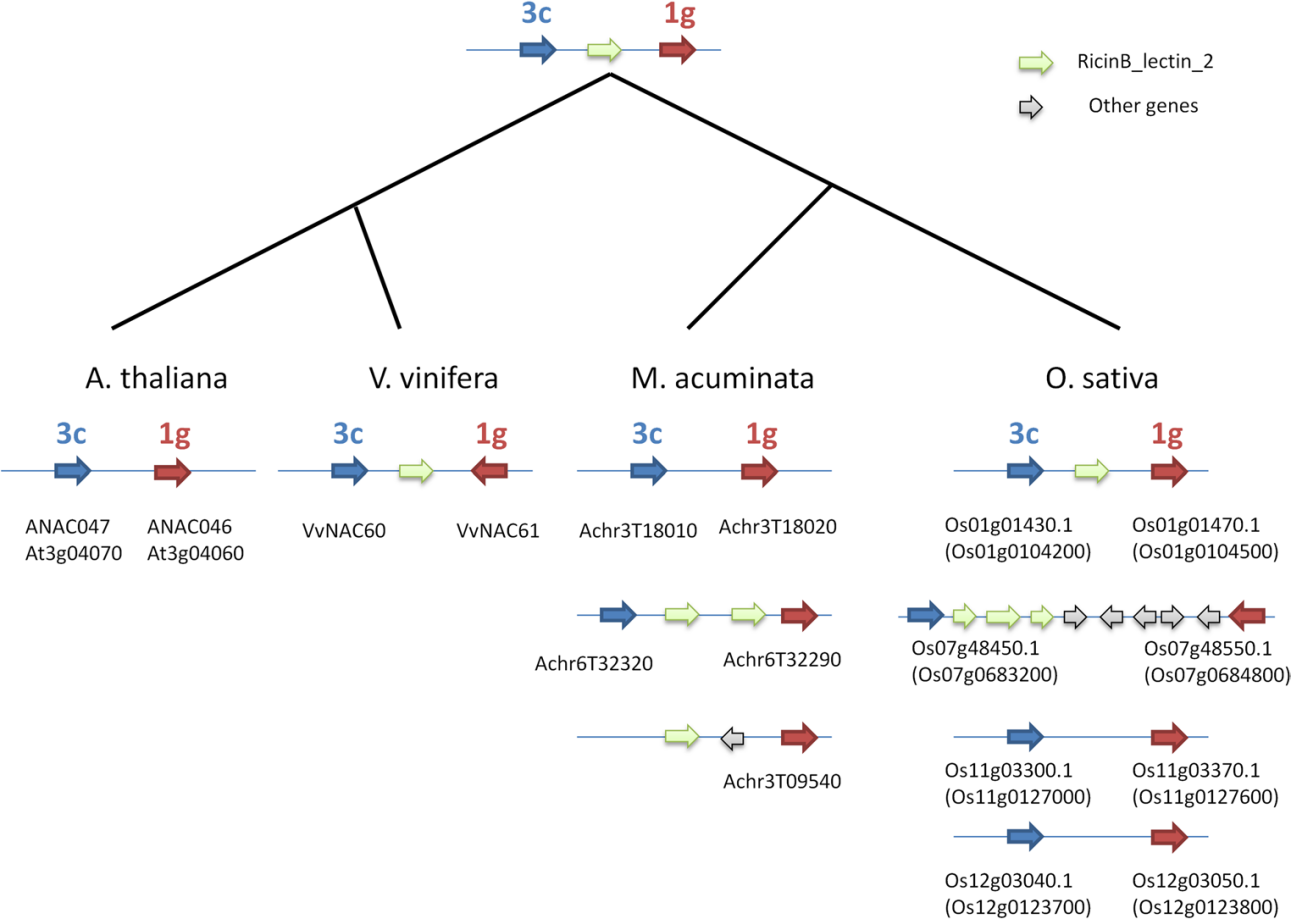
Evolutionary reconstruction of the fate of an ancestral locus having NAC genes of divergent OGs in tandem position. *Blue* and *red* *arrowheads* indicate NAC genes included in OGs 3c and 1g, respectively; *green* *arrowheads* indicate RicinB-lectin_2 genes; *grey arrowheads* indicate other genes

In the OG-specific phylogenies, when several sequences of a given species were included in an OG, in most of the cases these genes appeared clustered together suggesting lineage-specific amplifications. In addition, when the gene location of the sequences in their respective genome was considered, some tandem duplications were observed in *V. vinifera* (VvNAC20-21, 28-32, 51-55) (Wang et al. 2013) and in *A. thaliana* (ANAC003-005, 016-017, 018-019, 046-047, 048-049, 050-053, 055-056, 064-065, 067-69, 073-074, 077-078, 087-088) as well as in *O. sativa* (Nuruzzaman et al. 2010). Since the members of these tandem duplications are often in the same OG, it is likely that these duplications occurred after the lineage separation of the analysed species. Alternatively, some tandem duplications could be more ancient. For example, inside the phylogenetic cluster of the OG 4d, VvNAC20 + ANAC078 and VvNAC21 + ANAC077 are in a separated sub-cluster (aLRT >0.8). It is therefore likely that their tandem duplication took place before the divergence of these two dicot species (Online Resources 13). In *M. acuminata*, none of the detected tandem duplications could be assigned to the same OG, which indicates that their origin pre-dates the divergence between monocots and dicots. It is worth highlighting the tandem distribution of two NAC ancestral sequences (originating OGs 1g and 3c) that was retained during the divergence of monocots and dicots (Fig. 5). These genes are still in tandem in all the four analysed species and they were even multiplied during the *O. sativa* and *M. acuminata* segmental duplications: VvNAC61/ANAC46/Os3g21030/Os7g48550/Os11g03370/Os12g03050/GSMUA_Achr3T01820/GSMUA_Achr6T32290 (OG 1 g) and VvNAC60/ANAC47/Os3g21060/Os7g48450/Os11g03300/Os12g03040GSMUA_Achr3T01810/GSMUA_Achr6T32320 (OG 3c). Between the two NAC members, a RicinB gene is often found (Fig. 5). In addition to the tandem position, when the 5′–3′ orientation is considered, the 3c copies precede the 1 g ones in all the loci except in *V. vinifera* and *O. sativa* chromosome 7, where the 1 g copies have undergone inversion (Fig. 5). Since the genes in OGs 1 g and 3c occupy very remote positions in the phylogenetic tree of all the species, one can speculate that these genes originated through more ancient tandem duplication than the monocot/dicot divergence. Alternatively, it is possible that a fortuitous rearrangement juxtaposed these genes prior to the monocot/dicot divergence. Nuruzzaman et al. (2010) already pinpointed the duplications of NAC containing regions in chr.3/chr.7 and chr.11/chr.12 in *O. sativa*. These regions coincide with known duplications in the evolution of the *O. sativa* genome (Yu et al. 2005). By contrast, the duplication involving the ancestral regions of chr.3/chr.7 and chr.11/chr.12 was never observed. This duplication could have originated from an ancient WGD or via a segmental duplication involving a limited chromosome region. Similarly, duplication in *M. acuminata* was not coincident with the most recent two tetraploidization cycles (D’Hont et al. 2012) and could have originated during the older WGD suggested by D’Hont et al. (2012) or during a segmental duplication independent of any WGD.

Globally, a dramatic difference can be observed in the total number of NAC members among the four analysed taxa. Since all these species underwent to independent WGD events, the observed variability could be explained by different levels of the fractionation process (i.e. the loss of duplicated and redundant genes). If this is the main reason for the copy-number differences, similar ratios between NAC and total gene number are expected. When this ratio is calculated, *M. acuminata* showed the highest ratio (4.7 × 10^−3^), followed by *A. thaliana* (4.0 × 10^−3^), *O. sativa* (3.5 × 10^−3^) and *V. vinifera* (2.7 × 10^−3^). If we take in account that significant numbers of NAC genes of *O. sativa* were excluded from the comparative analysis, it appears that *V. vinifera* has a notably lower percentage of NAC genes than other species. Previous studies showed that, after WGDs, the retention of duplicated genes with regulatory functions such as transcription factors is higher than for other kinds of genes (Blanc and Wolfe 2004; Maere 2005). Consequently, the lower number of WGDs experienced by the *V. vinifera* genome could explain the observed lower genomic representation of NAC genes.

**Fig. 6:**
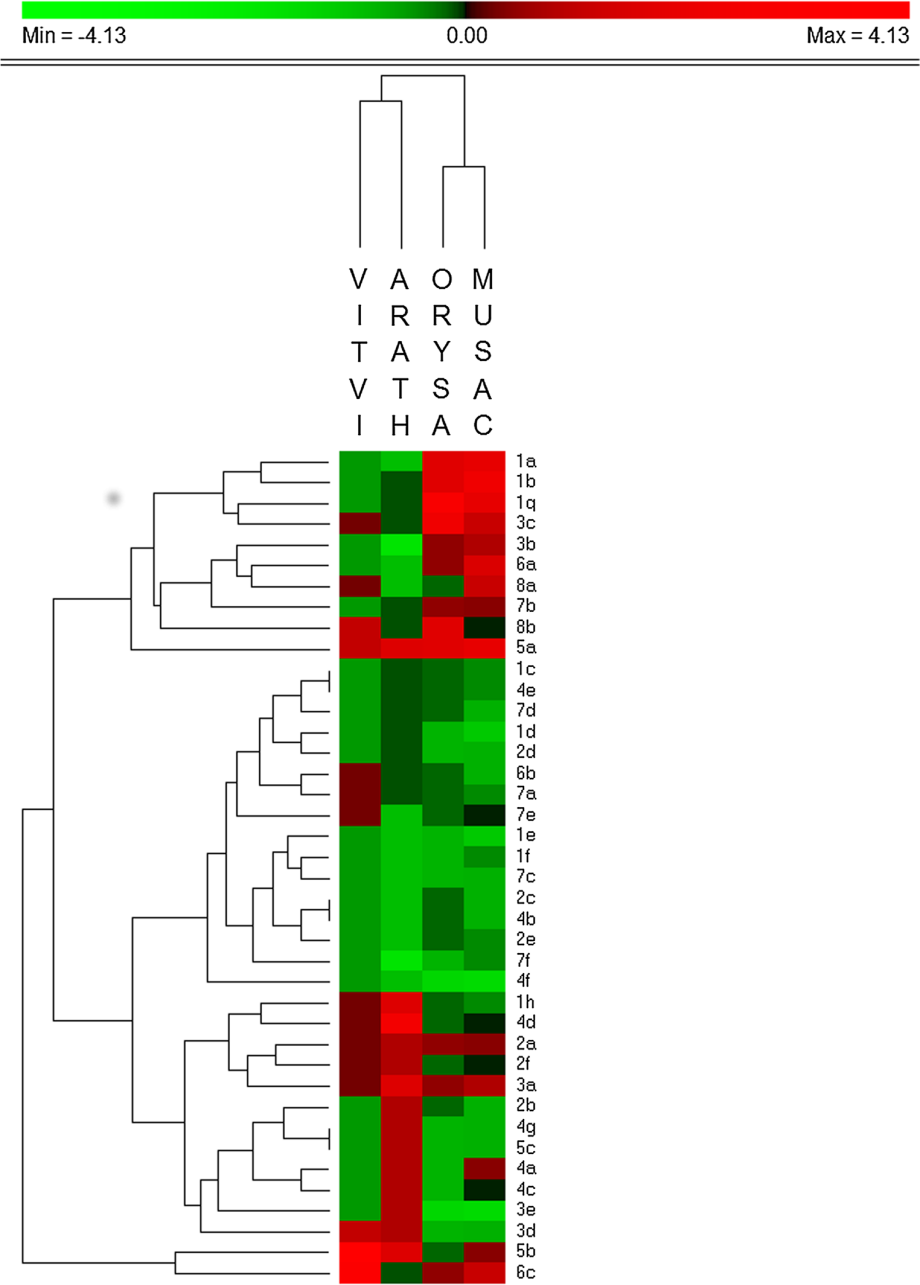
Hierarchical clustering of the 40 NAC OGs analysed in the four species (*V. vinifera*, *A thaliana*, *O. sativa* and *M. acuminata*). The colour gradient from *green* to *red* indicates whether a particular group is significantly smaller or bigger based on Z-score for all genes across the four species. The figure was generated using PermutMatrix (Caraux and Pinloche 2004) with the euclidean distance and the McQuitty clustering parameters

In addition to the comparison of global numbers of NAC copies, the present study allows the analysis of specific OGs. Marked differences in NAC gene number can be observed among OGs, in particular in the *M. acuminata* and *O. sativa* taxa (Fig. 6). These differences suggest that copy retention could vary among different OGs in a same family of transcription factors.

### Potential transfer of functional gene annotation

The NAC OGs are supposed to include most of the NAC copies that have evolved from an ancestral copy existing in the most recent common ancestor of monocots and dicots. It is likely that most of these ancestral genes already had their functions, and that these functions were maintained in the derived species during the independent evolution. The framework of the NAC OGs should provide a useful tool to predict the best candidates for a given function in species for which less information is available. For example, the sequence database provided in Online Resources 1, containing the NACs of four species representative of monocots and dicots, could be used to classify any NAC sequence of a given species by a simple Blastp analysis.

In particular, NACs involved in abiotic stress-resistance or tolerance could be predicted for newly sequenced genomes such as *M. acuminata*. Based on published functions of characterized NAC genes (Table 1), one can suppose that NAC members belonging to the OGs 3, 5 and 6 are mainly involved in response to biotic and abiotic stresses, even if OGs 1g–h appear to also play a role in this domain. Moreover many OGs do not contain any NAC genes characterized for their functions. Consequently, future results should identify an increasing number of potential genes suitable for improving the adaptability of crops to different environments and to climatic changes.

## Conclusion

The NAC gene family plays an important role in the regulation of plant development and stress-resistance/tolerance. A better understanding of the complex ancestral gene history may lead to better functional characterization of these genes. The recent sequencing of the *M. acuminata* genome has helped to elucidate the evolution of the NAC TFs in angiosperms.

Our approach to the study of NAC sequences was based on similarity analysis as inferred by a thorough and systematic examination of reciprocal Blastp results (here called expert orthologous grouping) and classical phylogenetic analysis. Phylogenetic analysis with all NAC sequences provided a global view of the reciprocal relations among NACs, but due to very divergent sequences, the resolution was insufficient to resolve some OGs. We have shown that when limiting the analysis to sequences belonging to more restricted groups, the phylogenetic resolution increases with the increase in available informative positions. Globally, phylogenetic analysis confirmed around two-thirds of the OGs based on similarity, but did not invalidate the remaining third of unresolved OGs. Combined with Blastp analysis, we were able to perform a more effective comparison between each pair of sequences with regard to the filtered multiple alignment that may not include divergent but informative sequence regions to resolve unclear OGs.

The OGs resulting from our analysis should provide a reference framework useful for functional gene annotation transfer in the NAC transcription factor family. These orthologous groups provide a curated resource for large-scale protein sequence annotation of NAC transcription factors. The established orthology relationships also provide a useful reference for NAC function studies in newly sequenced genomes such as *M. acuminata* and other plant species.

## Electronic supplementary material

Below is the link to the electronic supplementary material.

Online Resource 1 Fasta file containing the 407 translated sequences of NAC genes of *M.acuminata*, *O. sativa*, *V. vinifera* and *A. thaliana* analysed in this study (FA 157 kb)

Online Resource 2 List of sequence accessions used in this study and relative concise description of eventual modifications to the already published sequences (XLS 112 kb)

Online Resource 3 List of duplicated genes assigned to segmental duplication in M. acuminate genome, as detected by CoGe SynMap analysis (XLS 3111 kb)

Online Resource 4 Species tree used to reconciliate gene trees with RAPGreen (Dufayard et al. 2005) (XML 3 kb)

Online Resource 5 OrthoMCL clusters obtained with different inflation parameters (1.5, 5, 8, 10, 12, and 14, respectively) (TXT 11 kb)

Online Resource 6 OrthoMCL clusters obtained with different inflation parameters (1.5, 5, 8, 10, 12, and 14, respectively) (TXT 11 kb)

Online Resource 7 OrthoMCL clusters obtained with different inflation parameters (1.5, 5, 8, 10, 12, and 14, respectively) (TXT 11 kb)

Online Resource 8 OrthoMCL clusters obtained with different inflation parameters (1.5, 5, 8, 10, 12, and 14, respectively) (TXT 11 kb)

Online Resource 9 OrthoMCL clusters obtained with different inflation parameters (1.5, 5, 8, 10, 12, and 14, respectively) (TXT 11 kb)

Online Resource 10 OrthoMCL clusters obtained with different inflation parameters (1.5, 5, 8, 10, 12, and 14, respectively) (TXT 11 kb)

Online Resource 11 Phylogenetic tree (Newick format) obtained with all the sequences included in the inferred OGs (NWK 21 kb)

Online Resource 12 Phylogenetic trees obtained with sequences of OGs 2a-f and OGs 7a-d (PDF 347 kb)

Online Resource 13 Phylogenetic trees obtained with sequences included in 36 inferred OGs (PDF 2528 kb)
