# Supplementary figures and images for: Genomic analysis of NAC transcription factors in banana (Musa acuminata) and definition of NAC orthologous groups for monocots and dicots
Source: Plant Mol Biol. 2014 Feb 26;85(1):63–80. doi: 10.1007/s11103-013-0169-2 (PMC4151281; doi:10.1007/s11103-013-0169-2)

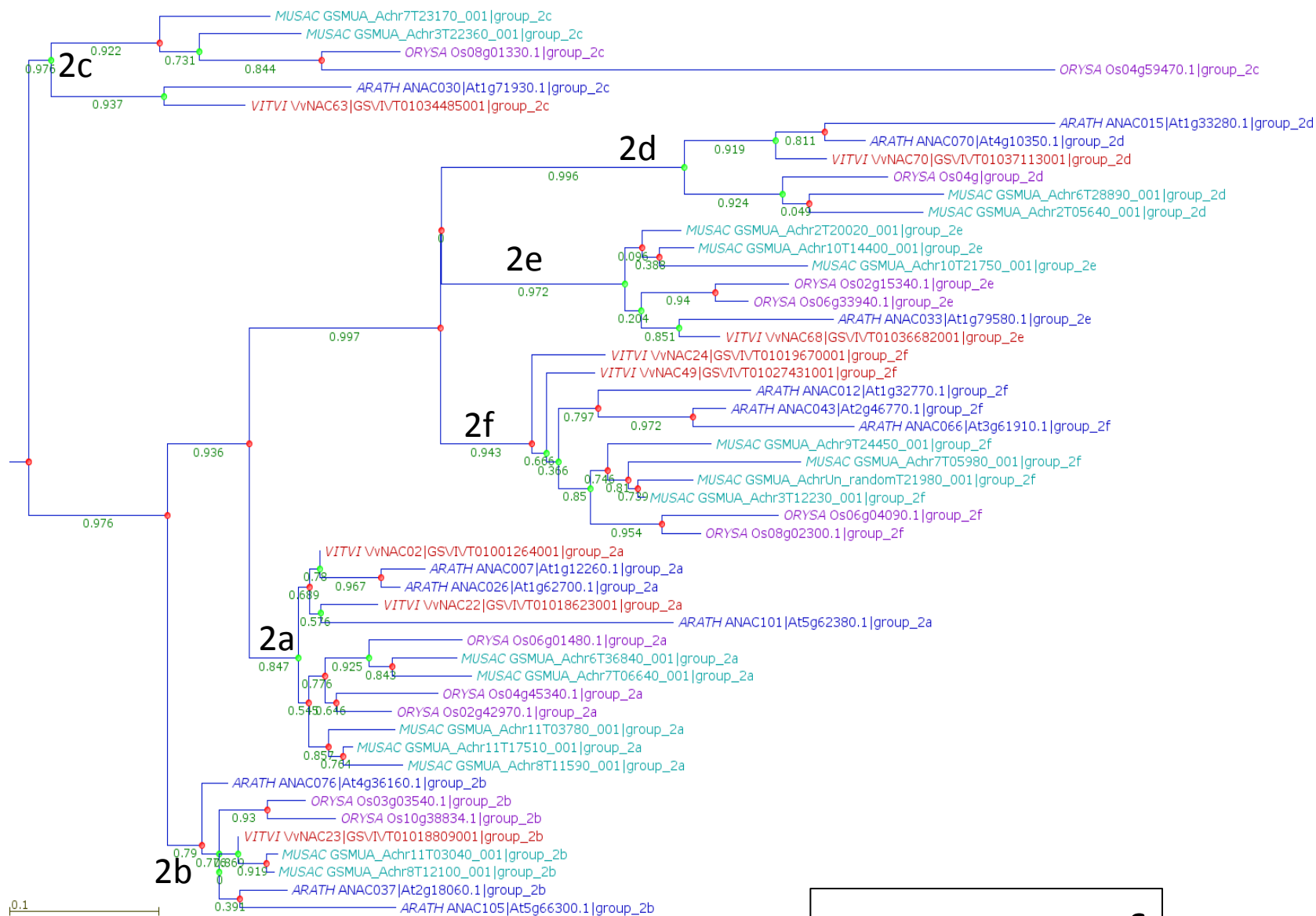

Groups 2a-f

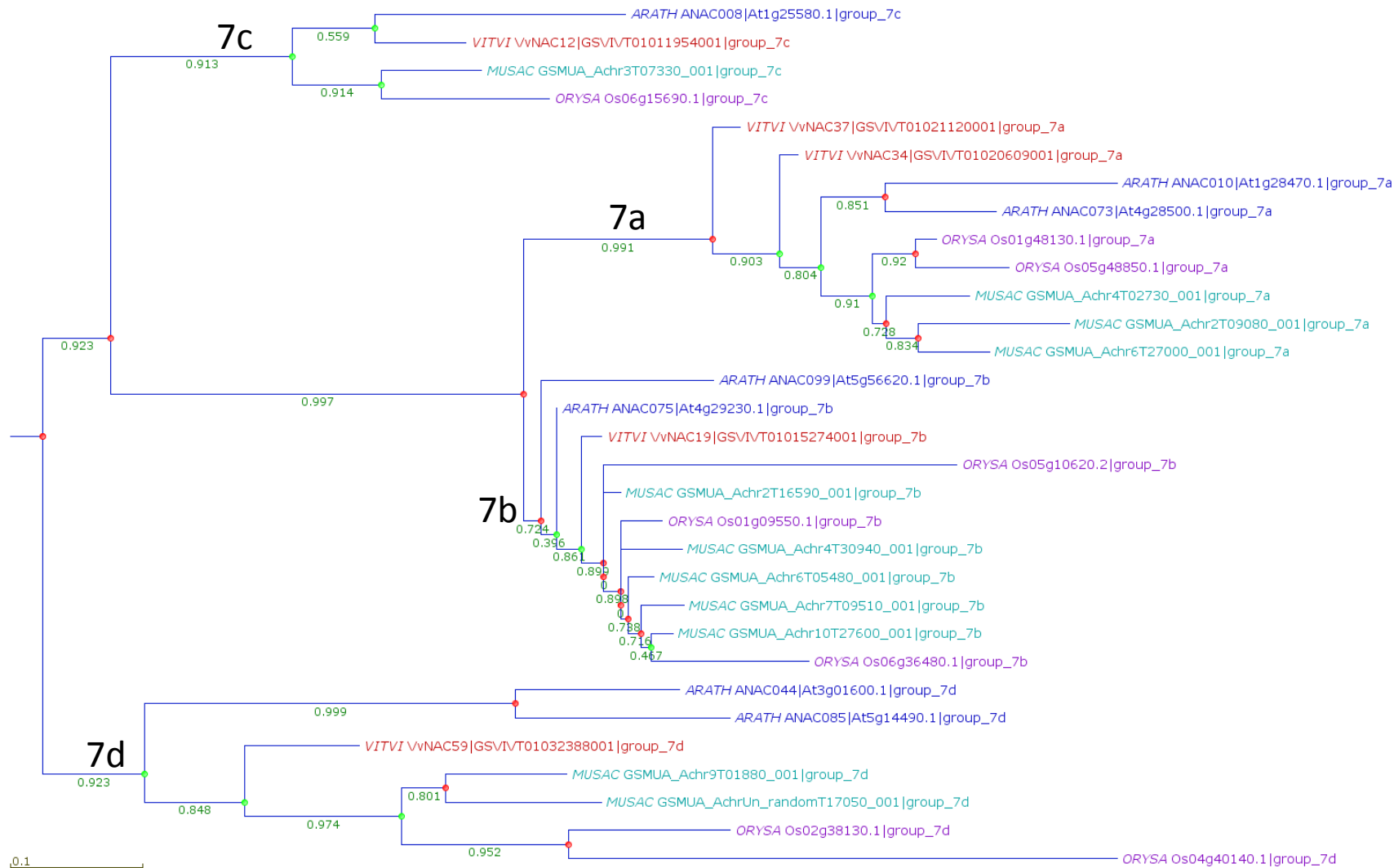

Groups 7a-d

Supplement: Supplementary file 12 — Phylogenetic trees obtained with sequences of OGs 2a-f and OGs 7a-d (PDF 347 kb) [file 11103_2013_169_MOESM12_ESM.pdf]
