# Supplementary material for: Genomic analysis of NAC transcription factors in banana (Musa acuminata) and definition of NAC orthologous groups for monocots and dicots
Source: Plant Mol Biol. 2014 Feb 26;85(1):63–80. doi: 10.1007/s11103-013-0169-2 (PMC4151281; doi:10.1007/s11103-013-0169-2)
Supplement: Supplementary file 13 — Phylogenetic trees obtained with sequences included in 36 inferred OGs (PDF 2528 kb) [file 11103_2013_169_MOESM13_ESM.pdf]

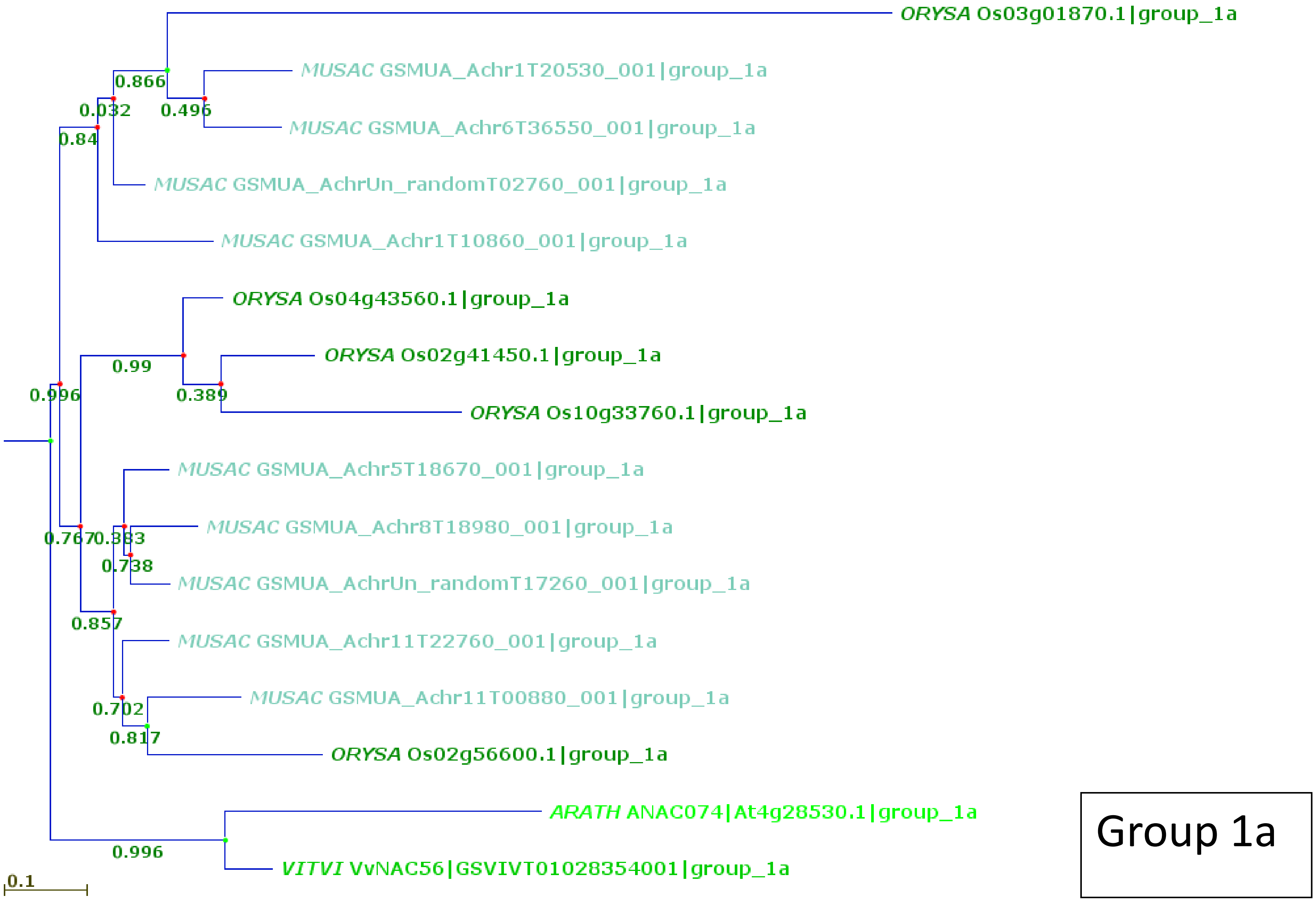
ORYSA Os03g01870.1|group_1a
MUSAC GSMUA_Achr1T20530_001|group_1a
MUSAC GSMUA_Achr6T36550_001|group_1a
MUSAC GSMUA_AchrUn_randomT02760_001|group_1a
MUSAC GSMUA_Achr1T10860_001|group_1a
ORYSA Os04g43560.1|group_1a
ORYSA Os02g41450.1|group_1a
ORYSA Os10g33760.1|group_1a
MUSAC GSMUA_Achr5T18670_001|group_1a
MUSAC GSMUA_Achr8T18980_001|group_1a
MUSAC GSMUA_AchrUn_randomT17260_001|group_1a
MUSAC GSMUA_Achr11T22760_001|group_1a
MUSAC GSMUA_Achr11T00880_001|group_1a
ORYSA Os02g56600.1|group_1a
ARATH ANAC074|At4g28530.1|group_1a
VITVI VvNAC56|GSVIVT01028354001|group_1a
0.866
0.032
0.496
0.84
0.99
0.996
0.389
0.767
0.383
0.738
0.857
0.702
0.817
0.996
0.1
Group 1a

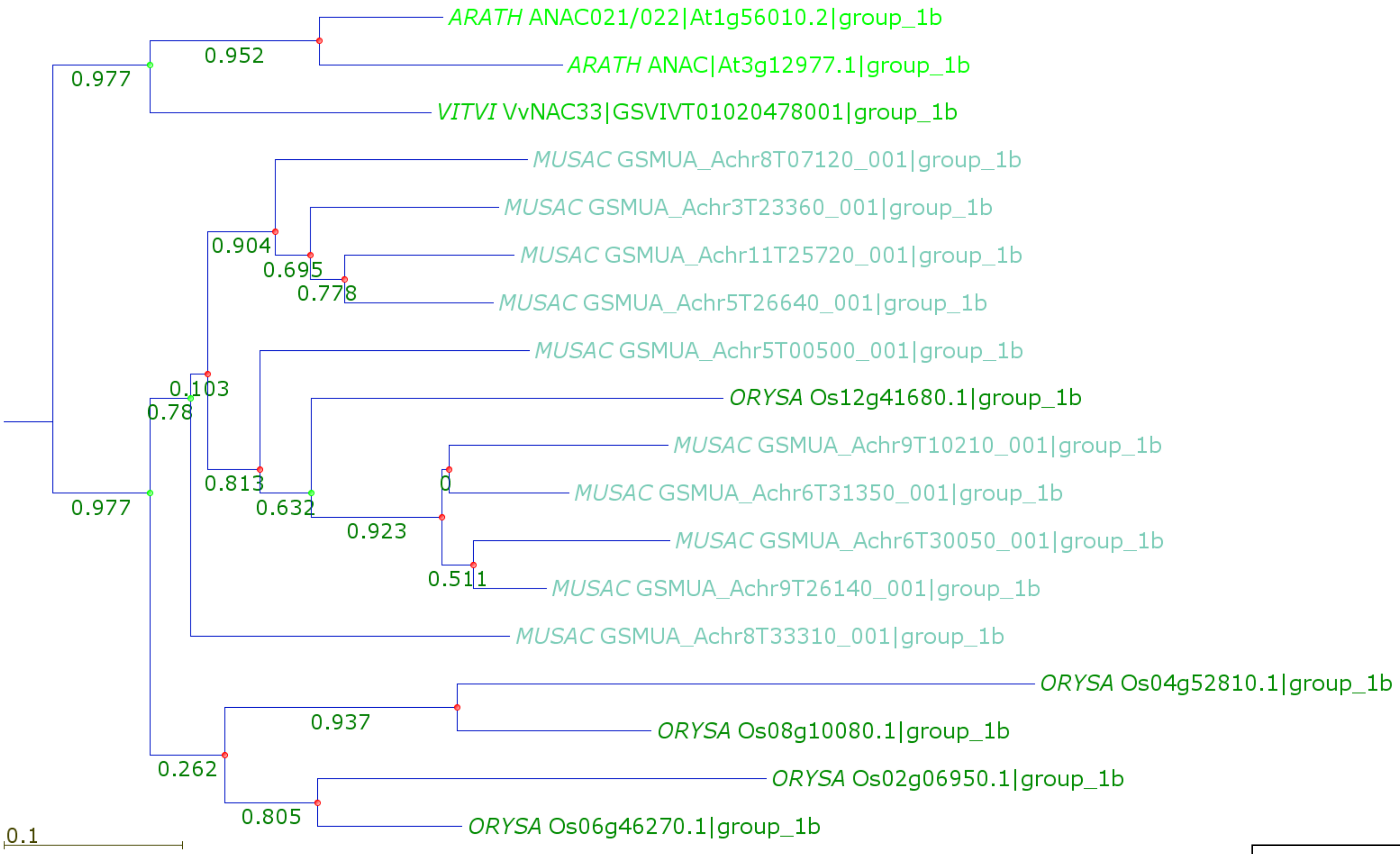

ARATH ANAC021/022|At1g56010.2|group_1b
ARATH ANAC|At3g12977.1|group_1b
VITVI VvNAC33|GSVIVT01020478001|group_1b
MUSAC GSMUA_Achr8T07120_001|group_1b
MUSAC GSMUA_Achr3T23360_001|group_1b
MUSAC GSMUA_Achr11T25720_001|group_1b
MUSAC GSMUA_Achr5T26640_001|group_1b
MUSAC GSMUA_Achr5T00500_001|group_1b
ORYSA Os12g41680.1|group_1b
MUSAC GSMUA_Achr9T10210_001|group_1b
MUSAC GSMUA_Achr6T31350_001|group_1b
MUSAC GSMUA_Achr6T30050_001|group_1b
MUSAC GSMUA_Achr9T26140_001|group_1b
MUSAC GSMUA_Achr8T33310_001|group_1b
ORYSA Os04g52810.1|group_1b
ORYSA Os08g10080.1|group_1b
ORYSA Os02g06950.1|group_1b
ORYSA Os06g46270.1|group_1b
0.952
0.977
0.904
0.695
0.778
0.103
0.78
0.813
0.632
0
0.923
0.511
0.977
0.937
0.262
0.805
0.1

Group 1b

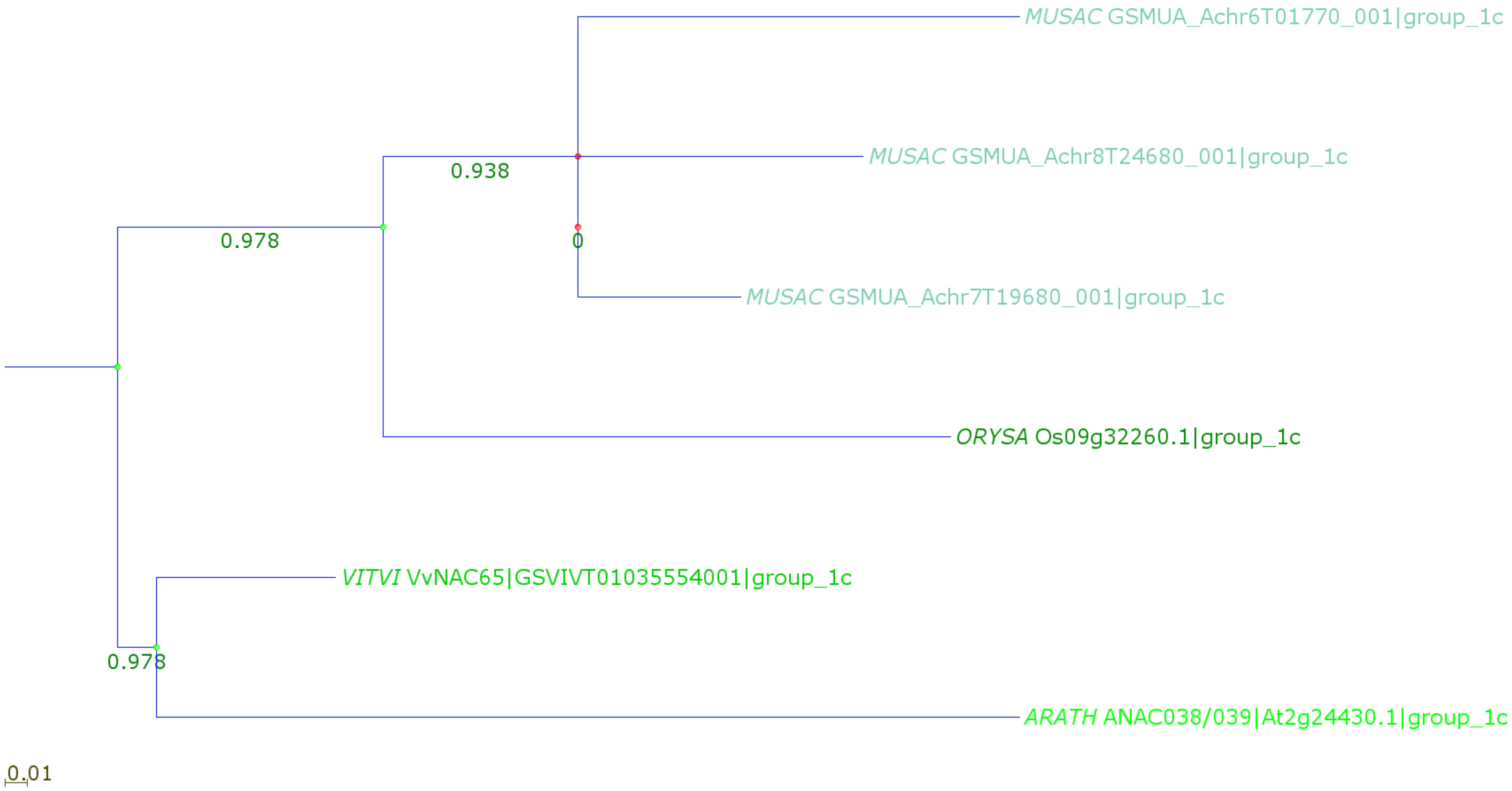
MUSAC GSMUA_Achr6T01770_001|group_1c
MUSAC GSMUA_Achr8T24680_001|group_1c
0.938
0.978
0
MUSAC GSMUA_Achr7T19680_001|group_1c
ORYSA Os09g32260.1|group_1c
VITVI VvNAC65|GSVIVT01035554001|group_1c
0.978
ARATH ANAC038/039|At2g24430.1|group_1c
0.01

Group 1c

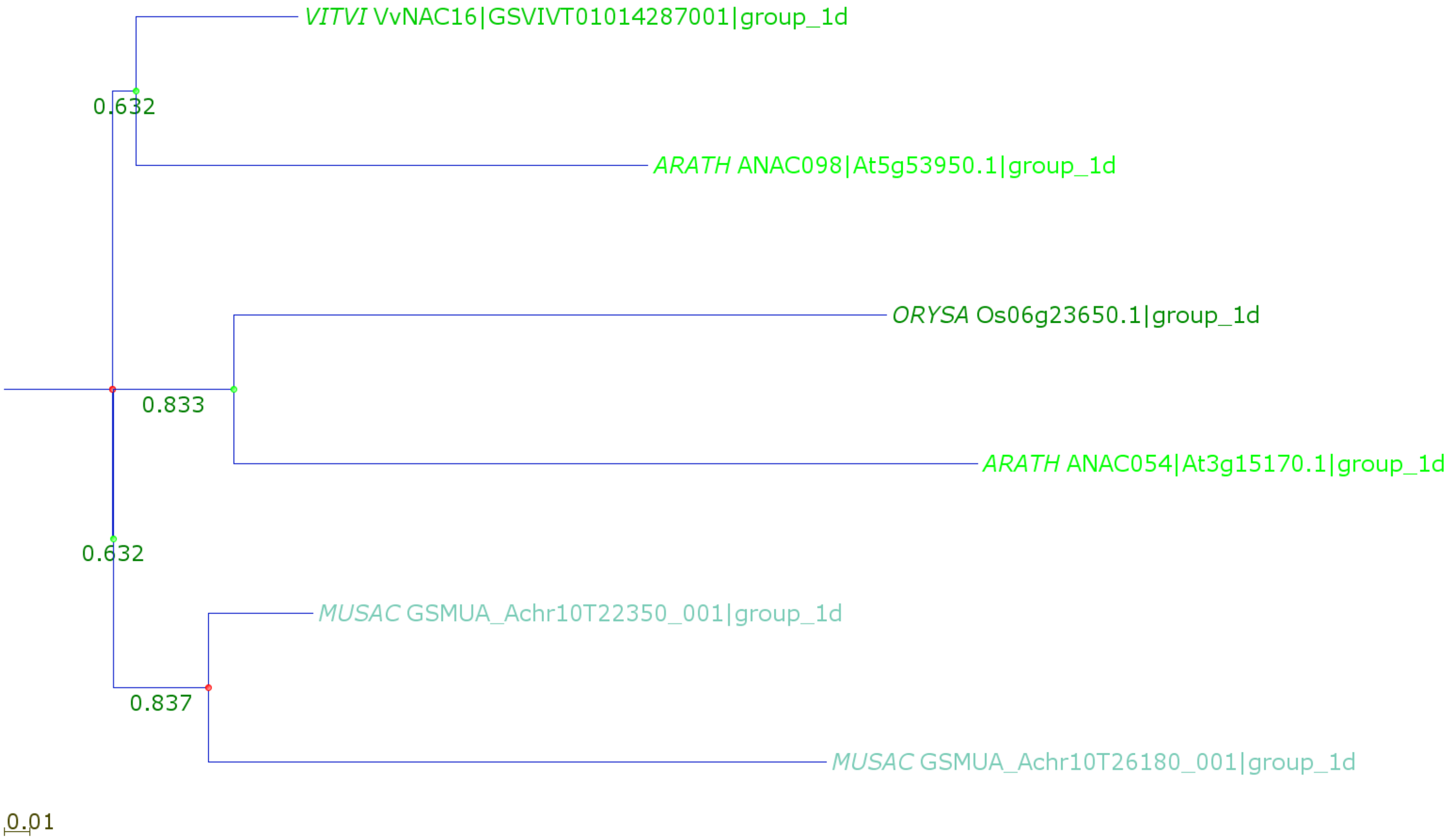
VITVI VvNAC16|GSVIVT01014287001|group_1d
0.632
ARATH ANAC098|At5g53950.1|group_1d
ORYSA Os06g23650.1|group_1d
0.833
ARATH ANAC054|At3g15170.1|group_1d
0.632
MUSAC GSMUA_Achr10T22350_001|group_1d
0.837
MUSAC GSMUA_Achr10T26180_001|group_1d
0.01

Group 1d

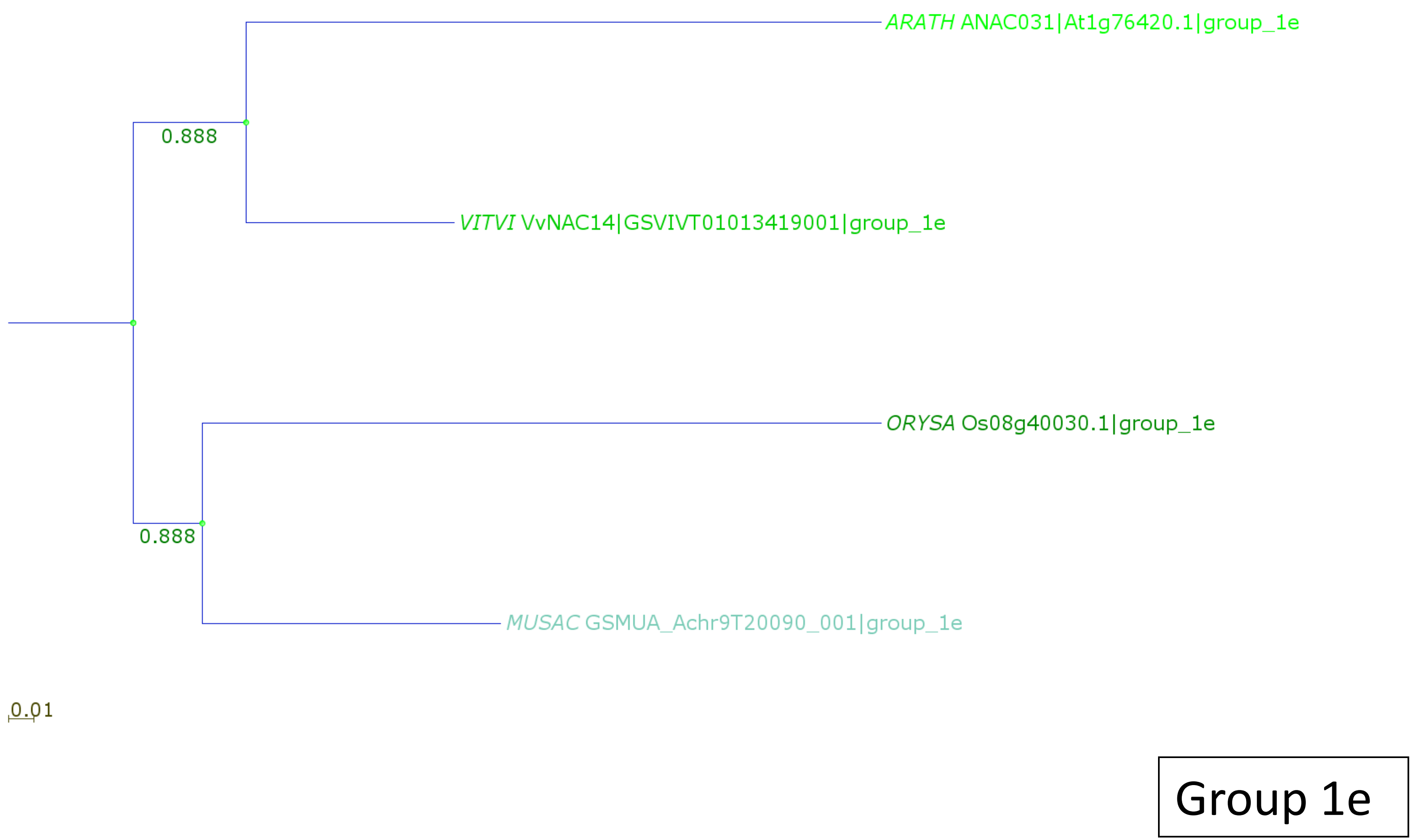
ARATH ANAC031|At1g76420.1|group_1e
0.888
VITVI VvNAC14|GSVIVT01013419001|group_1e
ORYSA Os08g40030.1|group_1e
0.888
MUSAC GSMUA_Achr9T20090_001|group_1e
0.01
Group 1e

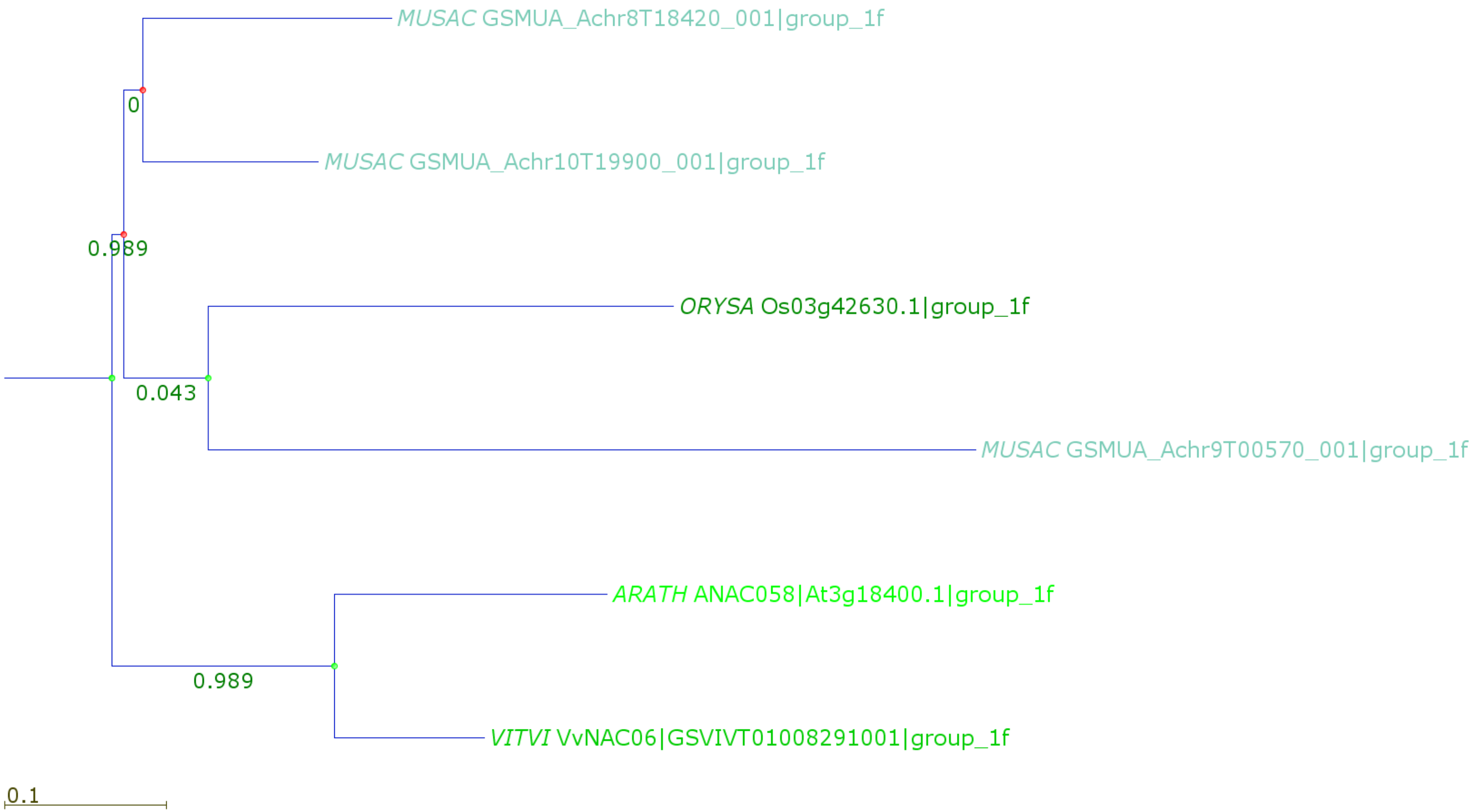
MUSAC GSMUA_Achr8T18420_001|group_1f
0
MUSAC GSMUA_Achr10T19900_001|group_1f
0.989
ORYSA Os03g42630.1|group_1f
0.043
MUSAC GSMUA_Achr9T00570_001|group_1f
ARATH ANAC058|At3g18400.1|group_1f
0.989
VITVI VvNAC06|GSVIVT01008291001|group_1f
0.1

Group 1f

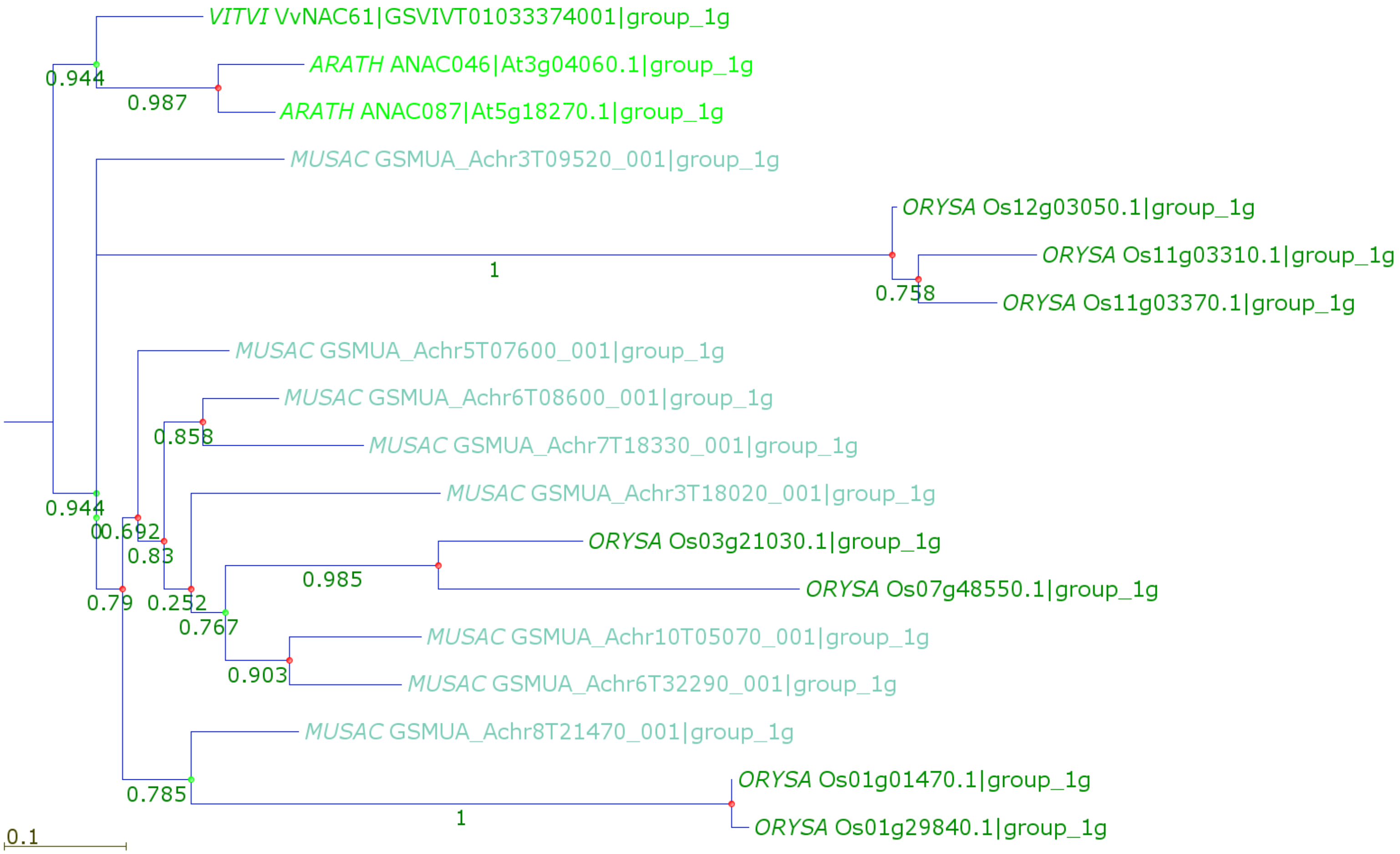

VITVI VvNAC61|GSVIVT01033374001|group_1g
ARATH ANAC046|At3g04060.1|group_1g
ARATH ANAC087|At5g18270.1|group_1g
MUSAC GSMUA_Achr3T09520_001|group_1g
ORYSA Os12g03050.1|group_1g
ORYSA Os11g03310.1|group_1g
ORYSA Os11g03370.1|group_1g
MUSAC GSMUA_Achr5T07600_001|group_1g
MUSAC GSMUA_Achr6T08600_001|group_1g
MUSAC GSMUA_Achr7T18330_001|group_1g
MUSAC GSMUA_Achr3T18020_001|group_1g
ORYSA Os03g21030.1|group_1g
ORYSA Os07g48550.1|group_1g
MUSAC GSMUA_Achr10T05070_001|group_1g
MUSAC GSMUA_Achr6T32290_001|group_1g
MUSAC GSMUA_Achr8T21470_001|group_1g
ORYSA Os01g01470.1|group_1g
ORYSA Os01g29840.1|group_1g
0.944
0.987
1
0.758
0.858
0.944
0.692
0.83
0.79
0.252
0.767
0.985
0.903
0.785
1
0.1

Group 1g

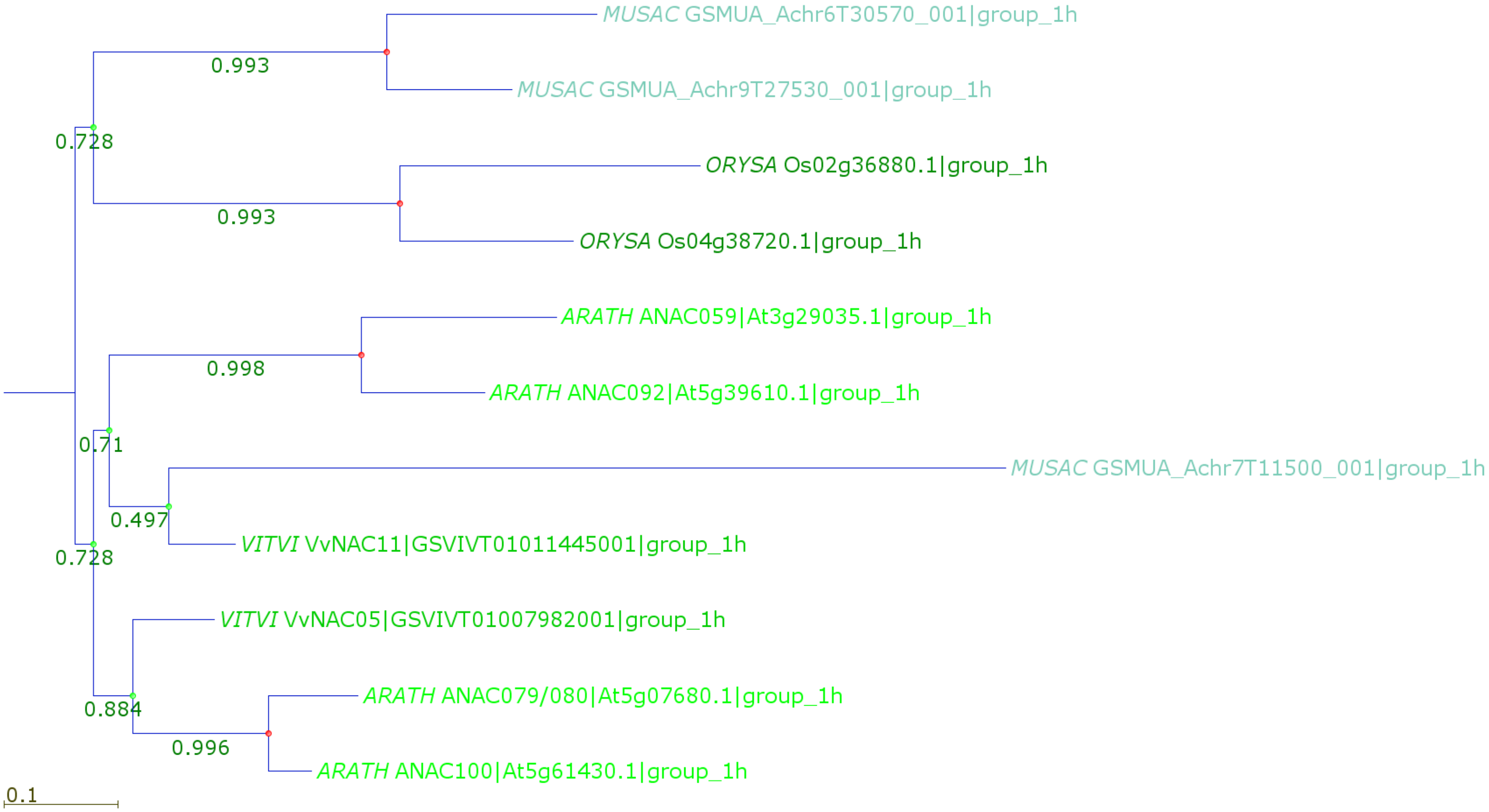
MUSAC GSMUA_Achr6T30570_001|group_1h
MUSAC GSMUA_Achr9T27530_001|group_1h
ORYSA Os02g36880.1|group_1h
ORYSA Os04g38720.1|group_1h
ARATH ANAC059|At3g29035.1|group_1h
ARATH ANAC092|At5g39610.1|group_1h
MUSAC GSMUA_Achr7T11500_001|group_1h
VITVI VvNAC11|GSVIVT01011445001|group_1h
VITVI VvNAC05|GSVIVT01007982001|group_1h
ARATH ANAC079/080|At5g07680.1|group_1h
ARATH ANAC100|At5g61430.1|group_1h
0.993
0.993
0.728
0.998
0.71
0.497
0.728
0.884
0.996
0.1

Group 1h

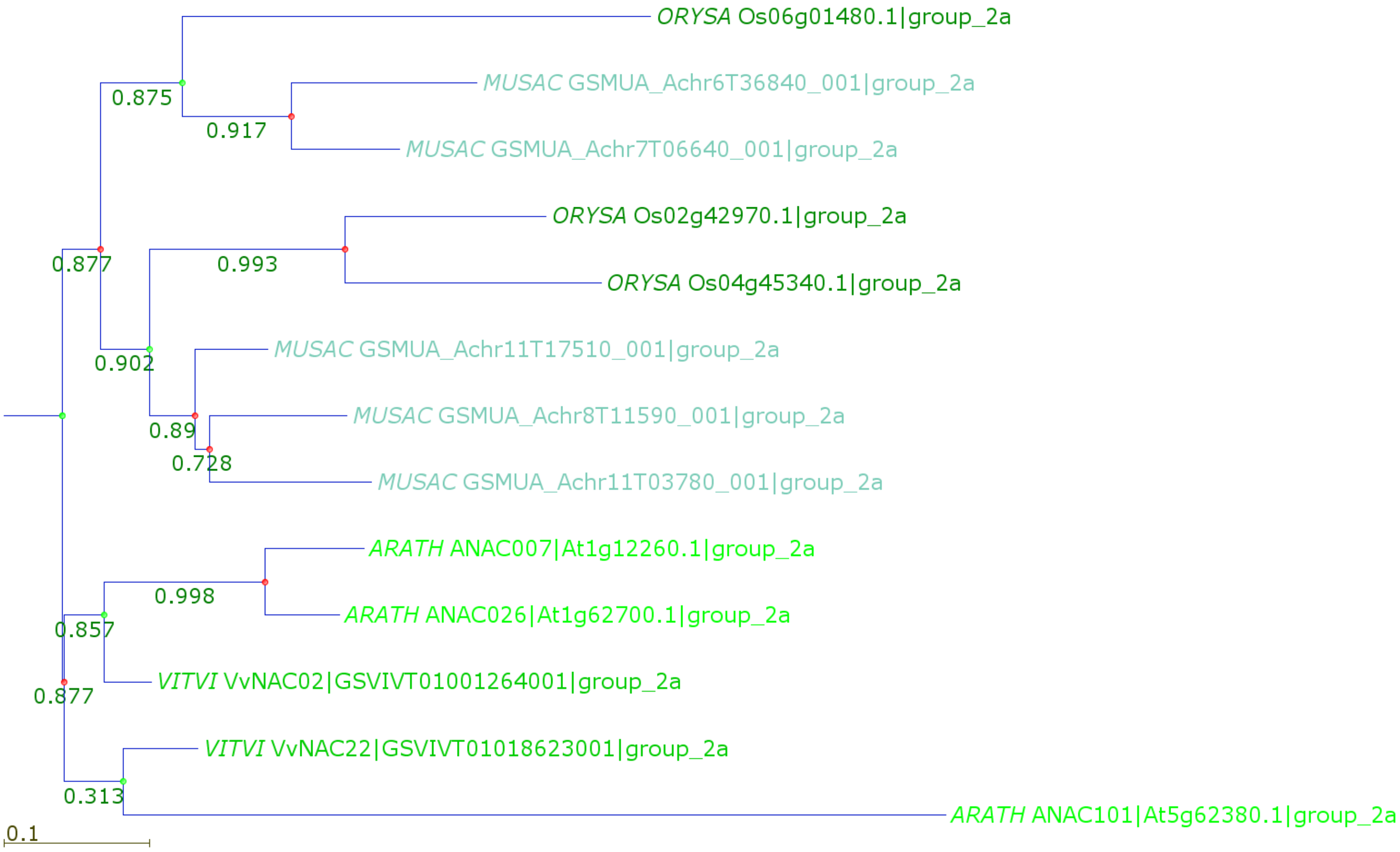
ORYSA Os06g01480.1|group_2a
MUSAC GSMUA_Achr6T36840_001|group_2a
MUSAC GSMUA_Achr7T06640_001|group_2a
ORYSA Os02g42970.1|group_2a
ORYSA Os04g45340.1|group_2a
MUSAC GSMUA_Achr11T17510_001|group_2a
MUSAC GSMUA_Achr8T11590_001|group_2a
MUSAC GSMUA_Achr11T03780_001|group_2a
ARATH ANAC007|At1g12260.1|group_2a
ARATH ANAC026|At1g62700.1|group_2a
VITVI VvNAC02|GSVIVT01001264001|group_2a
VITVI VvNAC22|GSVIVT01018623001|group_2a
ARATH ANAC101|At5g62380.1|group_2a
0.875
0.917
0.877
0.993
0.902
0.89
0.728
0.998
0.857
0.877
0.313
0.1

Group 2a

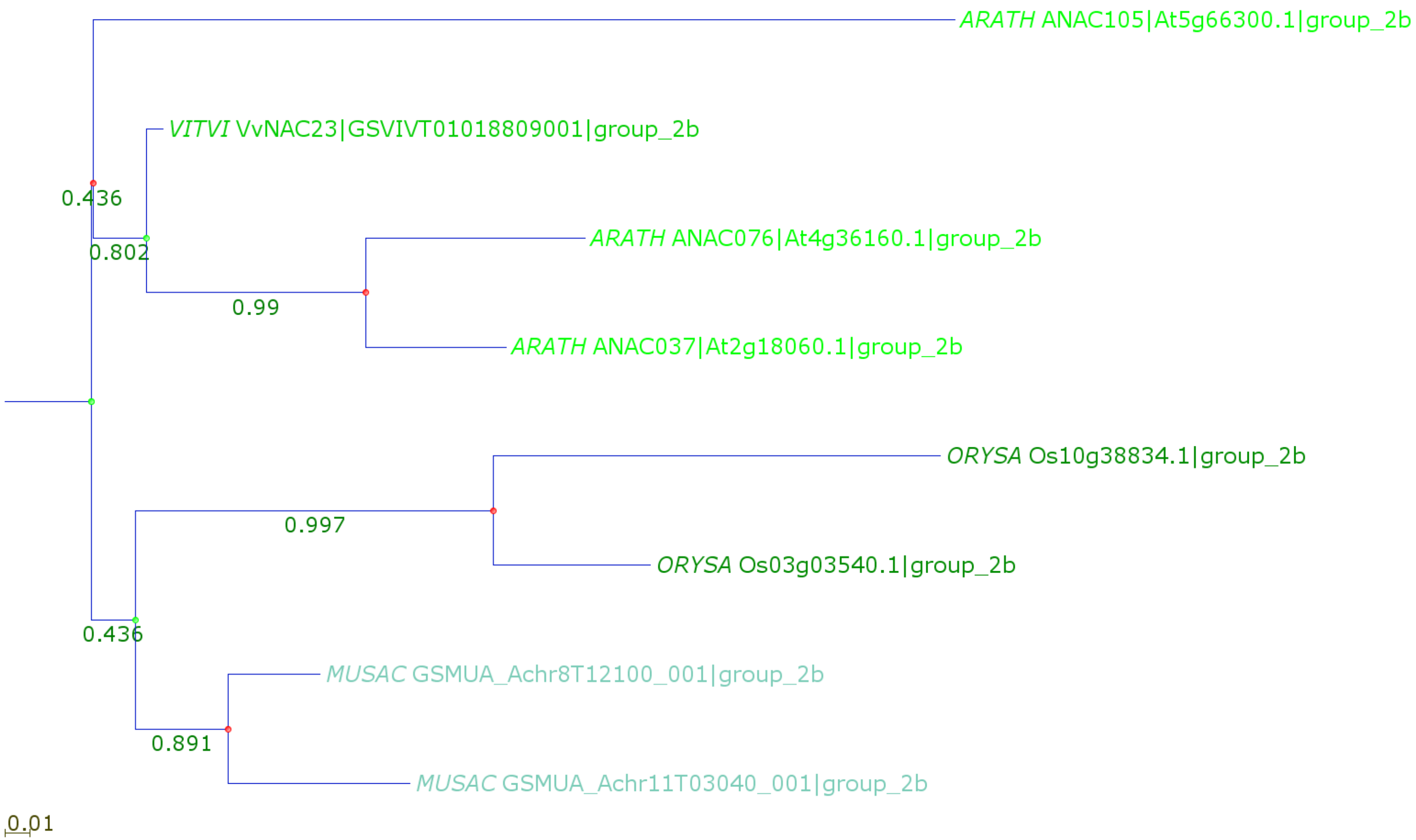
ARATH ANAC105|At5g66300.1|group_2b
VITVI VvNAC23|GSVIVT01018809001|group_2b
0.436
0.802
ARATH ANAC076|At4g36160.1|group_2b
0.99
ARATH ANAC037|At2g18060.1|group_2b
ORYSA Os10g38834.1|group_2b
0.997
ORYSA Os03g03540.1|group_2b
0.436
MUSAC GSMUA_Achr8T12100_001|group_2b
0.891
MUSAC GSMUA_Achr11T03040_001|group_2b
0.01

Group 2b

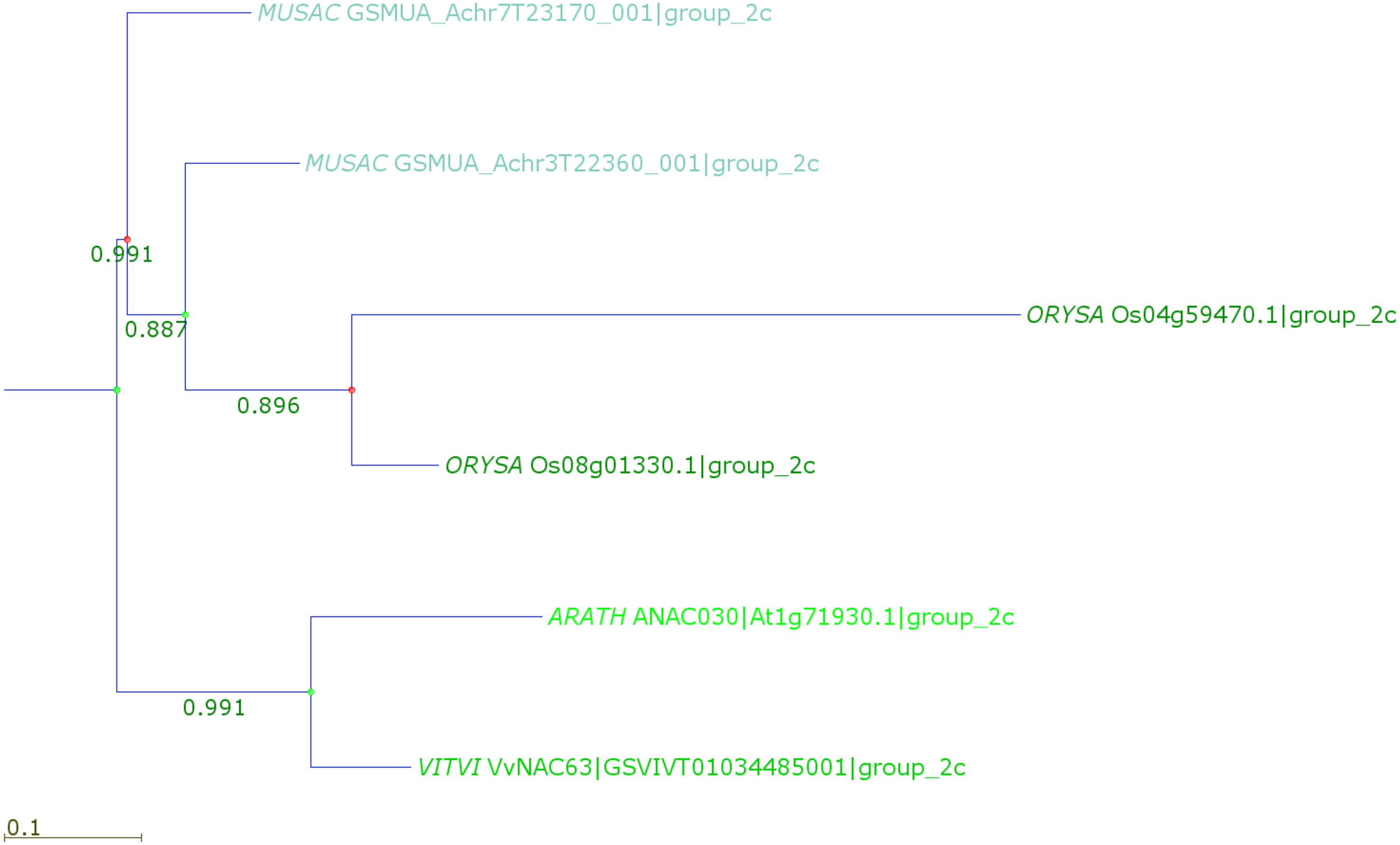
MUSAC GSMUA_Achr7T23170_001|group_2c
MUSAC GSMUA_Achr3T22360_001|group_2c
0.991
0.887
ORYSA Os04g59470.1|group_2c
0.896
ORYSA Os08g01330.1|group_2c
ARATH ANAC030|At1g71930.1|group_2c
0.991
VITVI VvNAC63|GSVIVT01034485001|group_2c
0.1

Group 2c

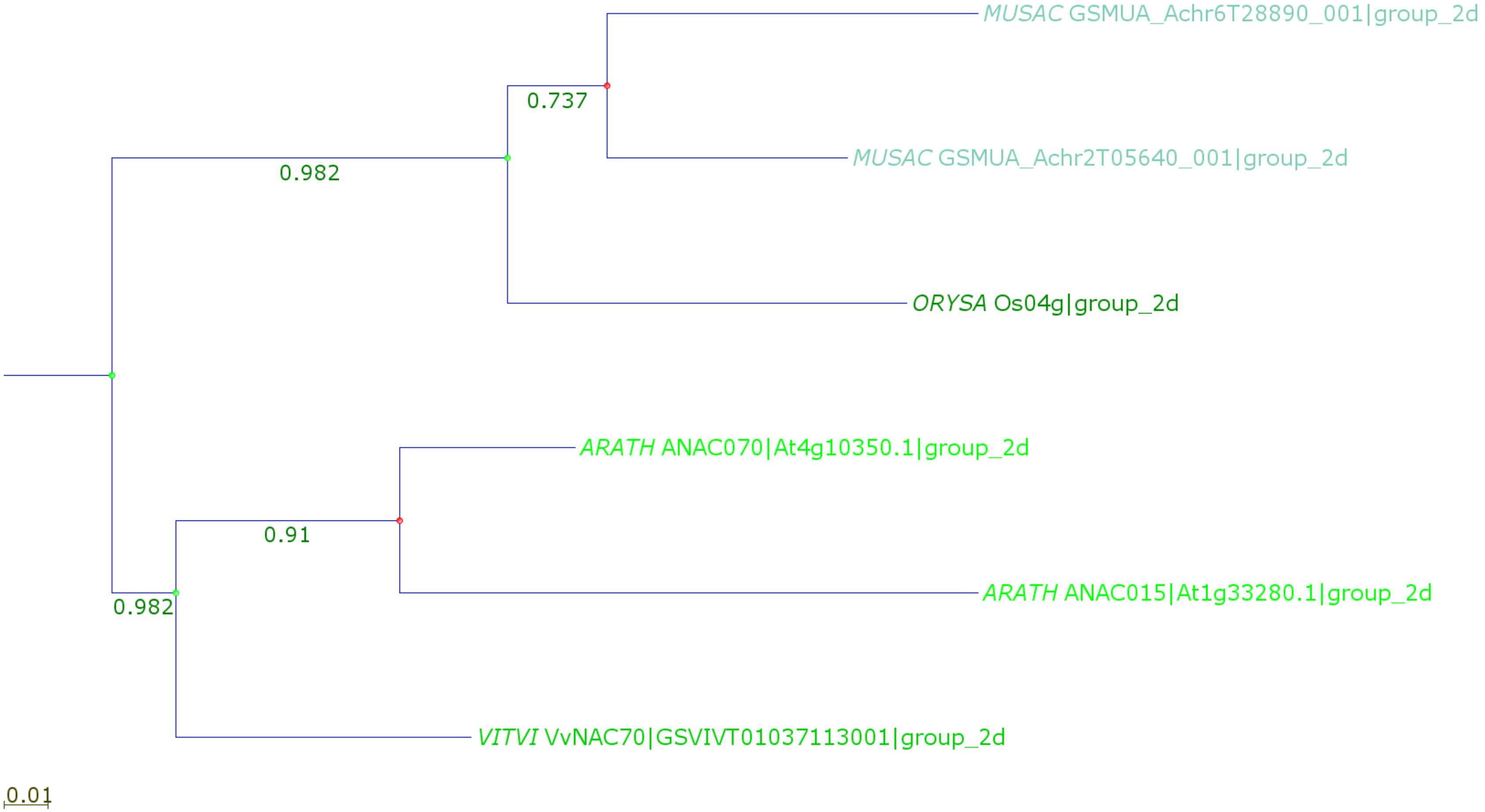
MUSAC GSMUA_Achr6T28890_001|group_2d
0.737
0.982
MUSAC GSMUA_Achr2T05640_001|group_2d
ORYSA Os04g|group_2d
ARATH ANAC070|At4g10350.1|group_2d
0.91
0.982
ARATH ANAC015|At1g33280.1|group_2d
VITVI VvNAC70|GSVIVT01037113001|group_2d
0.01

Group 2d

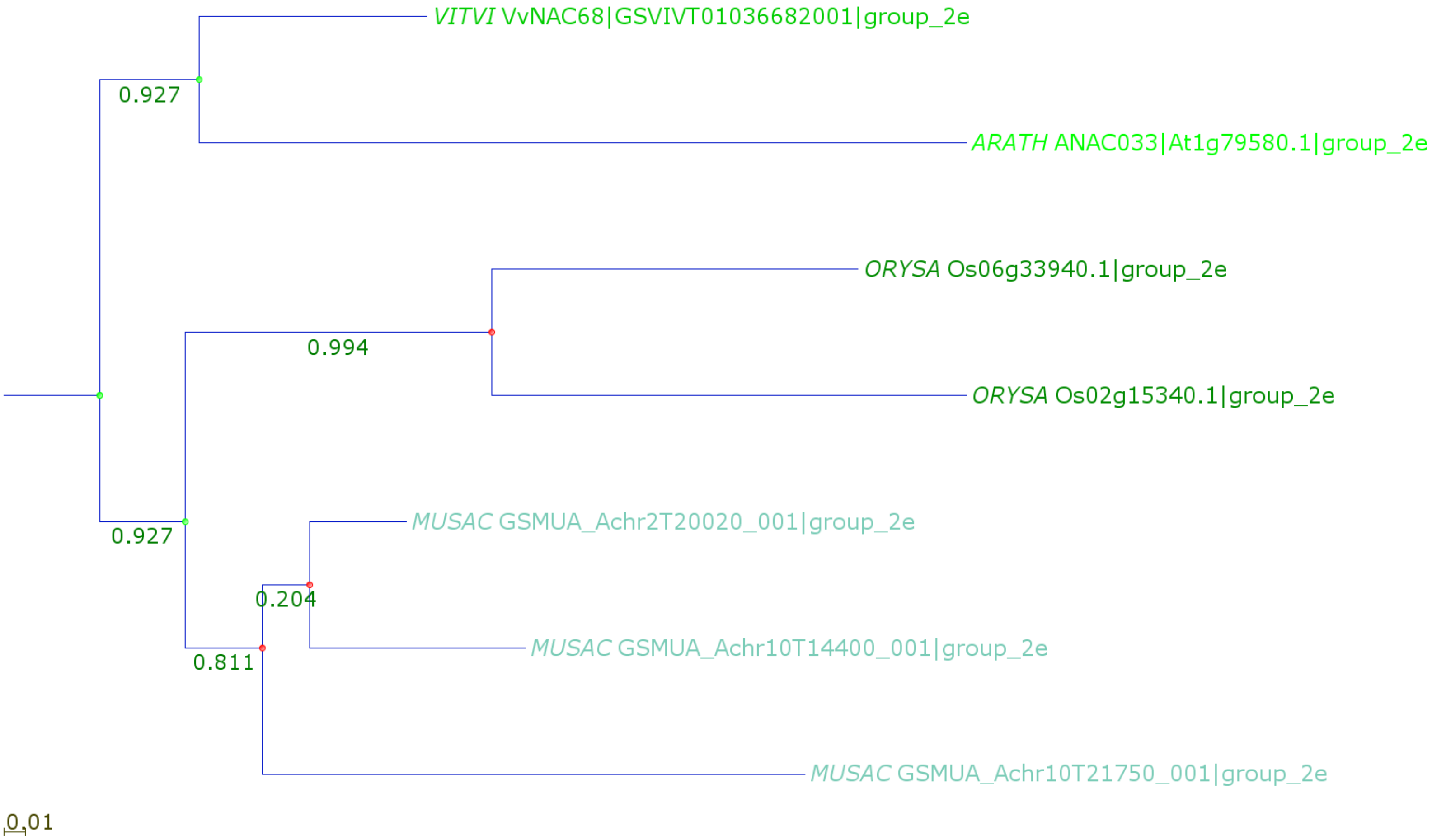

VITVI VvNAC68|GSVIVT01036682001|group_2e
ARATH ANAC033|At1g79580.1|group_2e
ORYSA Os06g33940.1|group_2e
ORYSA Os02g15340.1|group_2e
MUSAC GSMUA_Achr2T20020_001|group_2e
MUSAC GSMUA_Achr10T14400_001|group_2e
MUSAC GSMUA_Achr10T21750_001|group_2e
0.927
0.927
0.994
0.204
0.811
0.01

Group 2e

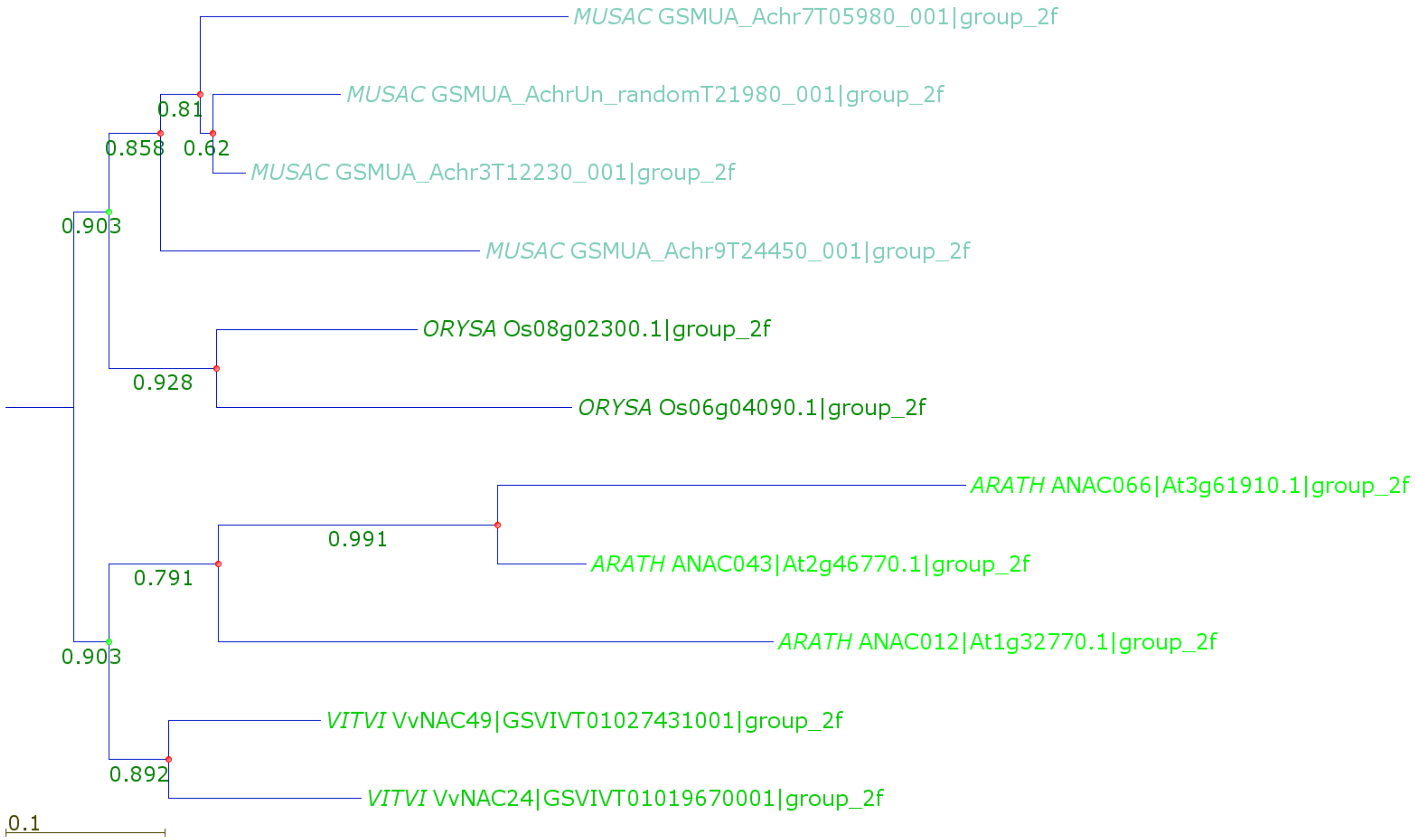

MUSAC GSMUA_Achr7T05980_001|group_2f
MUSAC GSMUA_AchrUn_randomT21980_001|group_2f
MUSAC GSMUA_Achr3T12230_001|group_2f
MUSAC GSMUA_Achr9T24450_001|group_2f
ORYSA Os08g02300.1|group_2f
ORYSA Os06g04090.1|group_2f
ARATH ANAC066|At3g61910.1|group_2f
ARATH ANAC043|At2g46770.1|group_2f
ARATH ANAC012|At1g32770.1|group_2f
VITVI VvNAC49|GSVIVT01027431001|group_2f
VITVI VvNAC24|GSVIVT01019670001|group_2f
0.81
0.858
0.62
0.903
0.928
0.991
0.791
0.903
0.892
0.1

Group 2f

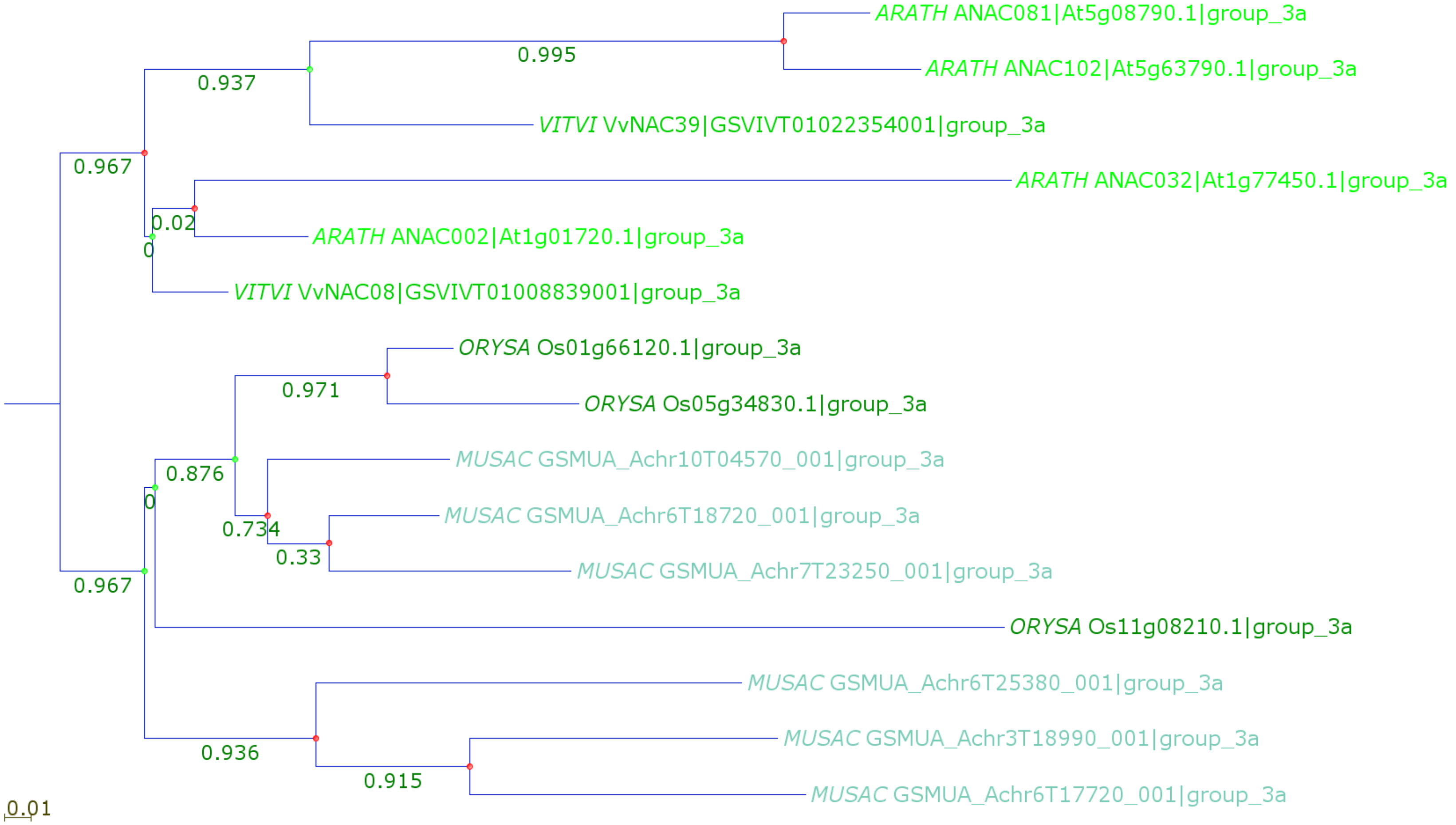
ARATH ANAC081|At5g08790.1|group_3a
ARATH ANAC102|At5g63790.1|group_3a
VITVI VvNAC39|GSVIVT01022354001|group_3a
ARATH ANAC032|At1g77450.1|group_3a
ARATH ANAC002|At1g01720.1|group_3a
VITVI VvNAC08|GSVIVT01008839001|group_3a
ORYSA Os01g66120.1|group_3a
ORYSA Os05g34830.1|group_3a
MUSAC GSMUA_Achr10T04570_001|group_3a
MUSAC GSMUA_Achr6T18720_001|group_3a
MUSAC GSMUA_Achr7T23250_001|group_3a
ORYSA Os11g08210.1|group_3a
MUSAC GSMUA_Achr6T25380_001|group_3a
MUSAC GSMUA_Achr3T18990_001|group_3a
MUSAC GSMUA_Achr6T17720_001|group_3a
0.995
0.937
0.967
0.02
0
0.971
0.876
0
0.734
0.33
0.967
0.936
0.915
0.01

Group 3a

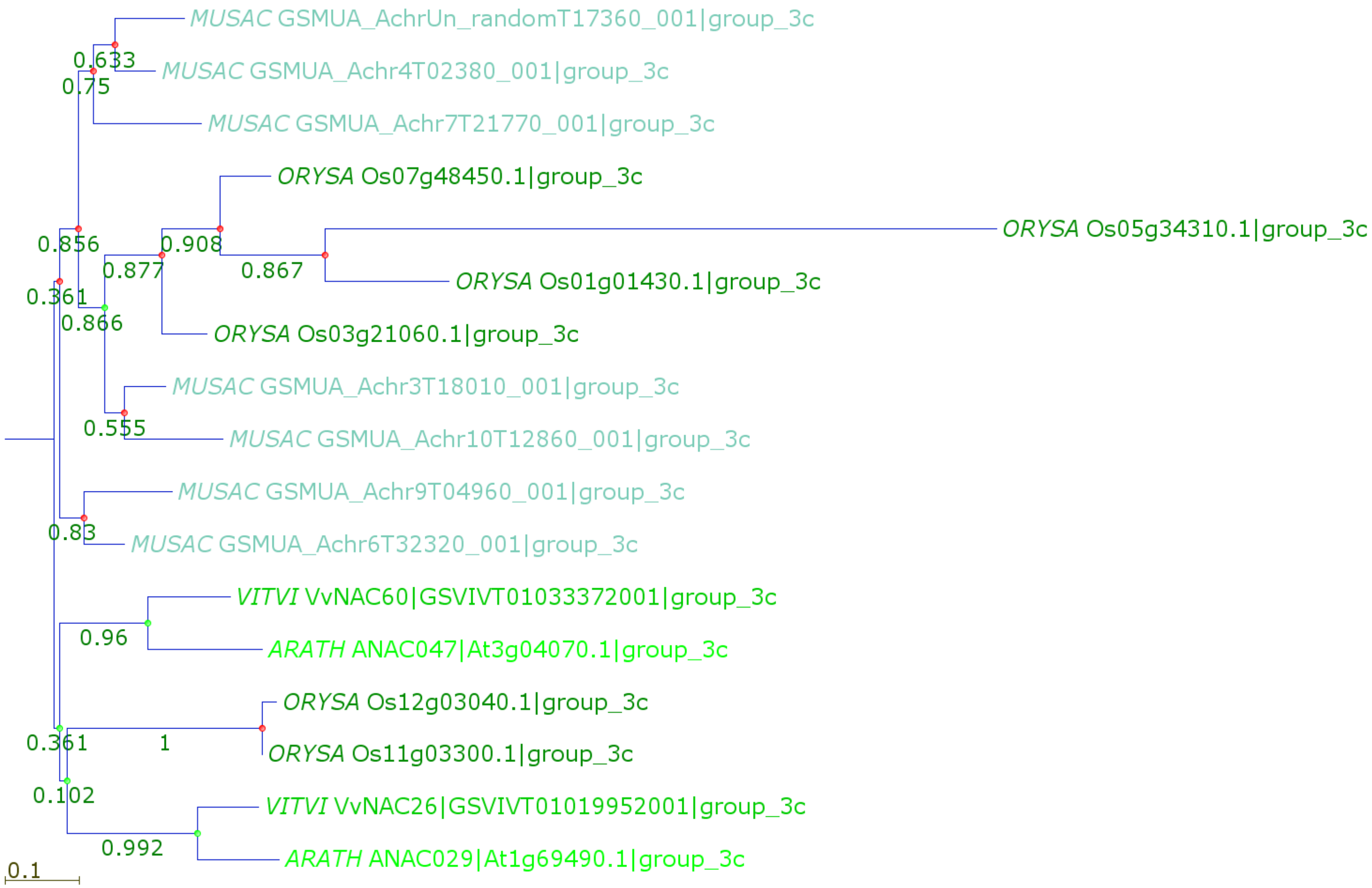
MUSAC GSMUA_AchrUn_randomT17360_001|group_3c
0.633
MUSAC GSMUA_Achr4T02380_001|group_3c
0.75
MUSAC GSMUA_Achr7T21770_001|group_3c
ORYSA Os07g48450.1|group_3c
ORYSA Os05g34310.1|group_3c
0.856
0.908
0.877
0.867
ORYSA Os01g01430.1|group_3c
0.361
0.866
ORYSA Os03g21060.1|group_3c
MUSAC GSMUA_Achr3T18010_001|group_3c
0.555
MUSAC GSMUA_Achr10T12860_001|group_3c
MUSAC GSMUA_Achr9T04960_001|group_3c
0.83
MUSAC GSMUA_Achr6T32320_001|group_3c
VITVI VvNAC60|GSVIVT01033372001|group_3c
0.96
ARATH ANAC047|At3g04070.1|group_3c
ORYSA Os12g03040.1|group_3c
0.361
1
ORYSA Os11g03300.1|group_3c
0.102
VITVI VvNAC26|GSVIVT01019952001|group_3c
0.992
ARATH ANAC029|At1g69490.1|group_3c
0.1

Group 3c

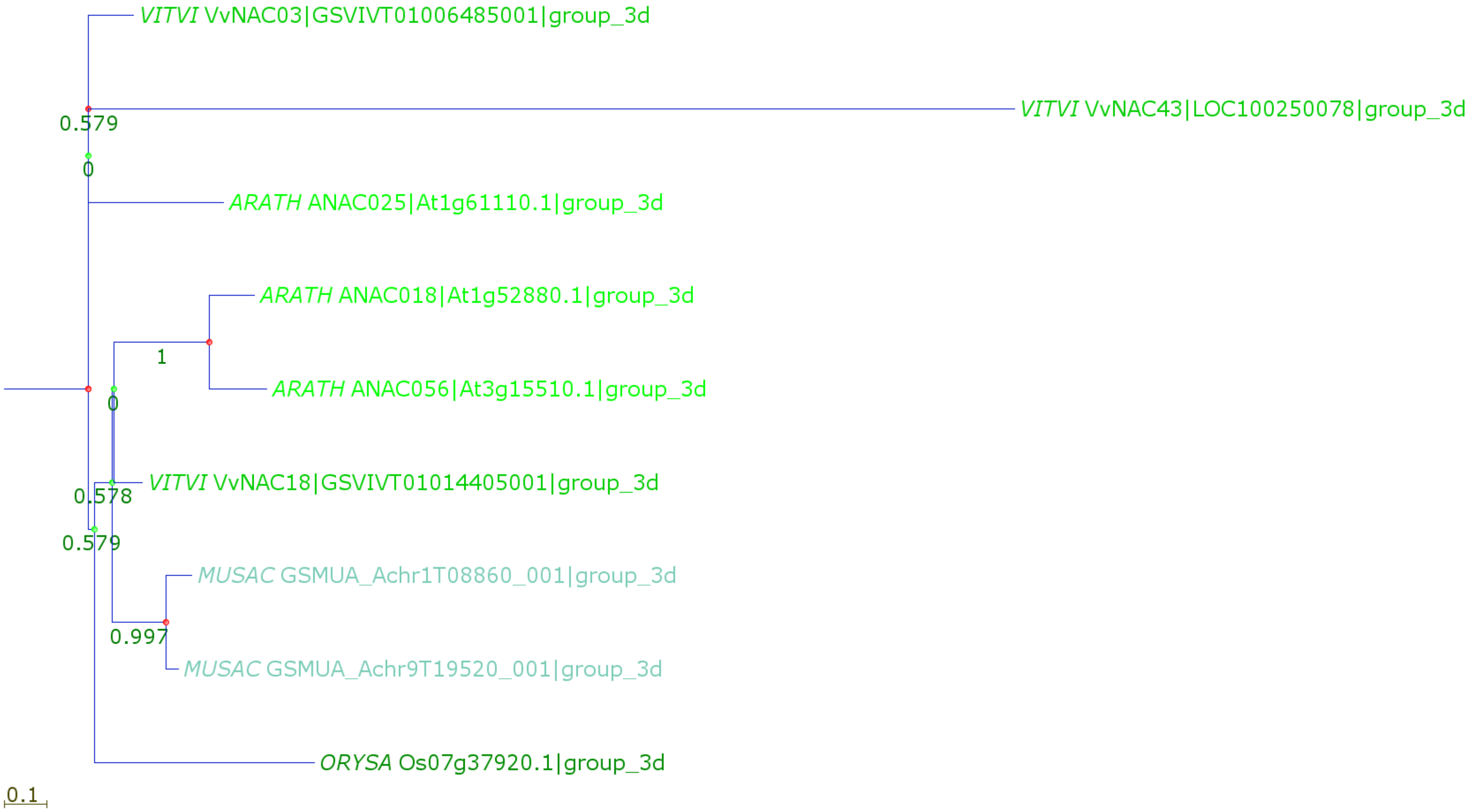
VITVI VvNAC03|GSVIVT01006485001|group_3d
0.579
VITVI VvNAC43|LOC100250078|group_3d
0
ARATH ANAC025|At1g61110.1|group_3d
ARATH ANAC018|At1g52880.1|group_3d
1
ARATH ANAC056|At3g15510.1|group_3d
0
VITVI VvNAC18|GSVIVT01014405001|group_3d
0.578
0.579
MUSAC GSMUA_Achr1T08860_001|group_3d
0.997
MUSAC GSMUA_Achr9T19520_001|group_3d
ORYSA Os07g37920.1|group_3d
0.1

Group 3d

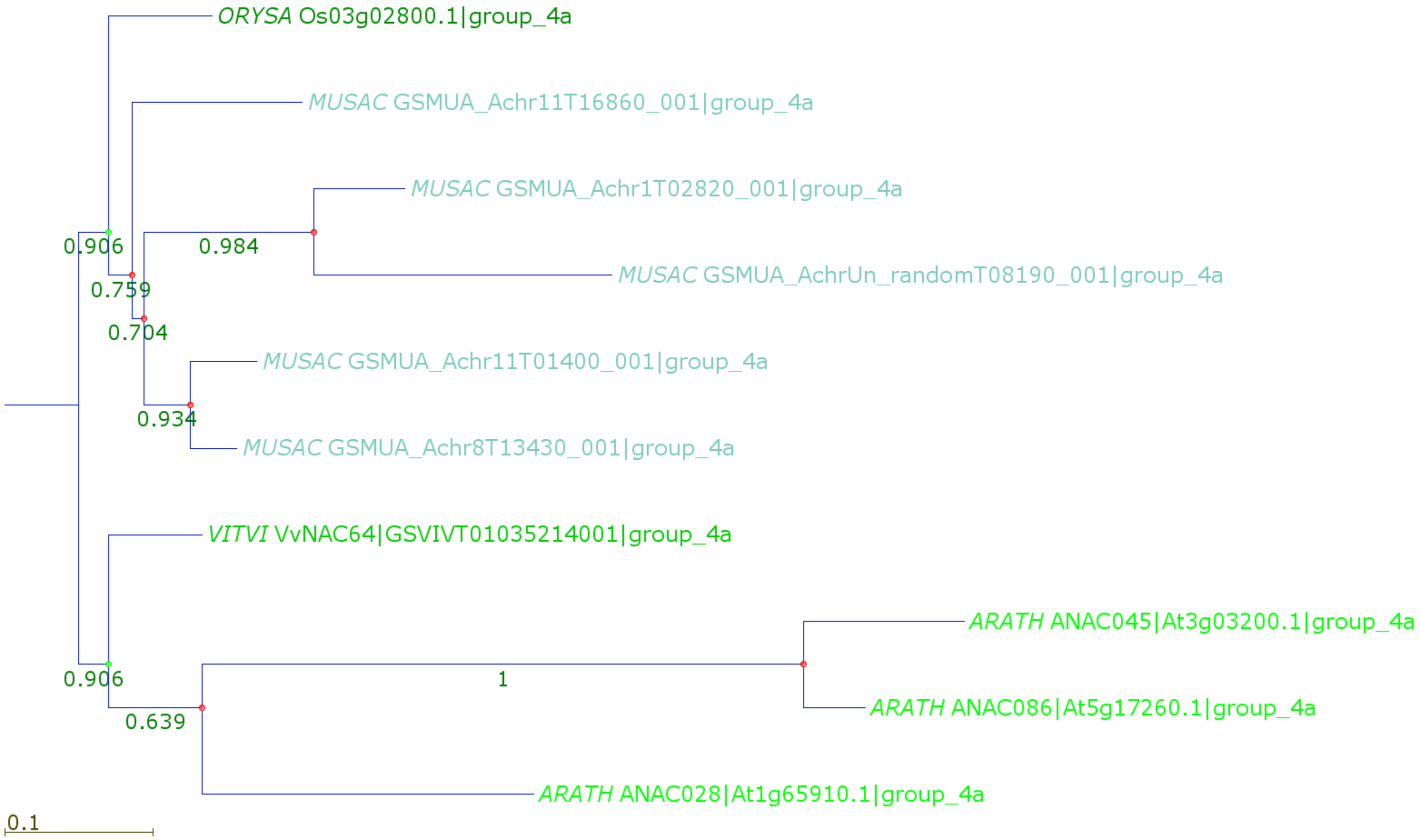
ORYSA Os03g02800.1|group_4a
MUSAC GSMUA_Achr11T16860_001|group_4a
MUSAC GSMUA_Achr1T02820_001|group_4a
0.906
0.984
MUSAC GSMUA_AchrUn_randomT08190_001|group_4a
0.759
0.704
MUSAC GSMUA_Achr11T01400_001|group_4a
0.934
MUSAC GSMUA_Achr8T13430_001|group_4a
VITVI VvNAC64|GSVIVT01035214001|group_4a
ARATH ANAC045|At3g03200.1|group_4a
0.906
1
ARATH ANAC086|At5g17260.1|group_4a
0.639
ARATH ANAC028|At1g65910.1|group_4a
0.1

Group 4a

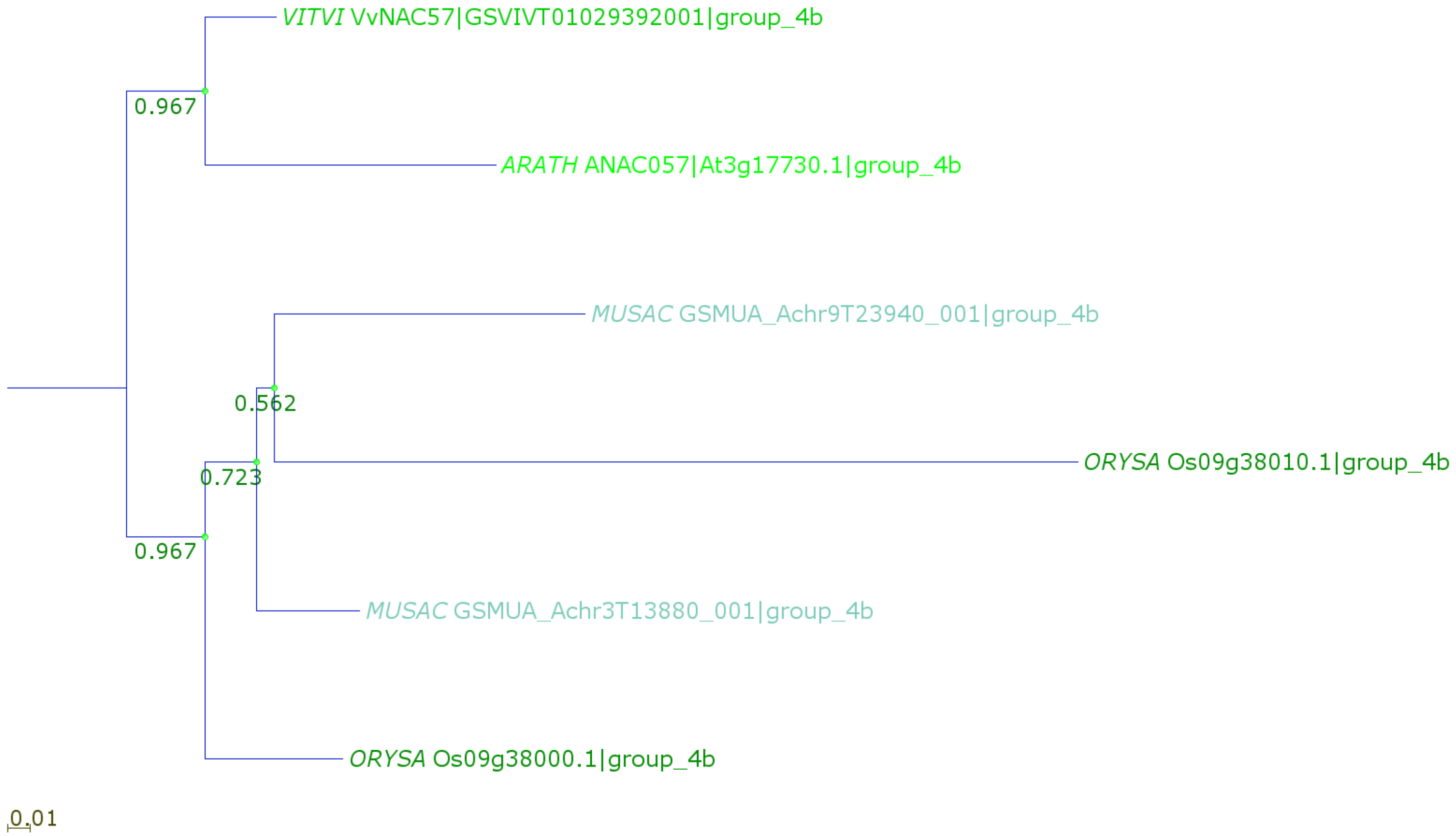
VITVI VvNAC57|GSVIVT01029392001|group_4b
0.967
ARATH ANAC057|At3g17730.1|group_4b
MUSAC GSMUA_Achr9T23940_001|group_4b
0.562
ORYSA Os09g38010.1|group_4b
0.723
0.967
MUSAC GSMUA_Achr3T13880_001|group_4b
ORYSA Os09g38000.1|group_4b
0.01

Group 4b

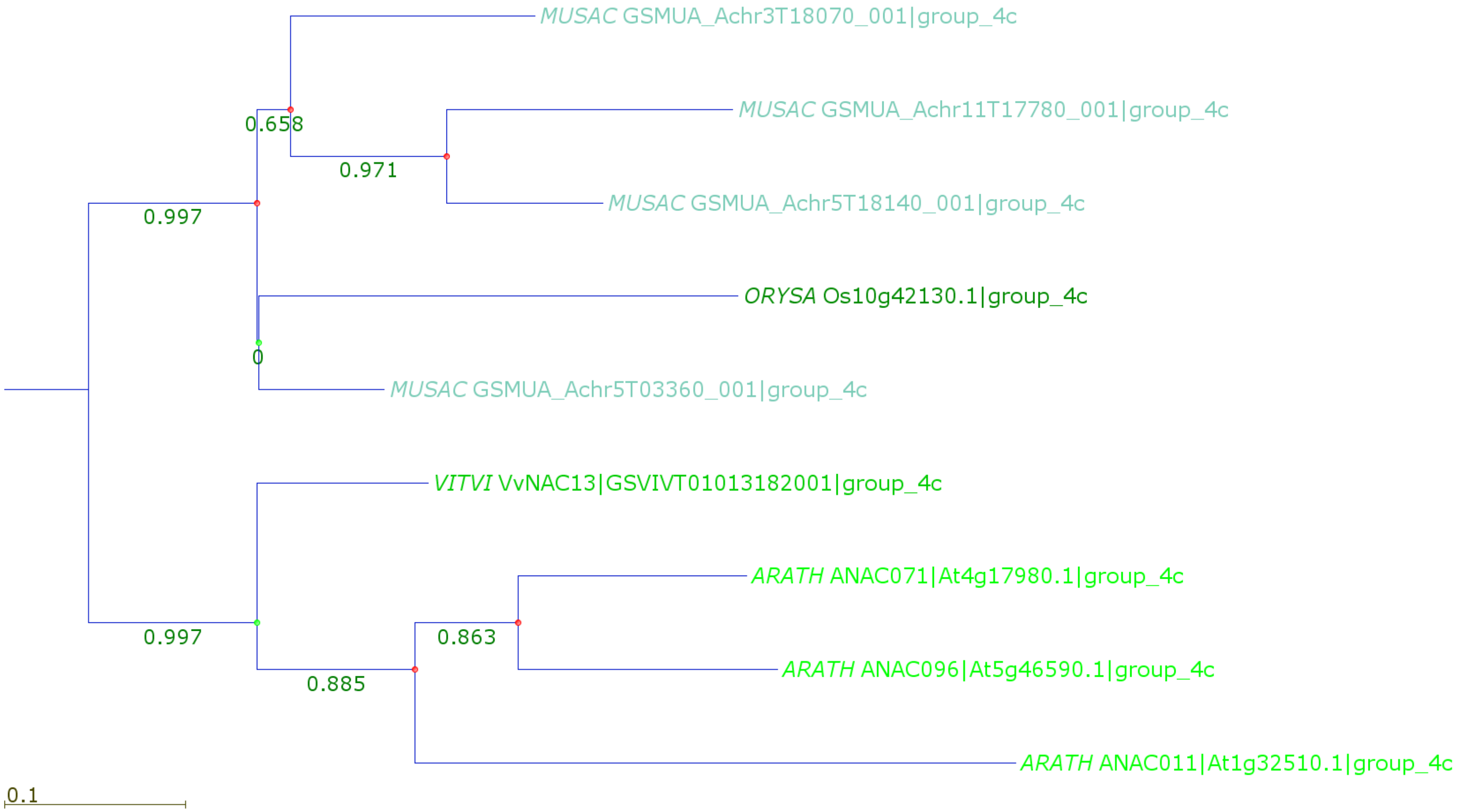
MUSAC GSMUA_Achr3T18070_001|group_4c
0.658
MUSAC GSMUA_Achr11T17780_001|group_4c
0.971
MUSAC GSMUA_Achr5T18140_001|group_4c
0.997
ORYSA Os10g42130.1|group_4c
0
MUSAC GSMUA_Achr5T03360_001|group_4c
VITVI VvNAC13|GSVIVT01013182001|group_4c
ARATH ANAC071|At4g17980.1|group_4c
0.997
0.863
ARATH ANAC096|At5g46590.1|group_4c
0.885
ARATH ANAC011|At1g32510.1|group_4c
0.1

Group 4c

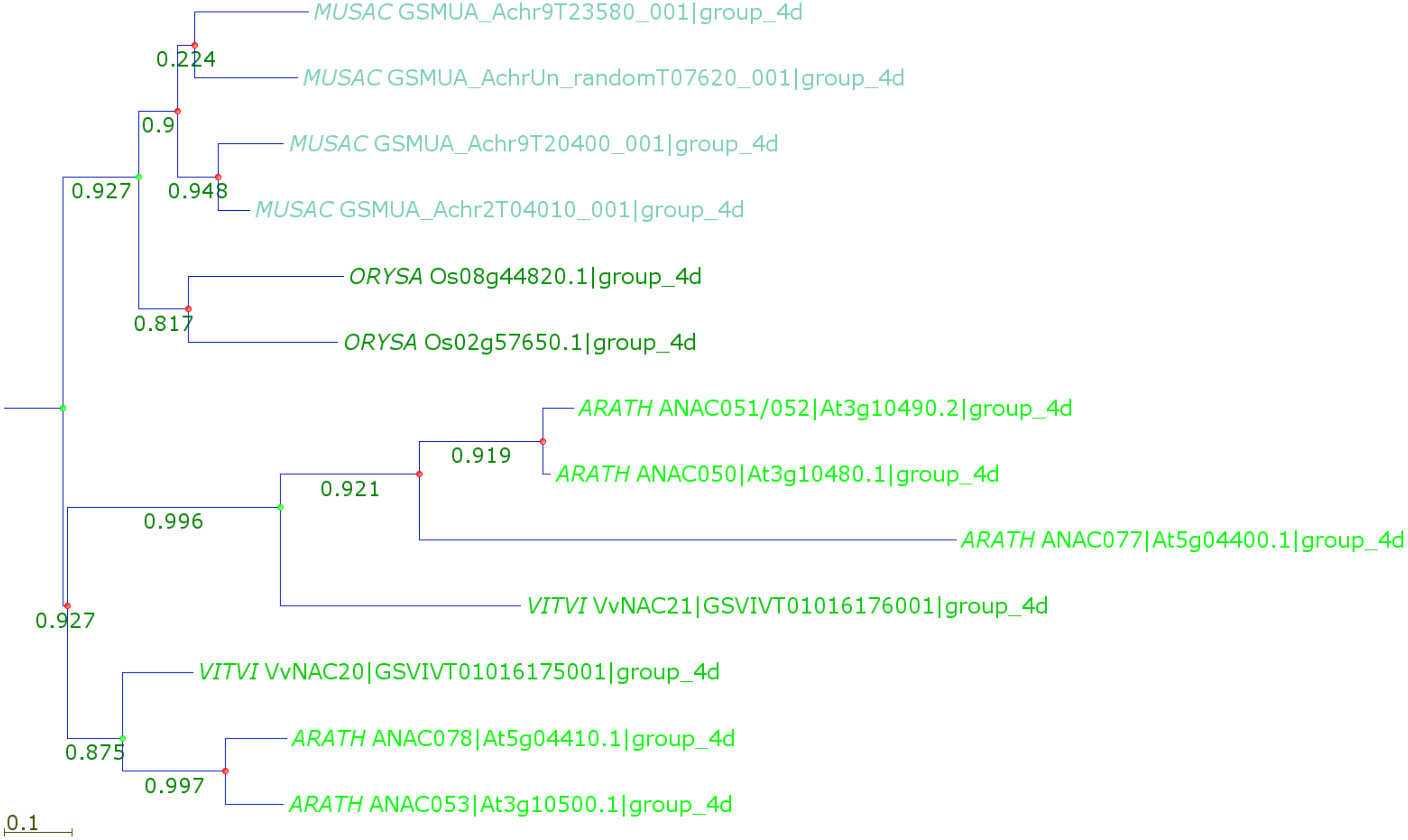
MUSAC GSMUA_Achr9T23580_001|group_4d
0.224
MUSAC GSMUA_AchrUn_randomT07620_001|group_4d
0.9
MUSAC GSMUA_Achr9T20400_001|group_4d
0.927
0.948
MUSAC GSMUA_Achr2T04010_001|group_4d
ORYSA Os08g44820.1|group_4d
0.817
ORYSA Os02g57650.1|group_4d
ARATH ANAC051/052|At3g10490.2|group_4d
0.919
ARATH ANAC050|At3g10480.1|group_4d
0.921
0.996
ARATH ANAC077|At5g04400.1|group_4d
VITVI VvNAC21|GSVIVT01016176001|group_4d
0.927
VITVI VvNAC20|GSVIVT01016175001|group_4d
ARATH ANAC078|At5g04410.1|group_4d
0.875
0.997
ARATH ANAC053|At3g10500.1|group_4d
0.1

Group 4d

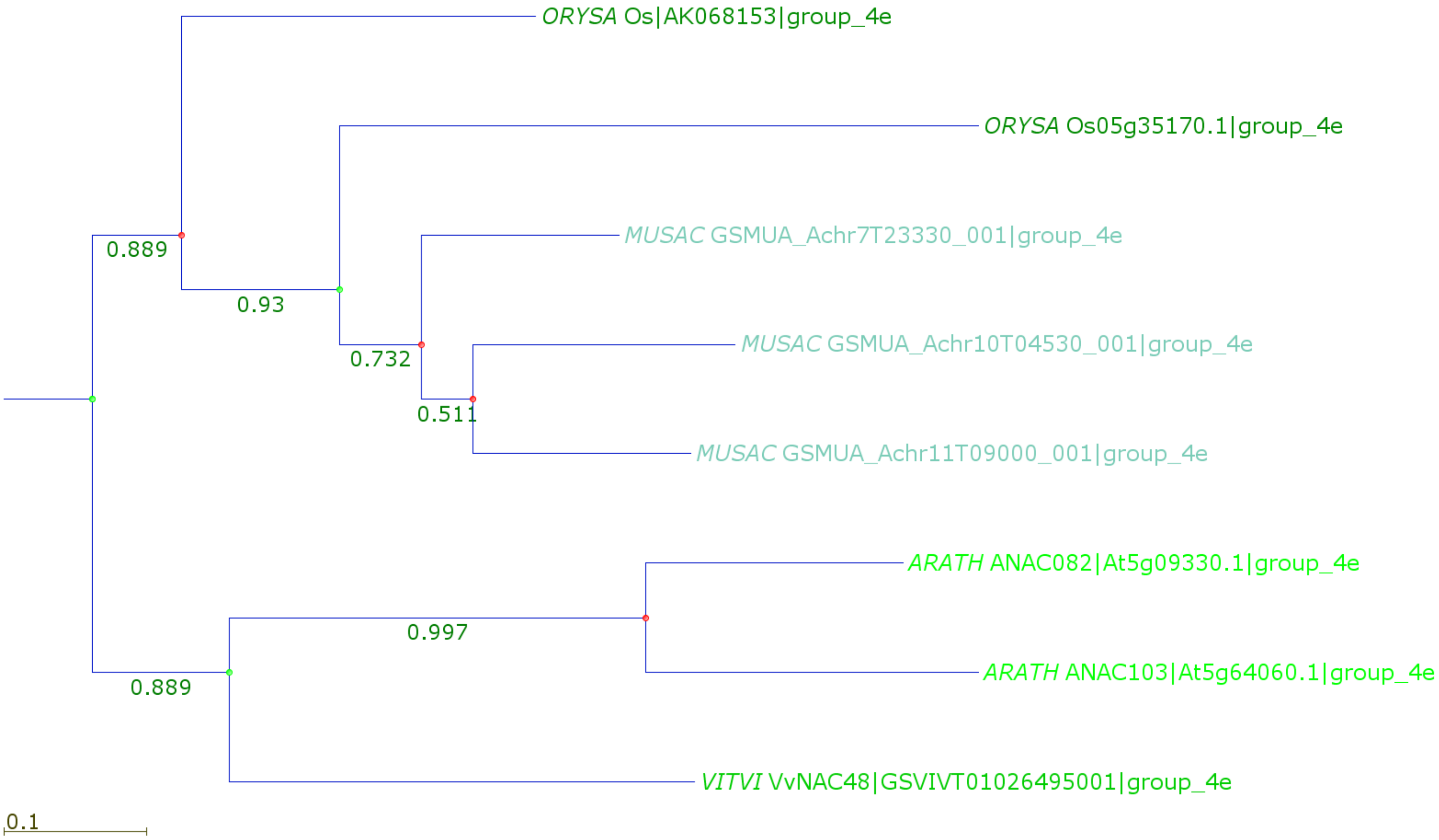

ORYSA Os|AK068153|group_4e
ORYSA Os05g35170.1|group_4e
MUSAC GSMUA_Achr7T23330_001|group_4e
MUSAC GSMUA_Achr10T04530_001|group_4e
MUSAC GSMUA_Achr11T09000_001|group_4e
ARATH ANAC082|At5g09330.1|group_4e
ARATH ANAC103|At5g64060.1|group_4e
VITVI VvNAC48|GSVIVT01026495001|group_4e
0.889
0.93
0.732
0.511
0.997
0.889
0.1

Group 4e

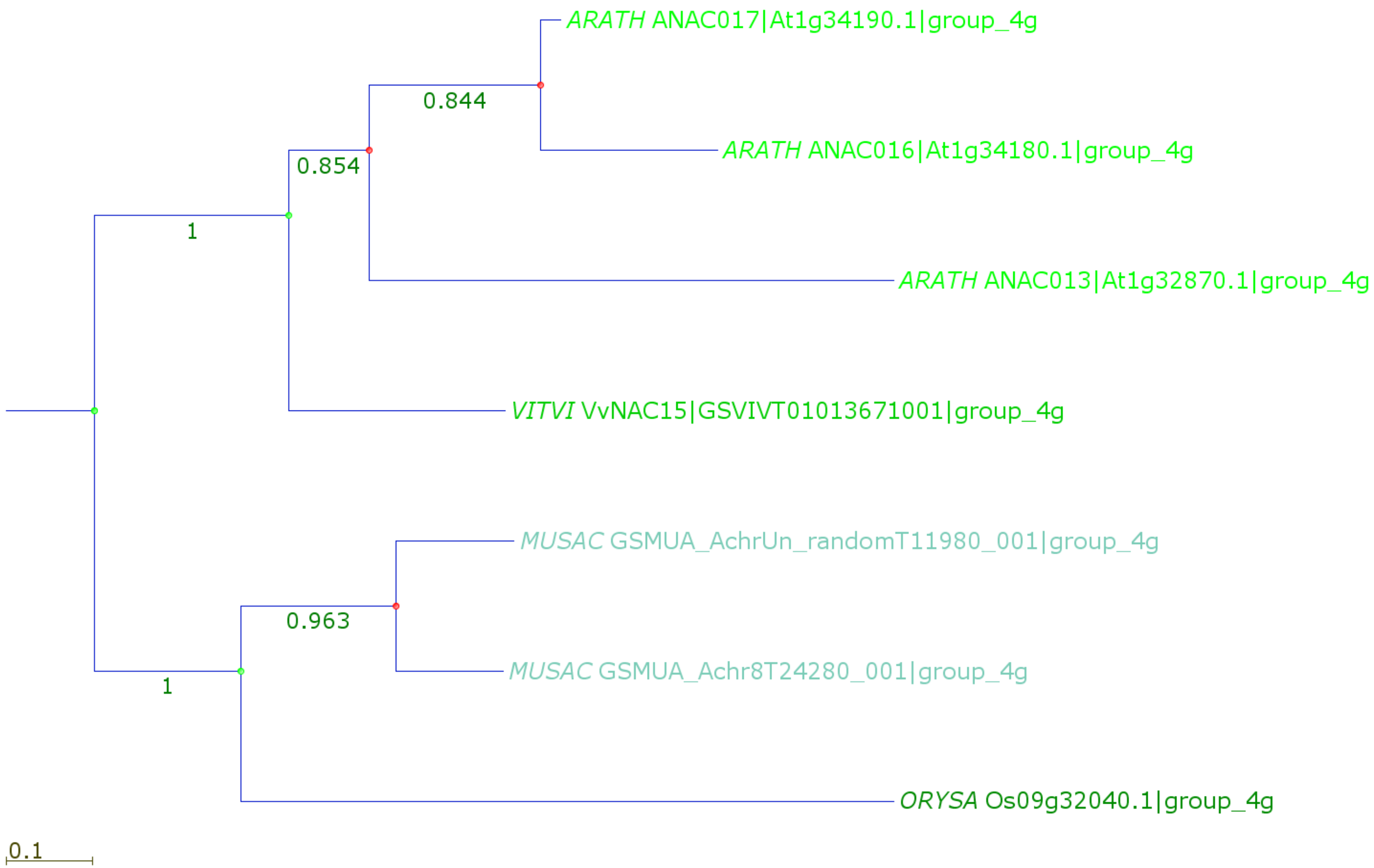
ARATH ANAC017|At1g34190.1|group_4g
ARATH ANAC016|At1g34180.1|group_4g
ARATH ANAC013|At1g32870.1|group_4g
VITVI VvNAC15|GSVIVT01013671001|group_4g
MUSAC GSMUA_AchrUn_randomT11980_001|group_4g
MUSAC GSMUA_Achr8T24280_001|group_4g
ORYSA Os09g32040.1|group_4g
0.844
0.854
1
0.963
1
0.1

Group 4g

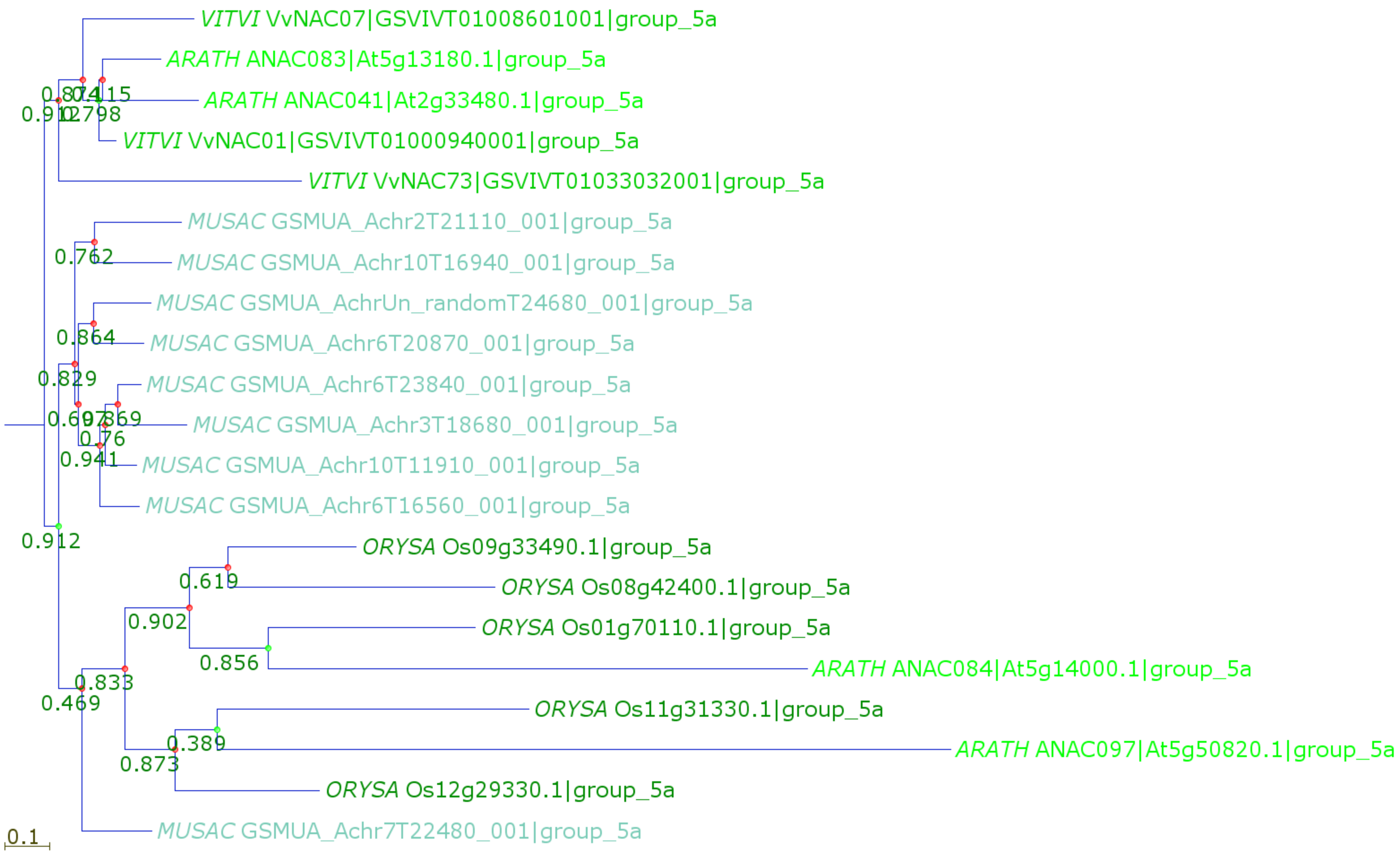
VITVI VvNAC07|GSVIVT01008601001|group_5a
ARATH ANAC083|At5g13180.1|group_5a
ARATH ANAC041|At2g33480.1|group_5a
VITVI VvNAC01|GSVIVT01000940001|group_5a
VITVI VvNAC73|GSVIVT01033032001|group_5a
MUSAC GSMUA_Achr2T21110_001|group_5a
MUSAC GSMUA_Achr10T16940_001|group_5a
MUSAC GSMUA_AchrUn_randomT24680_001|group_5a
MUSAC GSMUA_Achr6T20870_001|group_5a
MUSAC GSMUA_Achr6T23840_001|group_5a
MUSAC GSMUA_Achr3T18680_001|group_5a
MUSAC GSMUA_Achr10T11910_001|group_5a
MUSAC GSMUA_Achr6T16560_001|group_5a
ORYSA Os09g33490.1|group_5a
ORYSA Os08g42400.1|group_5a
ORYSA Os01g70110.1|group_5a
ARATH ANAC084|At5g14000.1|group_5a
ORYSA Os11g31330.1|group_5a
ARATH ANAC097|At5g50820.1|group_5a
ORYSA Os12g29330.1|group_5a
MUSAC GSMUA_Achr7T22480_001|group_5a
0.762
0.864
0.829
0.76
0.941
0.912
0.619
0.902
0.856
0.833
0.469
0.389
0.873
0.1

Group 5a

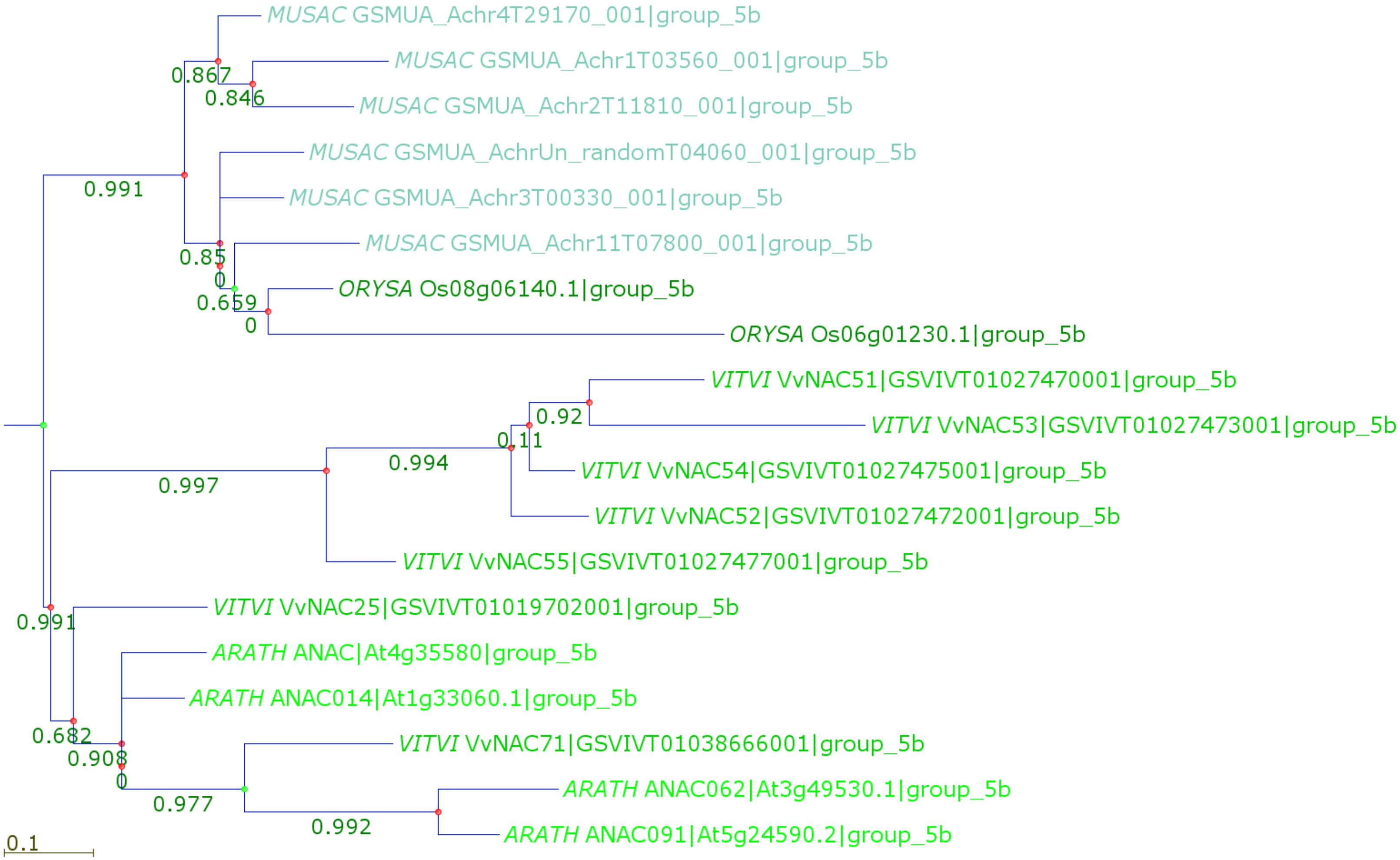
MUSAC GSMUA_Achr4T29170_001|group_5b
MUSAC GSMUA_Achr1T03560_001|group_5b
MUSAC GSMUA_Achr2T11810_001|group_5b
MUSAC GSMUA_AchrUn_randomT04060_001|group_5b
MUSAC GSMUA_Achr3T00330_001|group_5b
MUSAC GSMUA_Achr11T07800_001|group_5b
ORYSA Os08g06140.1|group_5b
ORYSA Os06g01230.1|group_5b
VITVI VvNAC51|GSVIVT01027470001|group_5b
VITVI VvNAC53|GSVIVT01027473001|group_5b
VITVI VvNAC54|GSVIVT01027475001|group_5b
VITVI VvNAC52|GSVIVT01027472001|group_5b
VITVI VvNAC55|GSVIVT01027477001|group_5b
VITVI VvNAC25|GSVIVT01019702001|group_5b
ARATH ANAC|At4g35580|group_5b
ARATH ANAC014|At1g33060.1|group_5b
VITVI VvNAC71|GSVIVT01038666001|group_5b
ARATH ANAC062|At3g49530.1|group_5b
ARATH ANAC091|At5g24590.2|group_5b
0.867
0.846
0.991
0.850
0.659
0
0.92
0.11
0.994
0.997
0.991
0.682
0.908
0
0.977
0.992
0.1

Group 5b

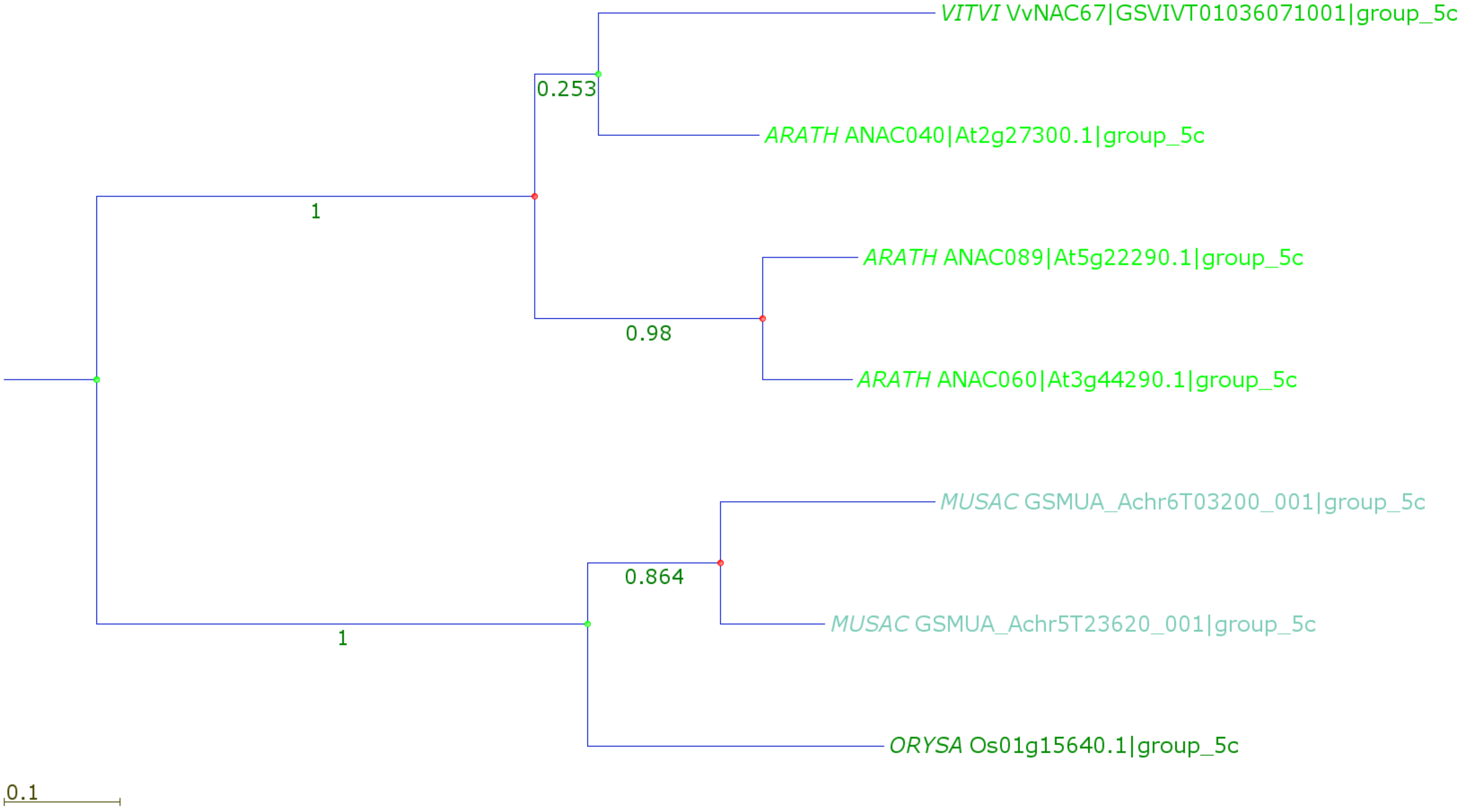

VITVI VvNAC67|GSVIVT01036071001|group_5c
0.253
ARATH ANAC040|At2g27300.1|group_5c
1
ARATH ANAC089|At5g22290.1|group_5c
0.98
ARATH ANAC060|At3g44290.1|group_5c
MUSAC GSMUA_Achr6T03200_001|group_5c
0.864
MUSAC GSMUA_Achr5T23620_001|group_5c
1
ORYSA Os01g15640.1|group_5c
0.1

Group 5c

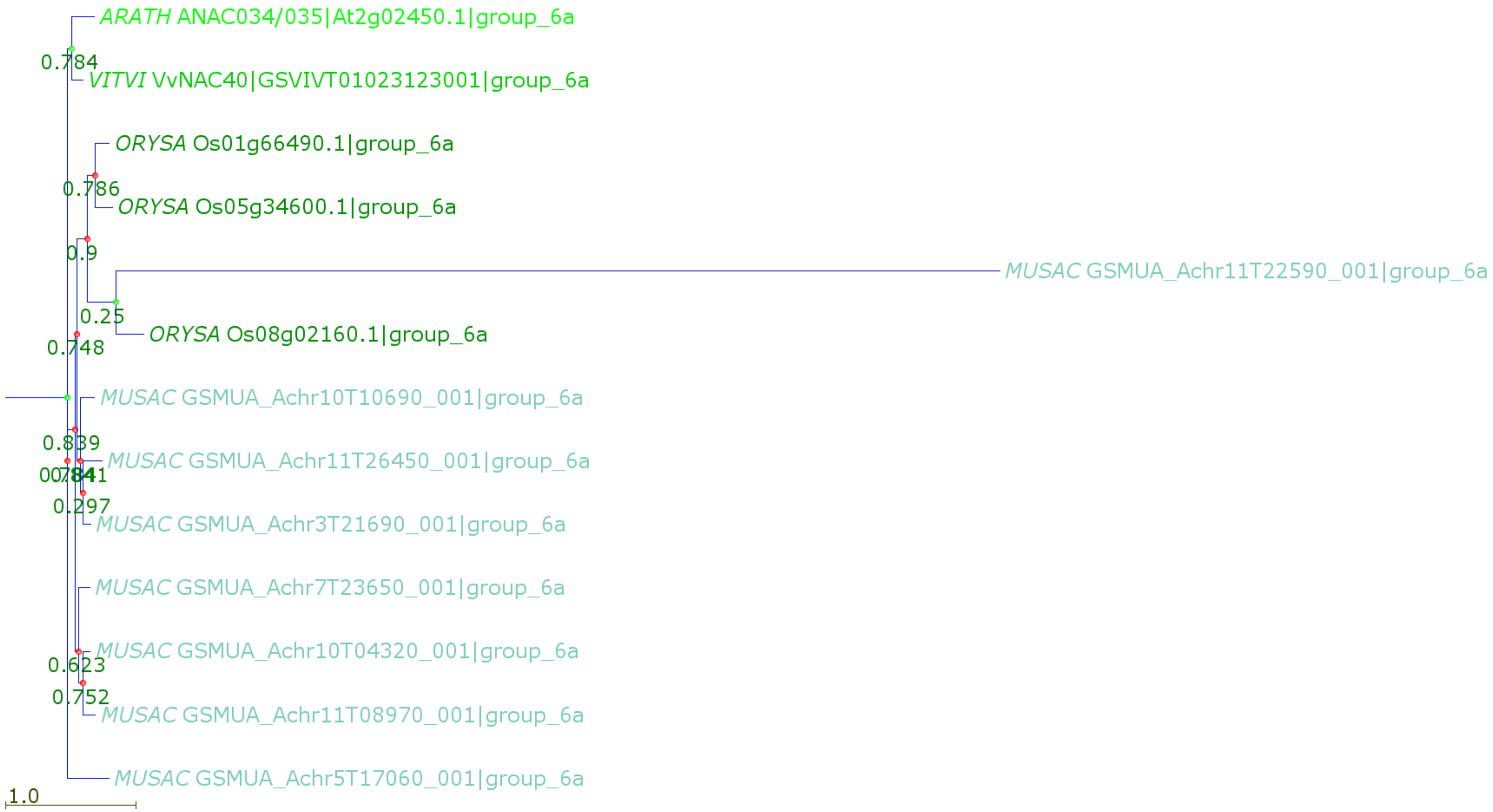
ARATH ANAC034/035|At2g02450.1|group_6a
0.784
VITVI VvNAC40|GSVIVT01023123001|group_6a
ORYSA Os01g66490.1|group_6a
0.786
ORYSA Os05g34600.1|group_6a
0.9
MUSAC GSMUA_Achr11T22590_001|group_6a
0.25
ORYSA Os08g02160.1|group_6a
0.748
MUSAC GSMUA_Achr10T10690_001|group_6a
0.839
MUSAC GSMUA_Achr11T26450_001|group_6a
0.297
MUSAC GSMUA_Achr3T21690_001|group_6a
MUSAC GSMUA_Achr7T23650_001|group_6a
MUSAC GSMUA_Achr10T04320_001|group_6a
0.623
0.752
MUSAC GSMUA_Achr11T08970_001|group_6a
MUSAC GSMUA_Achr5T17060_001|group_6a
1.0

Group 6a

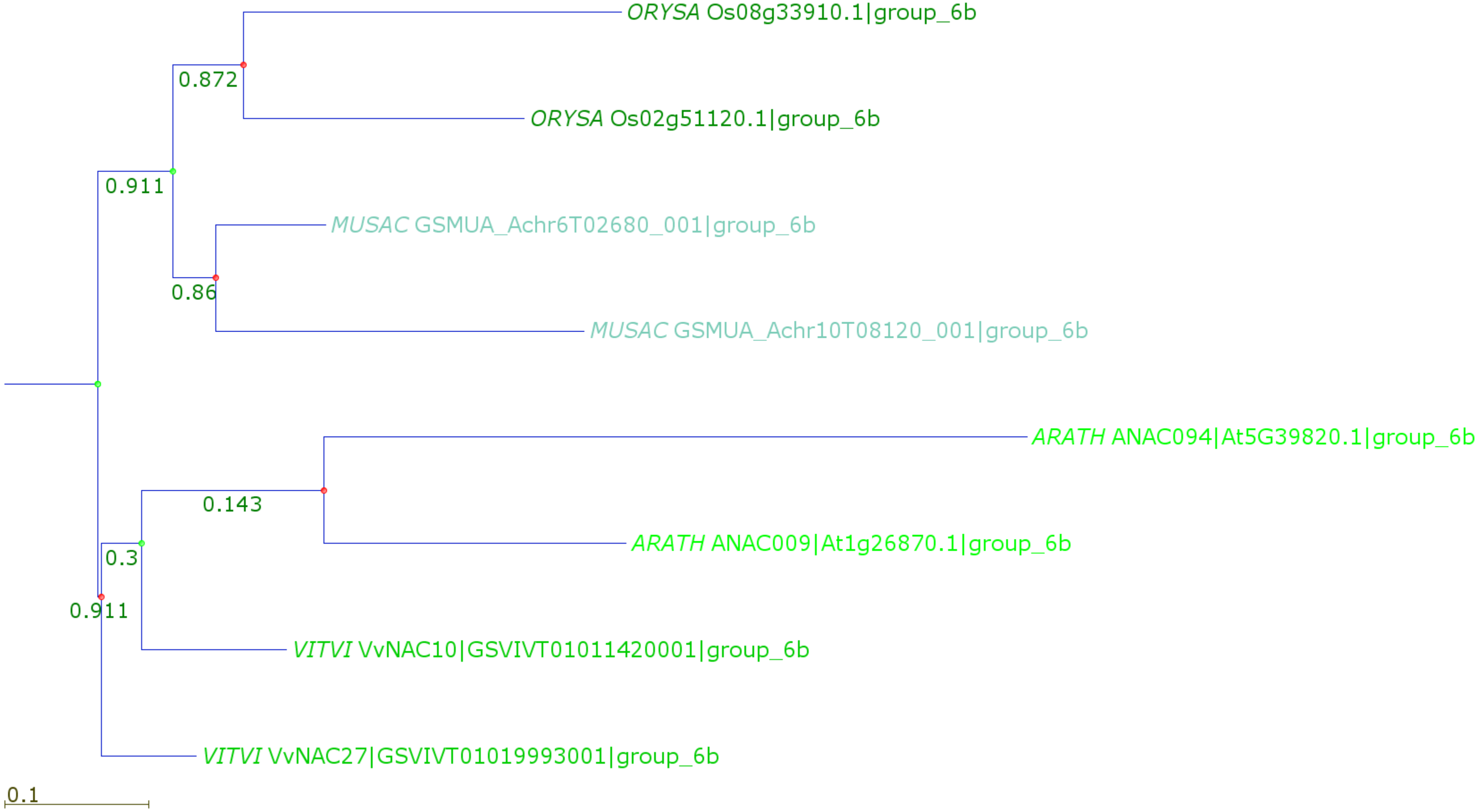

ORYSA Os08g33910.1|group_6b
0.872
ORYSA Os02g51120.1|group_6b
0.911
MUSAC GSMUA_Achr6T02680_001|group_6b
0.86
MUSAC GSMUA_Achr10T08120_001|group_6b
ARATH ANAC094|At5G39820.1|group_6b
0.143
ARATH ANAC009|At1g26870.1|group_6b
0.3
0.911
VITVI VvNAC10|GSVIVT01011420001|group_6b
VITVI VvNAC27|GSVIVT01019993001|group_6b
0.1

Group 6b

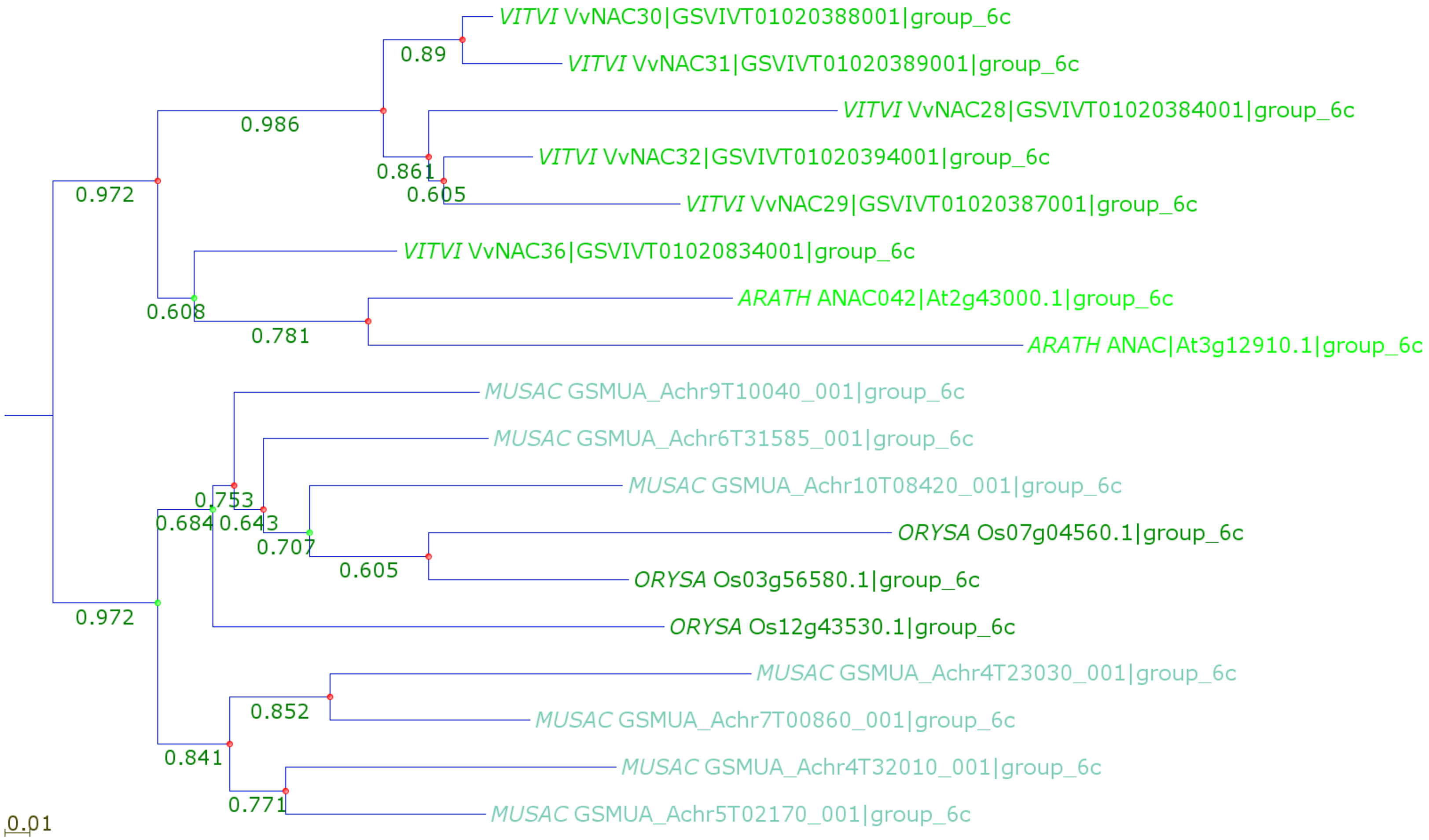
VITVI VvNAC30|GSVIVT01020388001|group_6c
VITVI VvNAC31|GSVIVT01020389001|group_6c
VITVI VvNAC28|GSVIVT01020384001|group_6c
VITVI VvNAC32|GSVIVT01020394001|group_6c
VITVI VvNAC29|GSVIVT01020387001|group_6c
VITVI VvNAC36|GSVIVT01020834001|group_6c
ARATH ANAC042|At2g43000.1|group_6c
ARATH ANAC|At3g12910.1|group_6c
MUSAC GSMUA_Achr9T10040_001|group_6c
MUSAC GSMUA_Achr6T31585_001|group_6c
MUSAC GSMUA_Achr10T08420_001|group_6c
ORYSA Os07g04560.1|group_6c
ORYSA Os03g56580.1|group_6c
ORYSA Os12g43530.1|group_6c
MUSAC GSMUA_Achr4T23030_001|group_6c
MUSAC GSMUA_Achr7T00860_001|group_6c
MUSAC GSMUA_Achr4T32010_001|group_6c
MUSAC GSMUA_Achr5T02170_001|group_6c
0.89
0.986
0.861
0.605
0.972
0.608
0.781
0.753
0.684
0.643
0.707
0.605
0.972
0.852
0.841
0.771
0.01

Group 6c

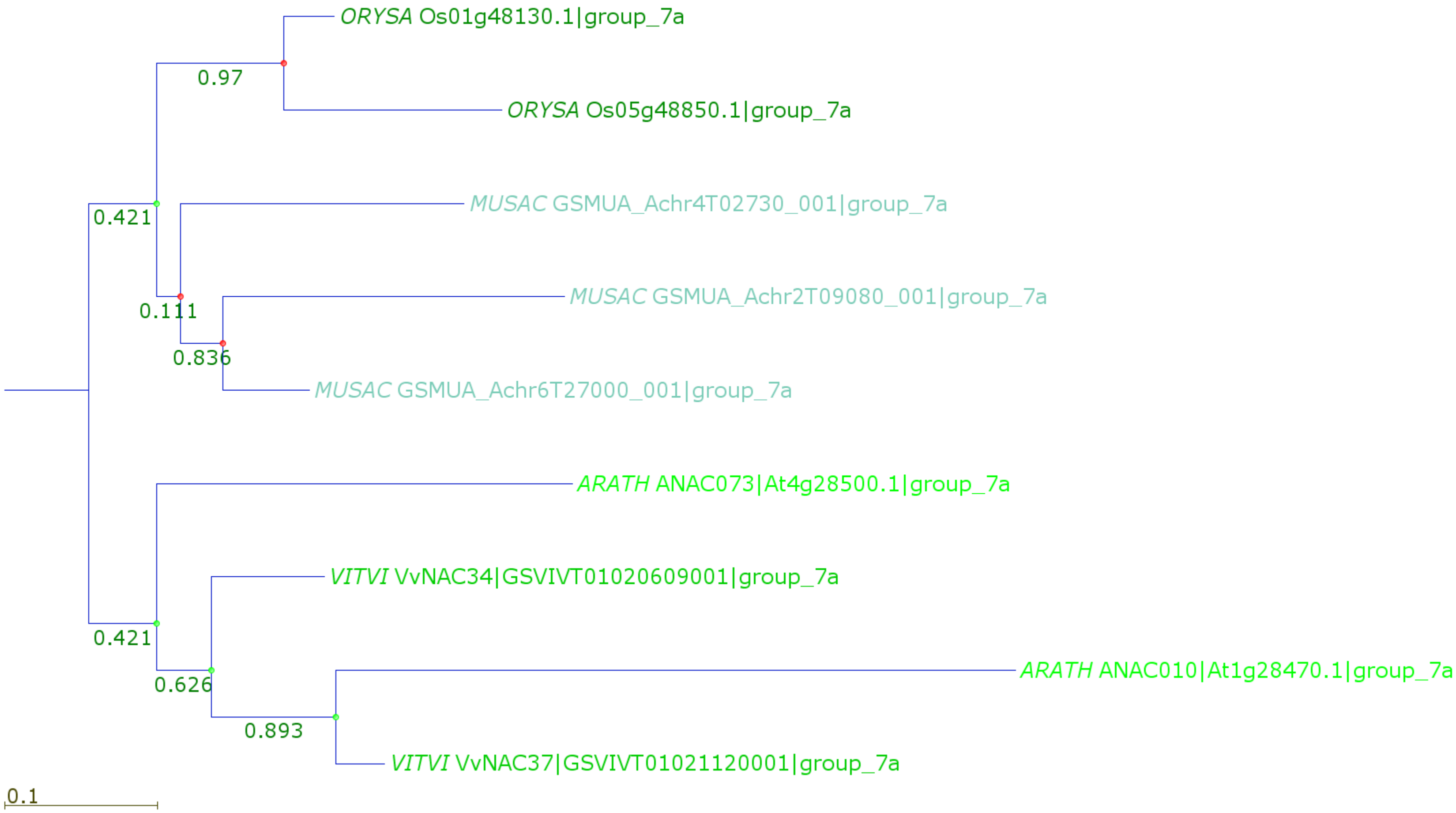
ORYSA Os01g48130.1|group_7a
0.97
ORYSA Os05g48850.1|group_7a
MUSAC GSMUA_Achr4T02730_001|group_7a
0.421
MUSAC GSMUA_Achr2T09080_001|group_7a
0.111
0.836
MUSAC GSMUA_Achr6T27000_001|group_7a
ARATH ANAC073|At4g28500.1|group_7a
VITVI VvNAC34|GSVIVT01020609001|group_7a
ARATH ANAC010|At1g28470.1|group_7a
0.421
0.626
0.893
VITVI VvNAC37|GSVIVT01021120001|group_7a
0.1

Group 7a

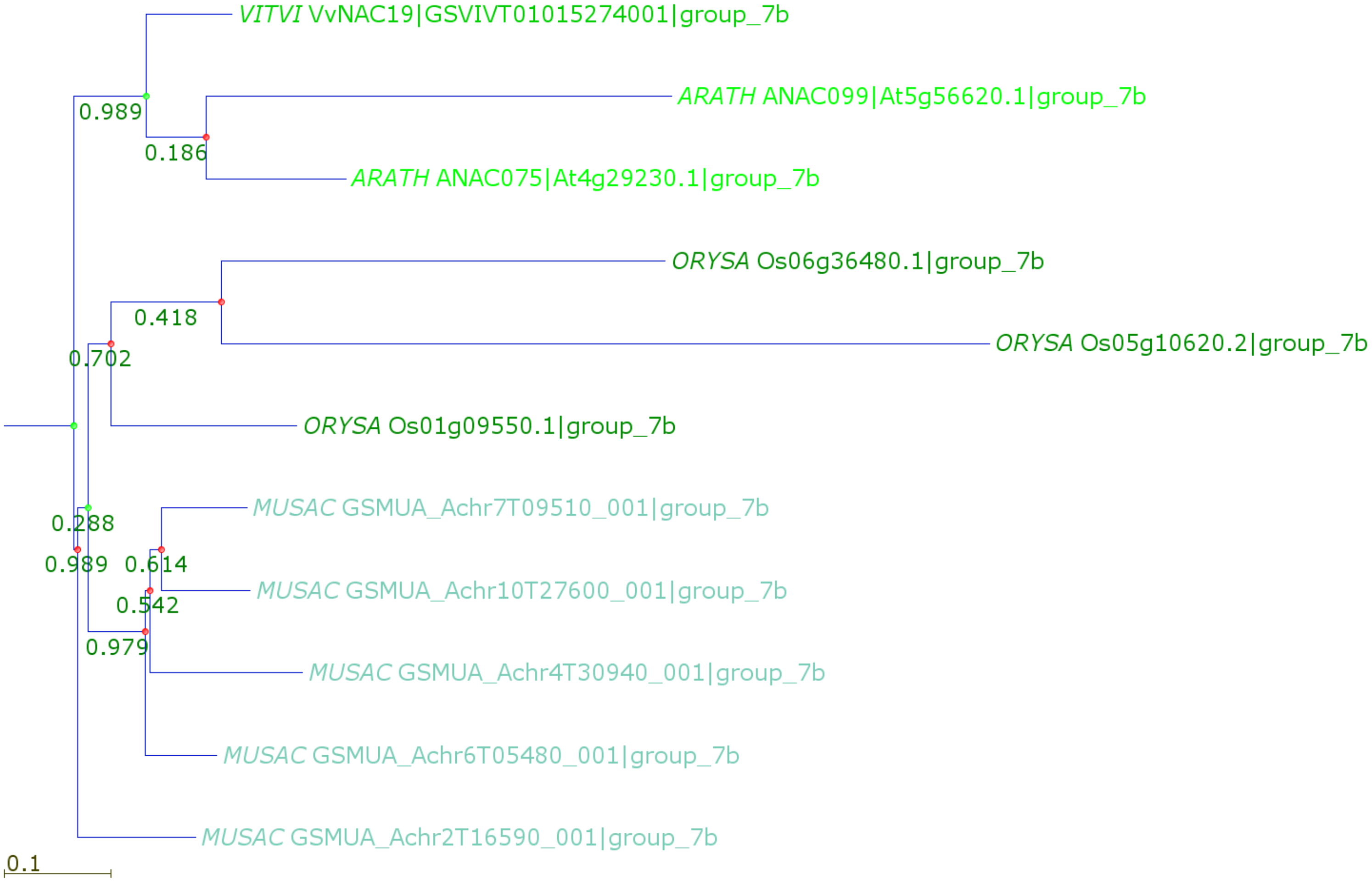

VITVI VvNAC19|GSVIVT01015274001|group_7b
ARATH ANAC099|At5g56620.1|group_7b
ARATH ANAC075|At4g29230.1|group_7b
ORYSA Os06g36480.1|group_7b
ORYSA Os05g10620.2|group_7b
ORYSA Os01g09550.1|group_7b
MUSAC GSMUA_Achr7T09510_001|group_7b
MUSAC GSMUA_Achr10T27600_001|group_7b
MUSAC GSMUA_Achr4T30940_001|group_7b
MUSAC GSMUA_Achr6T05480_001|group_7b
MUSAC GSMUA_Achr2T16590_001|group_7b
0.989
0.186
0.418
0.702
0.288
0.989
0.614
0.542
0.979
0.1

Group 7b

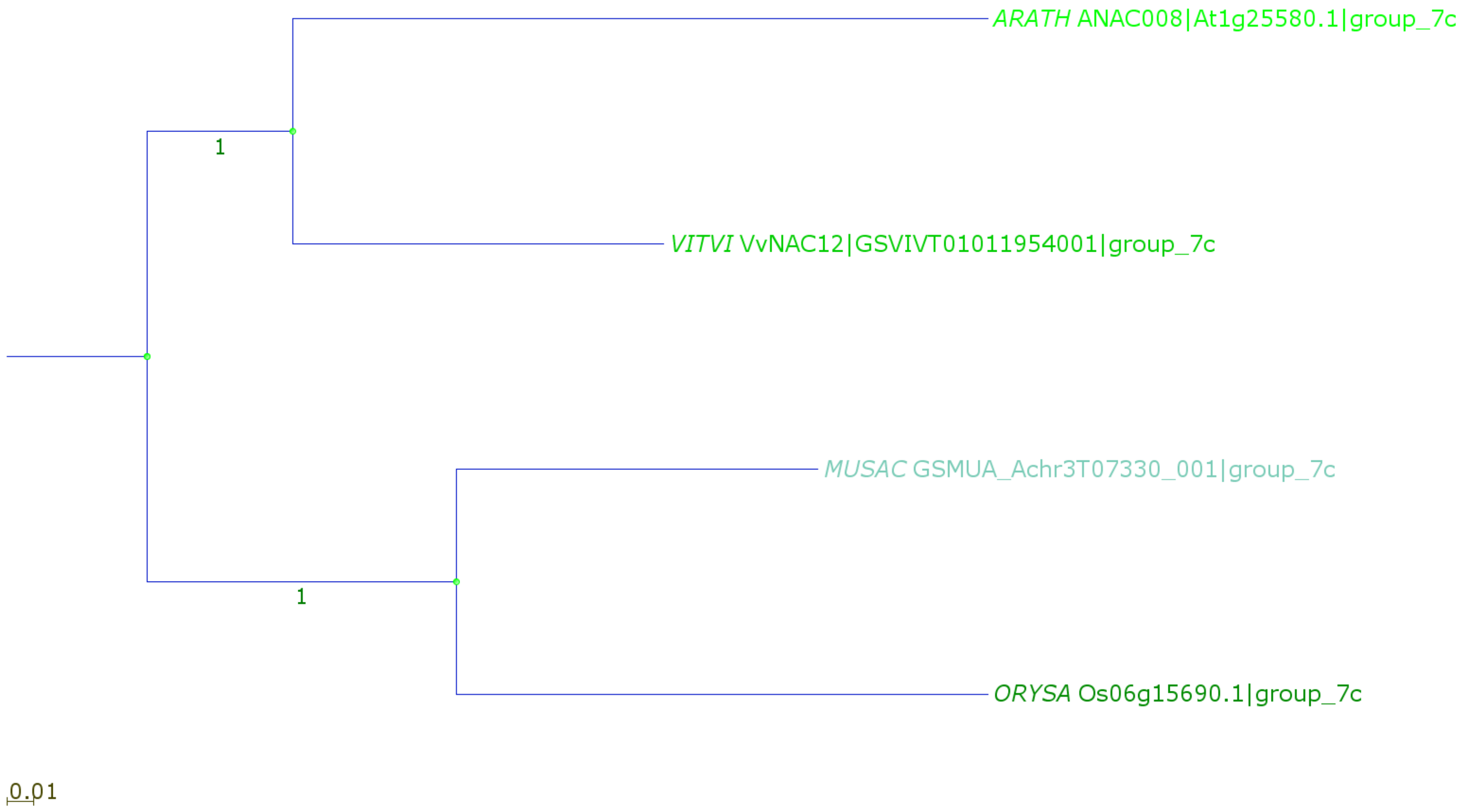
ARATH ANAC008|At1g25580.1|group_7c
1
VITVI VvNAC12|GSVIVT01011954001|group_7c
MUSAC GSMUA_Achr3T07330_001|group_7c
1
ORYSA Os06g15690.1|group_7c
0.01

Group 7c

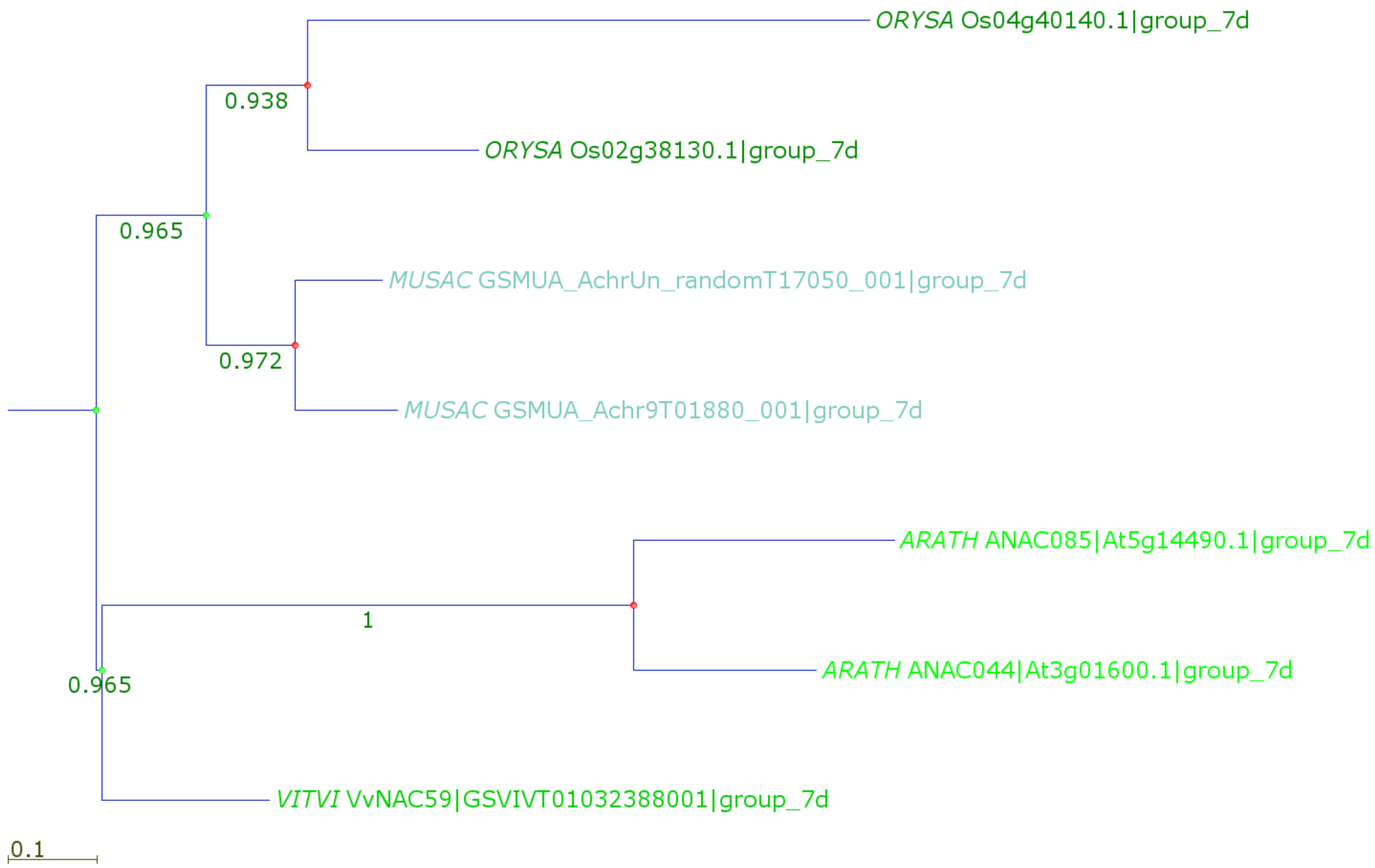

ORYSA Os04g40140.1|group_7d
0.938
ORYSA Os02g38130.1|group_7d
0.965
MUSAC GSMUA_AchrUn_randomT17050_001|group_7d
0.972
MUSAC GSMUA_Achr9T01880_001|group_7d
ARATH ANAC085|At5g14490.1|group_7d
1
0.965
ARATH ANAC044|At3g01600.1|group_7d
VITVI VvNAC59|GSVIVT01032388001|group_7d
0.1

Group 7d

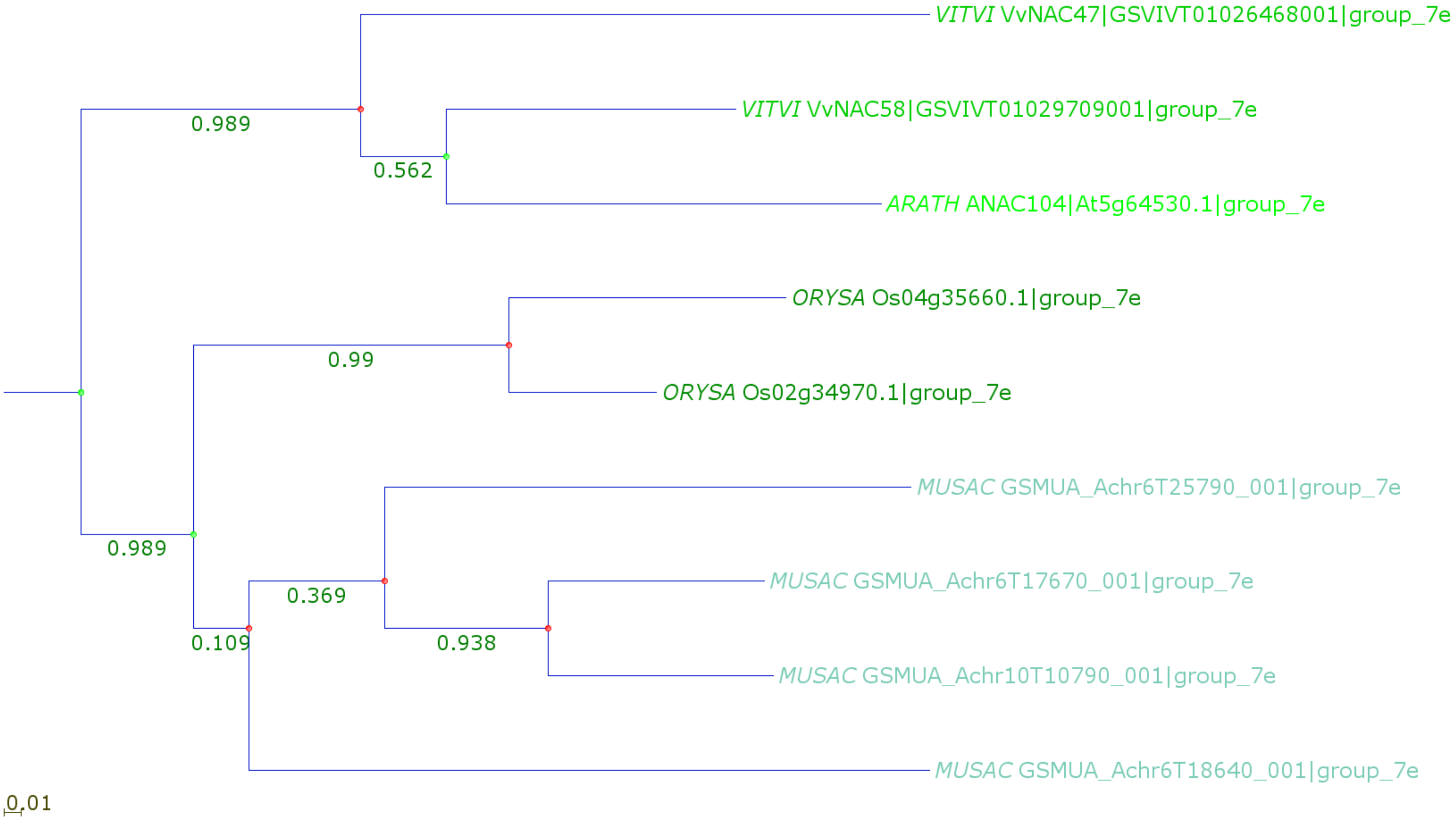
VITVI VvNAC47|GSVIVT01026468001|group_7e
VITVI VvNAC58|GSVIVT01029709001|group_7e
ARATH ANAC104|At5g64530.1|group_7e
ORYSA Os04g35660.1|group_7e
ORYSA Os02g34970.1|group_7e
MUSAC GSMUA_Achr6T25790_001|group_7e
MUSAC GSMUA_Achr6T17670_001|group_7e
MUSAC GSMUA_Achr10T10790_001|group_7e
MUSAC GSMUA_Achr6T18640_001|group_7e
0.989
0.562
0.99
0.989
0.369
0.938
0.109
0.01

Group 7e

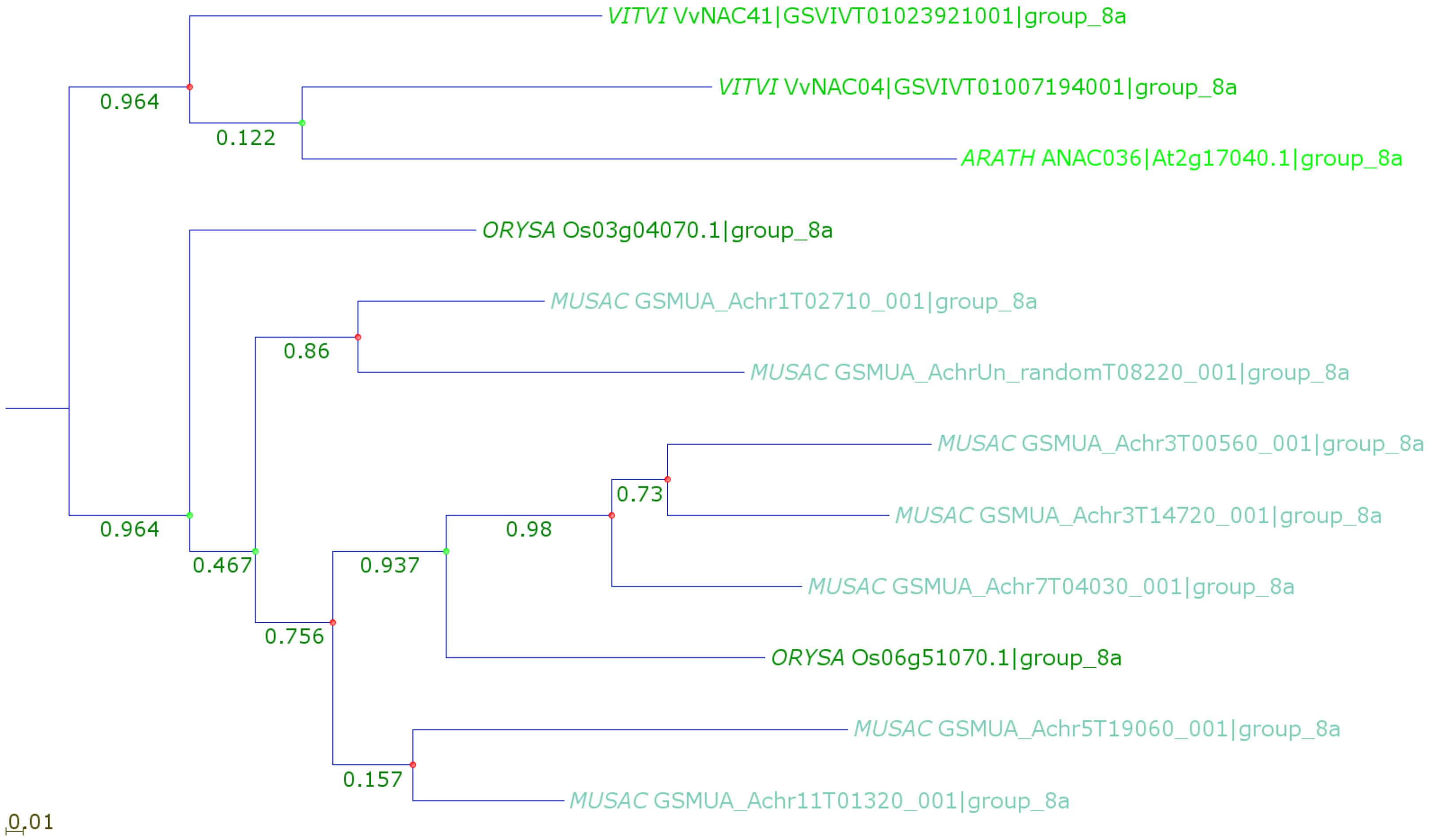
VITVI VvNAC41|GSVIVT01023921001|group_8a
VITVI VvNAC04|GSVIVT01007194001|group_8a
ARATH ANAC036|At2g17040.1|group_8a
ORYSA Os03g04070.1|group_8a
MUSAC GSMUA_Achr1T02710_001|group_8a
MUSAC GSMUA_AchrUn_randomT08220_001|group_8a
MUSAC GSMUA_Achr3T00560_001|group_8a
MUSAC GSMUA_Achr3T14720_001|group_8a
MUSAC GSMUA_Achr7T04030_001|group_8a
ORYSA Os06g51070.1|group_8a
MUSAC GSMUA_Achr5T19060_001|group_8a
MUSAC GSMUA_Achr11T01320_001|group_8a
0.964
0.122
0.964
0.86
0.467
0.73
0.98
0.937
0.756
0.157
0.01

Group 8a

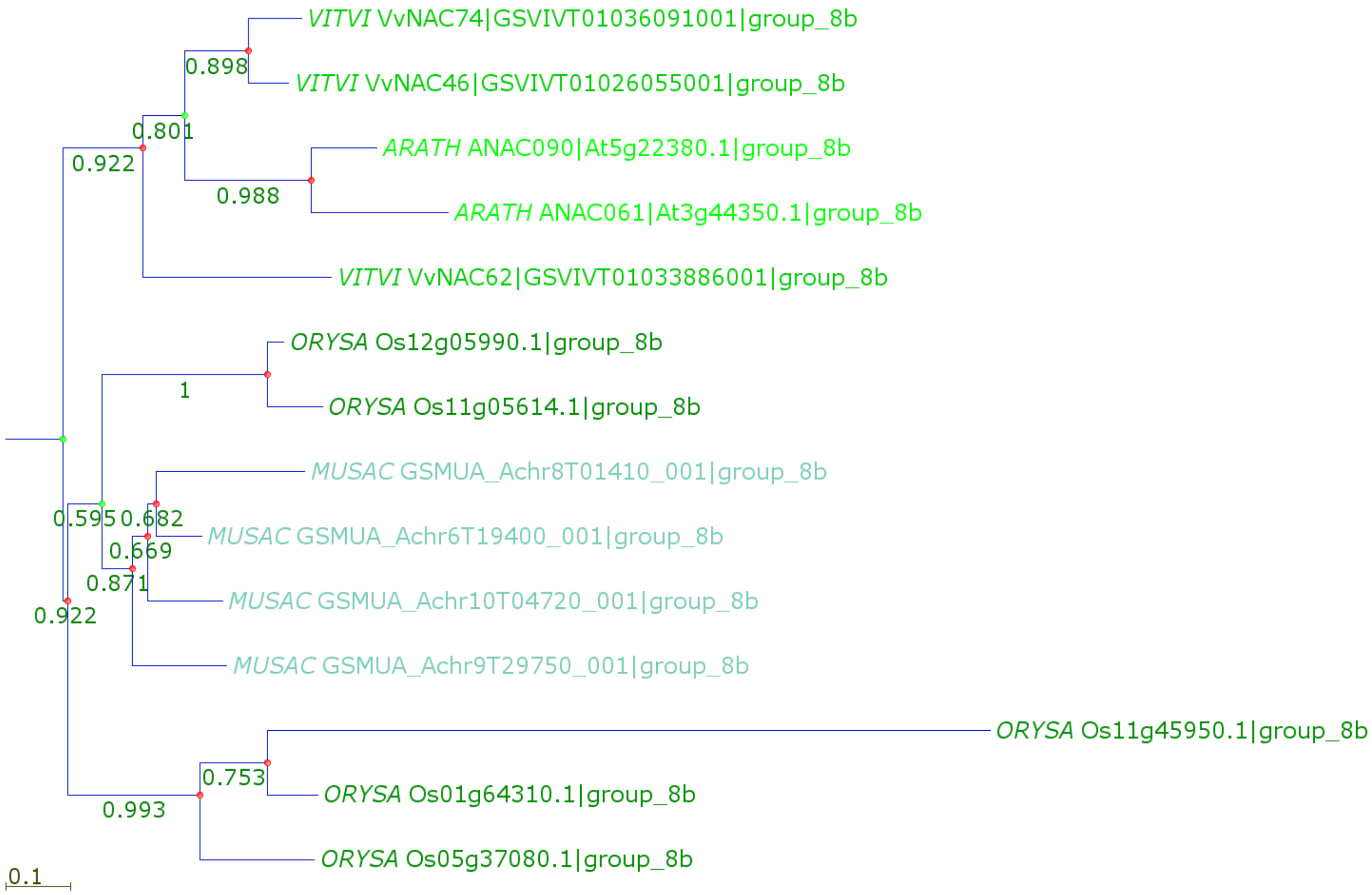
VITVI VvNAC74|GSVIVT01036091001|group_8b
0.898
VITVI VvNAC46|GSVIVT01026055001|group_8b
0.801
ARATH ANAC090|At5g22380.1|group_8b
0.922
0.988
ARATH ANAC061|At3g44350.1|group_8b
VITVI VvNAC62|GSVIVT01033886001|group_8b
ORYSA Os12g05990.1|group_8b
1
ORYSA Os11g05614.1|group_8b
MUSAC GSMUA_Achr8T01410_001|group_8b
0.595
0.682
MUSAC GSMUA_Achr6T19400_001|group_8b
0.669
0.871
MUSAC GSMUA_Achr10T04720_001|group_8b
0.922
MUSAC GSMUA_Achr9T29750_001|group_8b
ORYSA Os11g45950.1|group_8b
0.753
ORYSA Os01g64310.1|group_8b
0.993
ORYSA Os05g37080.1|group_8b
0.1

Group 8b
